# The repeatome landscape in the “*Saccharum* complex”

**DOI:** 10.3389/fpls.2026.1809735

**Published:** 2026-05-21

**Authors:** Nina Reis Soares, Zirlane Portugal da Costa, Luiz Augusto Cauz-Santos, Monalisa Sampaio Carneiro, Maria Lucia Carneiro Vieira

**Affiliations:** 1 Departamento de Genética, Escola Superior de Agricultura “Luiz de Queiroz”, Universidade de São Paulo, Piracicaba, São Paulo, Brazil; 2 Department of Botany and Biodiversity Research, University of Vienna, Vienna, Austria; 3 Departamento de Biotecnologia e Produção Vegetal e Animal, Centro de Ciências Agrárias, Universidade Federal de São Carlos, Araras, São Paulo, Brazil

**Keywords:** phylogeny, polyploid, RepeatExplorer2, repetitive DNA, sugarcane

## Abstract

**Introduction:**

The “*Saccharum* complex” comprises several closely related genera (*Tripidium/Erianthus*, *Miscanthus* and *Narenga*) which may hybridize with *Saccharum*, contributing to the origin of modern sugarcane. Sugarcane (*Saccharum* spp.) has one of the most complex crop genomes, shaped by interspecific hybridization, extreme polyploidy, and extensive chromosomal rearrangements. Research has found that repetitive DNA constitutes a large fraction of its genome. However, the dynamics, distribution, and evolutionary significance of these elements across the “*Saccharum* complex” are yet to be resolved.

**Methods:**

Here, we analyzed data from 30 genotypes, representing nine *Saccharum* species, several modern sugarcane cultivars and individuals from *Erianthus, Miscanthus* and *Narenga*. Repetitive sequences were identified using RepeatExplorer2. Repeat lineage evolution was assessed through comparative clustering, correlation analyses, and repeat-based phylogenetic reconstruction. LTR retrotransposons (LTR-RTs) were further examined in fully assembled genomes of *S. officinarum*, *S. spontaneum*, and the cultivar R570 using DANTE.

**Results:**

Repetitive DNA content ranged from 42.5% to 59.7%, with LTR-RTs the dominant fraction. Ty3 LTR-RTs, particularly Tekay, and Ty1 LTR-RTs, mainly SIRE, exhibited most interspecific variation. A homogeneous repeatome was found in *S. officinarum*, while *S. spontaneum* exhibited divergence among cytotypes. Satellite DNAs were abundant and largely taxon-specific, yet all species shared a highly conserved 137-bp centromeric repeat. Repeat abundance was strongly correlated with genome size, underscoring the central role of transposable element proliferation in genome expansion. Repeat-based phylogenies are (mainly) in line with published *Saccharum* phylogenies. Structural analyses of LTR-RTs revealed lineage-specific signatures of recent amplification, especially within SIRE and Tekay.

**Conclusion:**

Together, these results show that the *Saccharum* repeatome reflects both shared ancestry and extensive lineage-specific diversification. Repetitive DNA has played a major role in genome expansion and retains signatures associated with species differentiation and hybridization. The repeat families identified here provide valuable cytogenetic and genomic resources and offer an evolutionary framework which is key to understanding polyploid genome dynamics in sugarcane.

## 1 Introduction

Cultivated sugarcane (*Saccharum* spp., Poaceae) is the most extensively grown sugar-producing crop worldwide and functions as a renewable feedstock for bioethanol production (Yang et al., 2019). The genus *Saccharum* includes six species: *S. officinarum*, *S. sinense*, *S. barberi*, *S. edule*, *S. robustum*, and *S. spontaneum*. Among these, *S. spontaneum* (2n = 8x, 9x, 10x = 40 – 128) and *S. robustum* (2n = 6x = 60; 2n = 8x = 80) are wild (Irvine, 1999). The cultivated species *S. officinarum* (2n = 8x = 80), or ‘noble cane’, known for its high sugar content, is believed to have originated from the domestication of *S. robustum* (D’Hont et al., 1993; Lu et al., 1994; Schenck, 2004). *S. sinense* and *S. barberi* are considered hybrids between *S. officinarum* and *S. spontaneum* (D’Hont et al., 2002). *S. edule* may have derived from *S. robustum* (Lennoux, 1937), or be a hybrid of *S. officinarum*, *S. robustum*, and possibly other *Saccharum* species (Daniels and Roach, 1987). The foundational species of the *Saccharum* genus are *S. officinarum, S. spontaneum*, and *S. robustum* (Zhang et al., 2019). Genomic analyses suggest that *S. spontaneum* and *S. officinarum* diverged approximately 1.5–2 million years ago (Jannoo et al., 2007; Pompidor et al., 2021).

In addition to *Saccharum*, related genera such as *Erianthus*, *Miscanthus* and *Narenga*, part of the “*Saccharum* complex”, are of growing interest in sugarcane breeding. However, compared to *Saccharum*, genomic and sequence data for these related taxa remain scarce, limiting research on their phylogenetic relationships, population structure, and evolutionary history (Tsuruta et al., 2017).

In the late 19th century, the first sugarcane varieties emerged from interspecific hybridization of previously high sugar cultivated species (*S. barberi* and *S. officinarum*) and wild *S. spontaneum*. *Saccharum spontaneum* was incorporated due to its special attributes, such as hardiness, disease resistance, and vigorous tillering and ratooning capabilities, traits critical for enhancing productivity and resilience. It also has the greatest phenotypic variability within the “*Saccharum* complex” (see Barreto et al., 2021; Cheavegatti-Gianotto et al., 2011; Grivet et al., 2004). The interspecific hybrids were then successively backcrossed with *S. officinarum* to restore their sucrose content, through a process known as ‘nobilization’. During these backcrosses, unreduced gametes, i.e., 2n (somatic chromosome number), were transmitted by *S. officinarum* to its progeny through meiotic restitution (Bremer, 1961; Price, 1963). The transmission of unreduced gametes explains the overrepresentation (approximately 80%) of the *S. officinarum* genome in subsequent generations of varieties.

As a result of these processes, sugarcane has an ‘artificial’ genome of interspecific constitution (polyploid and aneuploid), produced by human intervention, with a complexity that exceeds most other crops (Vieira et al., 2018). Sugarcane cultivars origin is considered redundant (all modern varieties have, at their base, the same hybrid origin), and the genome is complex and highly polyploid, including a variable number of chromosomes (~10 GB, 2n = 12x = 110 - 130). According to pioneering molecular cytogenetic analyses, *S. officinarum* and *S. spontaneum* represent, respectively, 75 to 85% and 15 to 25% of the chromosomes of sugarcane cultivars, the remaining fraction consisting of recombinants chromosomes originated from pairing and recombination of homeologous chromosomes (D’Hont et al., 1996; Cuadrado, 2004; Piperidis et al., 2010; Piperidis and D’Hont, 2020).

Repetitive DNA constitutes a substantial fraction of the plant genome, ranging from ~3% in the small carnivorous plant *Utricularia gibba* (82 Mb; Ibarra-Laclette et al., 2013) to 85% in allohexaploid wheat (*Triticum aestivum*) (Wicker et al., 2018) or maize genomes (Schnable et al., 2009). Also known as the repeatome, repeat motifs vary extensively in composition, abundance and genomic distribution (Macas et al., 2015; Ramakrishnan et al., 2022). Major categories of repetitive elements include ribosomal DNAs (rDNAs) [35S (18S–5.8S-26S) and 5S rDNAs], telomeric repeats, class I retrotransposons - amplified via RNA intermediate, using a ‘copy-and-paste’ mechanism **-**, class II DNA transposons - amplified through DNA copies, using a ‘cut-and-paste’ mechanism -, and tandem repeats (Biscotti et al., 2015). Among these, long terminal repeat (LTR)-type retrotransposons are typically the most frequent transposable element (TE) type in plant genomes, especially for species with large genome sizes, such as sugarcane, wheat, maize, and cotton (Schnable et al., 2009; Appels et al., 2018; Zhang et al., 2018; Yang et al., 2020).

In some polyploids, hybridization leads to rapid proliferation of repeats and genome expansion (McCann et al., 2018). In others, plants do not show a proliferation of different repetitive elements classes, only a gradual and low increase or decrease of preexisting elements in their derived subgenomes (Chen, 2007). Other plant groups have experienced an opposite trend, with high-level polyploids exhibiting a drastic reduction in genome size and a considerable shrinkage of their repeatome (Chen, 2007; Parisod et al., 2010). The mechanisms of removal of the repetitive elements from the genome, attributed to several recombination mechanisms, and the underlying dynamics that equilibrate the expansion and contraction of the repeatome remain poorly understood (Fedoroff, 2012; Drouin et al., 2021). These contrasting responses generate lineage-specific patterns of genome expansion or contraction.

Previous studies have characterized the repeatome of *S. officinarum* and *S. spontaneum* (Huang et al., 2020; Wang et al., 2022), providing key insights into sugarcane genome composition, yet no repeatome analysis has included the full range of the “*Saccharum* complex” and its close relatives in *Miscanthus* and *Erianthus*. Such comparative analyses are essential for an understanding of genome evolution, repeat diversification, the molecular consequences of polyploidy, and the genomic signatures of hybridization and domestication.

Here, we expand the analysis to 30 representatives of the “*Saccharum* complex”, encompassing 9 species – both wild and domesticated – as well as several sugarcane cultivars. Our aim is to elucidate the structure, organization, and evolutionary significance of major repetitive DNA classes, and to investigate the role of the repeatome in shaping genome evolution in *Saccharum*. The characterization of repetitive DNA offers insights into evolutionary and speciation dynamics within the genus, suggesting possible contributions of hybridization and dysploidy events (fission/fusion), particularly in *S. spontaneum*. This enables elucidation of the potential role of repeats in genome size divergence and in the evolution of both genomes and species. The objectives of our study were: (i) to profile and quantify repetitive DNA and identify unique or preponderant repeats; (ii) to test the plausible correlation between genome size and the abundance of the repeats; (iii) to identify repeat types that may have contributed to the expansions or contractions of genomes and their relationships with the ploidy levels, repeat bursts after hybridization events and the phylogenetic positions of the groups; and (iv) to assess the phylogenetic value of repeats using phylogenetic reconstructions and phylogenetic signal approaches.

## 2 Methods

### 2.1 Data acquisition and quality assessment

All the data used in this study is available in the European Nucleotide Archive (ENA) at EMBL-EBI. A total of 27 *Saccharum* genotypes, two *Miscanthus* and one *Erianthus* were evaluated, totaling 30 genotypes (**Supplementary Table 1**).

The quality parameters of the raw Illumina reads in FASTQ format were visualized using FastQC (https://www.bioinformatics.babraham.ac.uk/projects/fastqc/). Sequences were trimmed to 150 bp, low quality bases (phred <30) removed, then converted to FASTA format. The FASTA files were set to the ‘interlaced’ format (reads r1 and r2 combined into one file). These steps were performed using the reformat.sh program (https://sourceforge.net/projects/bbmap/).

The estimation of the number of sequences analyzed for each species was based on the formula: genome coverage = sample size (bp)/genome size (bp). Genome sizes were previously estimated for some *Saccharum* species (Garsmeur et al., 2025) (**Supplementary Table 2**). A sample of 5 million reads was used for all analyzed genotypes, except for *E. fulvus*, for which 1 million reads were used due to its small genome size, in order to obtain the desired coverage, which ranged from 0.09× to 0.33×. The sampling of the dataset was carried out in reformat.sh (https://sourceforge.net/projects/bbmap/).

### 2.2 Clustering and annotation of repetitive DNA

Repetitive sequences were identified by similarity-based clustering of Illumina paired-end reads using RepeatExplorer2 (Novák et al., 2020). Three different analyses were performed: (*i*) an individual analysis to characterize all types of repeats in each genotype, (*ii*) a comparative analysis with all genotypes, and (*iii*) a comparative analysis within each species and of hybrids.

As the reads in the comparative clustering are analyzed together, each species was renamed with a specific code. The tool ‘RepeatExplorer Utilities/FASTA read name affixer’ was used to add genotype-specific seven-letter prefixes to the read names. The ‘Text manipulation/Concatenate datasets’ tool was then used to join the FASTA files.

Default parameters were employed for all analyses. Repeats were automatically annotated based on REXdb (release Viridiplantae v4.0), the most complete, up-to-date database of domains of LTR-RT evolutionary lineages, available in RepeatExplorer2 (Neumann et al., 2019).

All clusters representing at least 0.01% of the genome were manually inspected to correct possible annotation errors (Novák et al., 2020). RepeatExplorer2 output files with the description of the main results were recovered for each species and used to check annotations. Superclusters with discordant annotations, resulting from conflicting evidence and pointing to different types of repetitive elements, were split into groups of clusters, annotated as single repetitive elements. Clusters with no evidence were annotated as ‘Unknown_repeats’.

Clusters were used to characterize and quantify the repeats. Genomic proportions of the repeat types were calculated based on the proportion of reads in individual annotated clusters. The Revis-master tool, available in RepeatExplorer2, was used for graphic visualization of the shared lineages in the comparative analysis. For a better visualization of the shared lineages ‘Unknown_repeats’ annotated clusters were removed from these graphs.

Statistical analyses were performed in R version 4.3.2 (R: The R Project for Statistical Computing, n.d.) using the packages *ggplot2* and *car*. The dataset included genome size (Mbp), chromosome number, ploidy level, and proportion of repetitive DNA (%). Absolute repetitive DNA content (Mbp) was calculated as genome size × (% repetitive DNA/100), using genome size estimates provided in **Supplementary Table 2**. Pearson’s correlations were used to assess relationships between repetitive DNA and genomic parameters. Multiple linear regression models were fitted to test the combined effects of genome size and (i) chromosome number or (ii) ploidy. Model performance was evaluated using adjusted R², variance inflation factors (VIF), and Akaike Information Criterion (AIC). Results were visualized with scatterplots and regression fits, and residual and observed–predicted analyses were undertaken to verify model adequacy.

### 2.3 Satellite DNA analyses

Satellite DNA (satDNA) clusters were identified by TAREAN (Tandem Repeat Analyzer), included in RepeatExplorer2 (Novák et al., 2013, 2020). These clusters were further manually examined. The consensus_dimer.fasta file, comprising reconstructed consensus dimers generated by the TAREAN, were retrieved, aligned with Geneious Prime v. 2022.2 (https://www.geneious.com) and their structure examined with the dotplot tool.

For final characterization and annotation, the homology of all satDNAs was checked. Monomer sequences were aligned in Geneious Prime v.2022.2 (https://www.geneious.com). satDNA classification followed the criteria established by Ruiz-Ruano et al. (2016), utilizing specific sequence similarity thresholds: 1. Monomeric sequences displaying less than 80% similarity were assigned to different families within the same superfamily; 2. Sequences sharing 80–95% similarity were considered variants belonging to the same family; and 3. Sequences exhibiting greater than 95% similarity were classified as variants of the same consensus monomer. The satDNA families were designated using a four-letter species prefix (e.g., Soff for *S. officinarum*), followed by the descriptor ‘Sat’, a numerical identifier assigned by decreasing abundance, and the consensus monomer length.

Finally, the identified satDNA sequences were characterized in terms of genome proportion, monomer length and AT content. The genomic abundance of each satDNA was quantified by TAREAN, based on the relative frequency of reads assigned to each cluster.

All identified satDNA were compiled in a presence-absence matrix, and pairwise Jaccard similarity coefficients (Jaccard, 1908) were calculated to quantify shared satDNA content. Heatmap representations of the similarity matrix were generated using package pheatmap (Kolde, 2019) and UpSet plot representing the shared SatDNA among genotypes was generated using the package ggplot2 (Wickham, 2016) in R v. 4.3.2 (R: The R Project for Statistical Computing, n.d.).

### 2.4 Comparison of the LTR-RT fraction in *S. officinarum, S. spontaneum* and modern cultivar R570 fully assembled genomes

To compare the LTR fraction of available complete genomes, we used the DANTE tool (Domain-based ANnotation of Transposable Elements - Neumann et al., 2019; Novák et al., 2024) and analysed the genomes of *S. officinarum* (LA-Purple, PRJNA744175, unpublished, 1.3 Gb), *S. spontaneum* (AP85-441, 3.1 Gb - Zhang et al., 2018), and modern cultivar R570 (5.04 Gb - Healey et al., 2024).

### 2.5 Phylogenetic analyses of repeats

Phylogenetic analysis based on repeat sequence similarities followed the methodology described by Vitales et al. (2020) with modifications. For each of the 100 most abundant repeat clusters identified by RepeatExplorer2 during the total comparison, the observed/expected (O/E) number of matrix edges was extracted. This matrix represents pairwise sequence similarity values between species for each repeat family, normalized by the number of reads contributed by each species to the cluster (Vitales et al., 2020). The O/E similarity matrices were converted to distance matrices by calculating the inverse of similarity values (distance = 1/similarity). For each of the top 100 repeat clusters, a neighbor-joining (NJ) tree was constructed using the NJ function from the ape package in R (Paradis and Schliep, 2019). Clusters lacking pairwise similarity values for all species pairs were excluded from further analysis.

All individual NJ trees were summarized in a consensus network using the consensusNet function from the phangorn package in R (Schliep, 2011). The consensus network was constructed with a threshold of 20%, meaning splits present in at least 20% of the individual trees were included in the final network. The resulting network was visualized and exported using FigTree v.1.4.4.

## 3 Results

### 3.1 Global composition of the *Saccharum* repeatome

**Figure 1:**
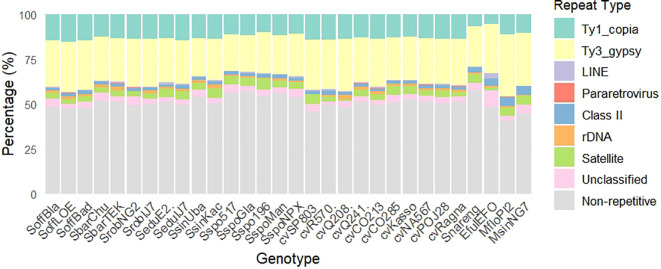
Relative abundance (%) of repetitive sequences in 30 genotypes of the “*Saccharum* complex” identified using RepeatExplorer2.

Low-coverage genome sequencing followed by RepeatExplorer2 analysis characterized the repetitive DNA landscape across 30 genotypes representing the “*Saccharum complex”*, including *S. officinarum, S. barberi, S. robustum, S. edule, S. sinense, S. spontaneum, S. narenga*, modern cultivars, *Miscanthus sinensis, Miscanthus floridulus* and *Erianthus fulvus* (**Supplementary Table 1**). Genome coverage for individual analysis ranged from 0.1x to 0.33x (**Supplementary Table 2**). Total repetitive DNA content varied substantially among taxa, from 42.5% in *S. narenga* to 59.7% in *M. floridulus* (**Table 1**, **Figure 1**). Long terminal repeat retrotransposons (LTR-RTs) represented the predominant component of the repetitive genome, although their relative abundance differed among genotypes.

**Table 1:** Genomic proportion (%) of major repeat identified in individual analyses of the “*Saccharum* complex” genotypes using RepeatExplorer2.

Genotype	LTR Ty1			LTR Ty3				Non-LTR		DNA transposon		Tandem repeats		Unclassified	Total
	SIRE	Ale	Other	Tekay	Retand	Athila	Other	LINE	Pararetrovirus	EnSpm_CACTA	Other	rDNA	satDNA		
SoffBla	12.13	0.95	1.68	18.10	3.83	2.62	1.31	0.47	0.07	0.92	0.57	1.01	3.29	4.61	51.56
SoffBad	11.94	1.07	1.66	18.82	3.99	2.64	1.93	0.58	0.07	1.01	0.99	1.23	2.84	3.48	52.25
SoffLOE	12.33	1.28	1.92	18.82	4.00	2.84	2.15	0.61	0.06	1.00	1.03	1.64	2.35	2.49	52.52
SrobNG2	11.43	1.04	1.63	18.38	3.61	2.56	1.90	0.47	0.07	0.97	0.73	0.23	2.81	4.89	50.72
SrobIJ7	11.07	1.17	1.65	17.70	3.62	2.61	2.37	0.56	0.04	1.06	0.78	1.53	2.82	2.98	49.96
SeduE2.	11.06	0.96	1.52	16.52	3.81	1.83	2.39	1.06	–	0.85	0.62	0.95	4.67	2.37	48.61
SeduIJ7	11.39	0.94	2.08	16.77	3.84	1.90	1.91	0.79	–	1.64	0.50	1.04	4.66	3.14	50.60
SbarChu	10.06	1.20	1.52	15.39	3.62	3.48	1.95	0.40	0.02	0.81	0.76	1.56	3.20	4.57	48.54
SbarTEK	10.61	1.14	1.45	15.24	3.67	3.35	2.17	0.86	0.06	0.92	0.91	1.99	3.54	2.78	48.69
SsinUba	10.66	0.94	1.58	13.81	3.11	3.09	1.52	0.39	–	0.92	0.53	1.77	3.70	3.94	45.96
SsinKac	11.13	1.21	1.45	14.44	3.44	2.93	2.30	0.66	–	0.77	0.71	2.16	5.26	3.11	49.57
Sspo517	9.11	1.35	0.89	10.29	3.97	3.66	2.35	0.30	0.03	0.97	0.75	1.00	4.66	4.52	43.85
SspoGla	9.14	1.40	1.29	10.13	3.50	4.00	2.46	1.11	0.04	0.93	0.75	0.73	4.62	2.82	42.92
Sspo196	7.76	0.96	1.53	10.60	2.76	5.73	3.70	0.36	0.06	0.85	1.38	0.28	6.35	3.28	45.60
SspoMan	8.35	1.71	1.71	10.34	4.13	4.59	2.53	0.48	–	1.07	0.99	1.1	3.89	2.69	43.58
SspoNp-X	7.88	1.23	1.77	12.10	3.19	5.41	3.14	0.76	0.06	0.75	0.69	0.75	3.73	4.62	46.08
cvSP803	11.88	0.75	1.52	15.45	2.02	6.79	3.66	0.75	0.08	1.32	0.52	0.03	5.16	4.04	53.96
cvR570.	11.90	0.72	1.70	18.15	3.28	3.60	2.22	1.03	0.10	1.33	1.07	0.88	2.63	1.76	50.37
cvQ208.	9.67	2.00	2.41	17.38	4.51	2.83	4.31	0.38	0.05	1.02	0.66	2.38	0.70	3.42	51.71
cvQ241.	10.09	1.04	1.97	16.57	3.50	2.96	1.91	0.51	0.05	0.91	1.01	1.42	3.98	2.90	48.82
cvCO213	11.09	1.22	1.64	17.24	3.72	3.41	2.18	0.98	0.05	0.96	0.67	1.72	3.08	2.46	50.42
cvCO285	10.56	1.24	1.39	14.11	3.99	3.09	2.26	0.55	–	0.84	0.57	1.60	4.76	4.01	48.97
cvKasso	10.13	1.02	1.48	15.11	3.68	3.08	2.08	0.45	0.06	0.87	0.72	1.41	4.30	3.08	47.47
cvNA567	10.79	1.03	1.60	16.48	3.61	3.11	2.39	0.47	0.06	0.98	0.79	1.36	2.69	3.46	48.82
cvPOJ28	11.25	0.98	1.61	17.04	3.68	2.61	1.73	0.90	0.09	0.82	0.61	1.17	3.91	3.05	49.45
cvRagna	11.17	0.99	1.58	17.33	3.33	3.03	2.29	0.52	0.06	1.02	0.97	1.03	2.72	2.61	48.65
Snareng	4.80	0.12	2.00	8.72	6.18	5.65	1.81	0.51	0.02	1.96	0.68	0.86	5.00	4.18	42.50
EfulEFO	4.73	0.23	1.19	11.83	7.87	6.50	1.45	–	–	1.69	0.48	0.88	2.63	4.96	44.44
MfloPI2	8.68	0.19	2.45	14.37	12.30	1.64	6.17	0.33	0.09	4.45	0.70	0.67	4.46	3.23	59.74
MsinNG7	7.52	0.55	2.36	11.27	13.16	0.92	4.11	0.28	–	4.31	0.41	0.58	4.82	5.17	55.46

**Figure 2:**
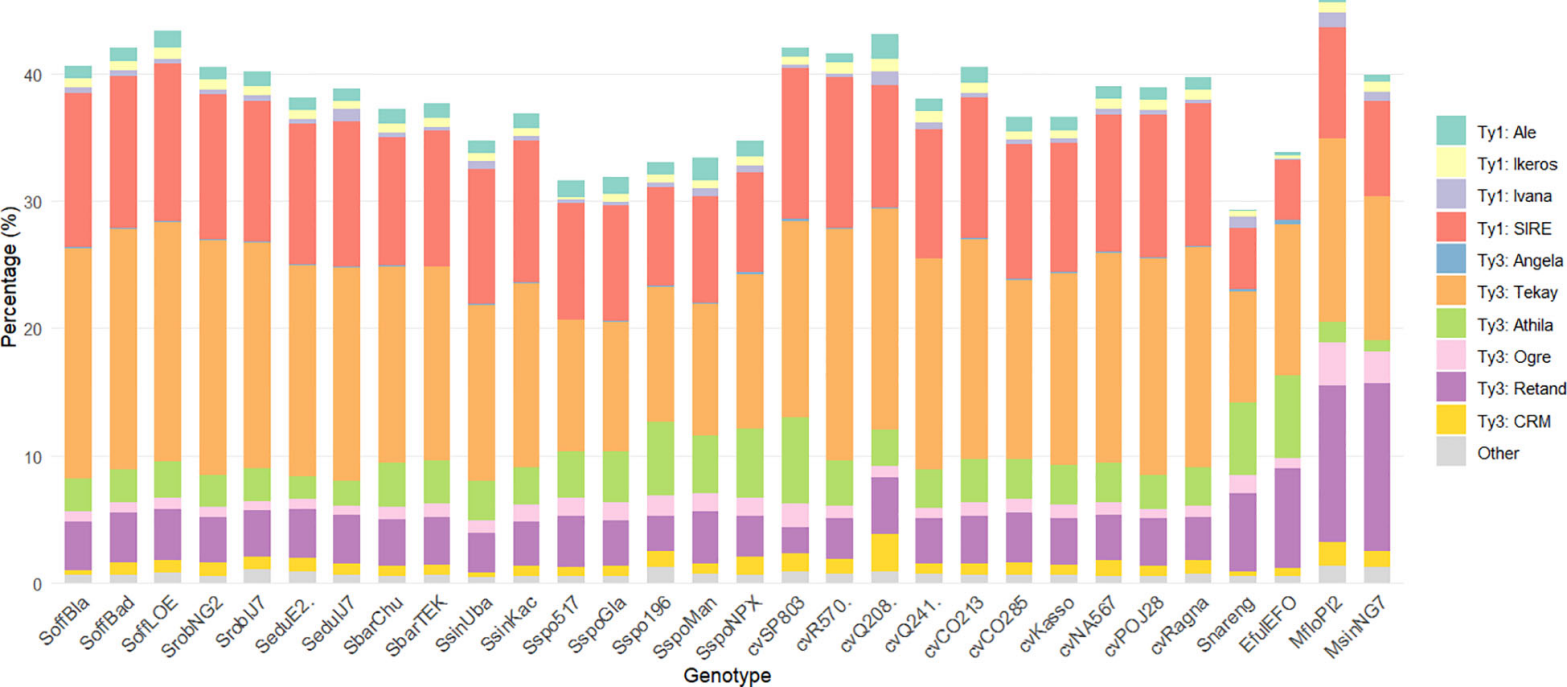
Genomic proportion of LTR-RTs identified in the *“Saccharum* complex” using RepeatExplorer2.

Within the Ty1 LTR-RTs superfamily, SIRE lineage was the most abundant, whereas among Ty3 LTR-RTs elements, the Tekay clade was predominant. The genomic proportion of Ty1 LTR-RTs elements ranged from 6.15% in *E. fulvus* to 15.53% in SoffLOE, while Ty3 LTR-RTs elements accounted for 20.1% in *S. spontaneum* Glagah up to 34.5% in MfloPI2. Retand and Athila were the next most abundant in Ty3 LTR-RTs (**Figure 2**; **Supplementary Table 3**). Genotype-specific elements were detected at low frequency. The chromovirus Tcn1 was detected in cvSP803 (0.30%) and in *S. edule* IJ76-337 (0.02%), whereas the non-chromoviruses TatII and Tatius were restricted to *S. edule* E2 (0.45%) and cultivar R570 (0.01%), respectively.

Non-LTR retrotransposons (mainly LINEs), DNA transposons (EnSpm/CACTA, Tc1/Mariner, PIF/Harbinger), and tandem repeats together represented minor fractions but showed genotype-specific variation.

Regarding tandem repeats, 35S rDNA ranged from 0.03% in cvSP803 to 2.27% in cvQ208, and 0% to 0.30% in cvSP803 and SoffLOE, respectively for 5S. SatDNA sequences were present in all genotypes, ranging from 0.70% in cultivar Q208 to 6.35% in *S. spontaneum* SES196 (**Supplementary Table 3**).

### 3.2 Drivers of repeatome variation

**Figure 3:**
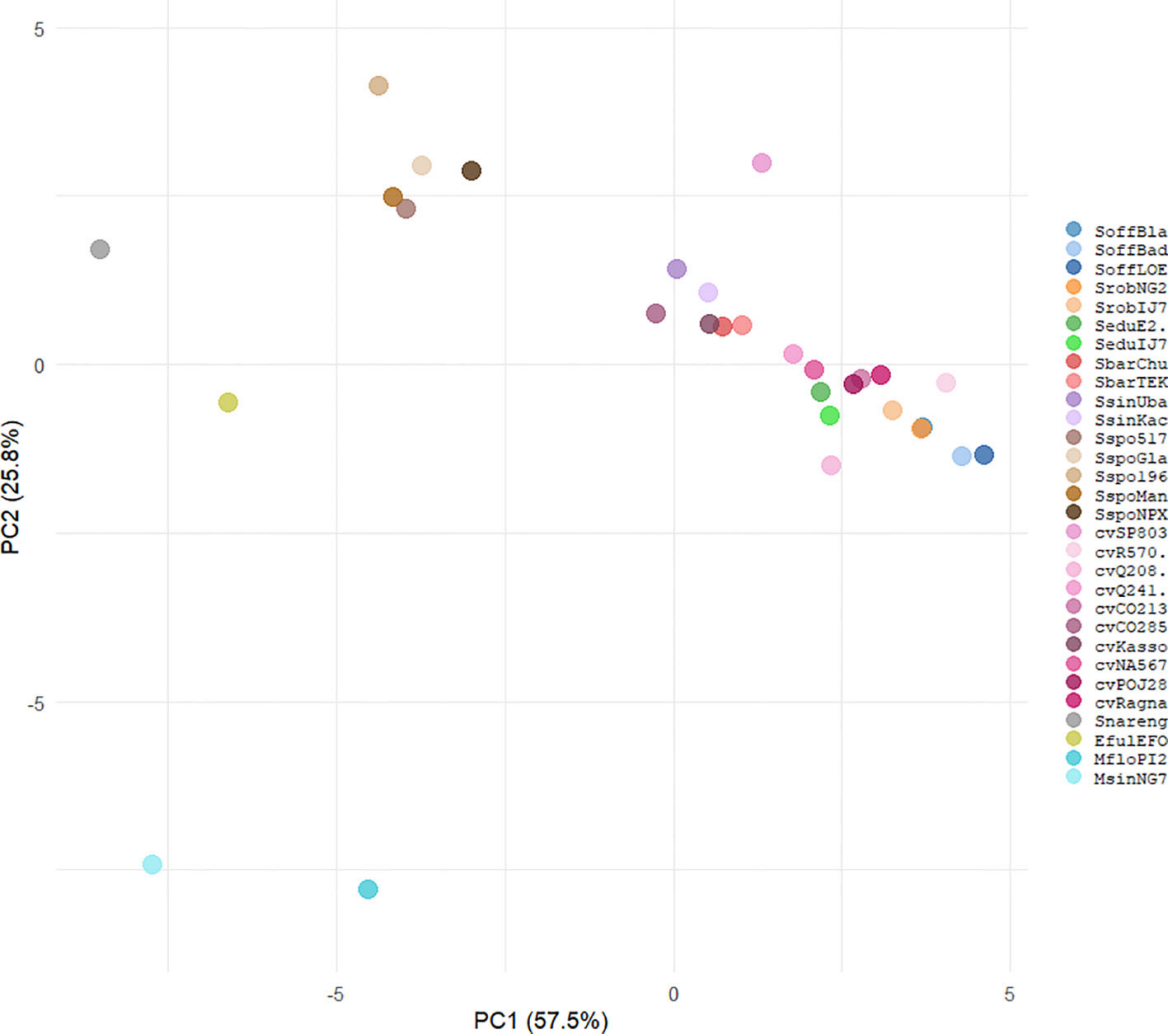
Principal Component Analysis (PCA) of the repetitive element classes across 30 “*Saccharum* complex” genotypes.

Principal Component Analysis (PCA) based on the relative abundances of major repeat classes indicated clear patterns of variation among genotypes (**Figure 3**). The first two principal components (PC1 and PC2) collectively explained 83.3% of the total variance (PC1: 57.5%, PC2: 25.8%). The lineages Retand, Tekay, SIRE and Athila were the main contributors to this variation (**Supplementary Figure 1**).

Species with large proportions of Tekay and Retand (e.g., *Miscanthus* species) clustered at one end of PC1, whereas *S. narenga, E. fulvus* and several *S. spontaneum* genotypes, which exhibited lower Ty3 LTR-RTs content and higher heterogeneity, were distributed across distinct regions of the PCA space. Cultivars formed a cohesive cluster intermediate between *S. officinarum* and *S. spontaneum*, consistent with their hybrid origin. *S. robustum, S. edule*, *S. barberi* and *S. sinense* are closer to *S. officinarum* than to *S. spontaneum*.

**Figure 4:**
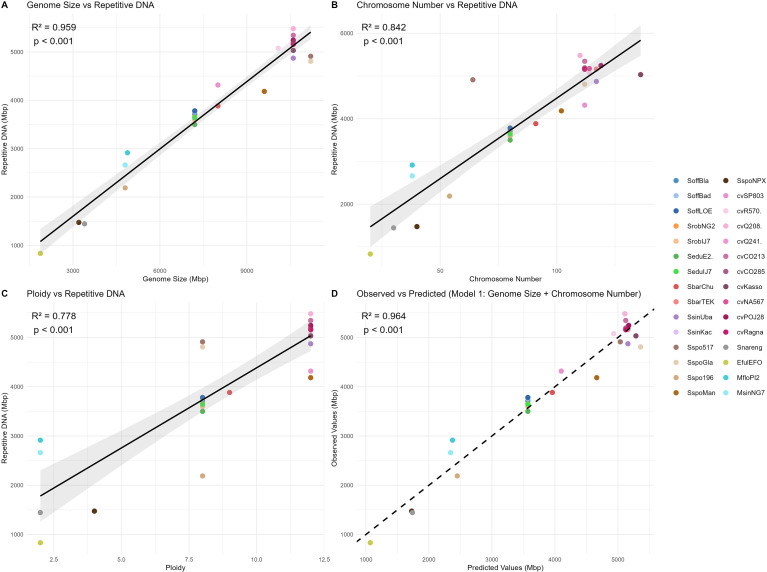
Correlation between the amount of repetitive DNA and **(A)** genome size, **(B)** chromosome number, **(C)** ploidy and **(D)** multiple regression analyses in *Saccharum*.

There is a strong positive association between genome size and the amount of repetitive DNA across the evaluated genotypes, with an extremely high correlation (R^2^ = 0.96; p < 0.001), indicating that larger genomes contain proportionally more repetitive content (**Figure 4A**). Chromosome number and ploidy also showed significant correlations with repetitive DNA (R^2^ = 0.84 and R^2^ = 0.77, respectively; p < 0.001) (**Figures 4B, C**). Multiple regression analysis indicated that genome size alone explains most of the variation in repetitive DNA content (R² ≈ 0.96) (**Figure 4D**). Neither chromosome number nor ploidy level remained statistically significant when genome size was included in the model, with p values of p = 0.072 and p = 0.17, respectively. Both regression models explained a high proportion of the variance (R² ≈ 0.96), indicating that variation in genome size is the main driver of repetitive DNA abundance across the analyzed taxa.

### 3.3 Satellite DNA diversity and distribution

Across all genotypes, 220 clusters annotated as satDNAs were identified using TAREAN. Ninety-one of these were high confidence satDNAs, expected to form long, homogeneous arrays of highly conserved monomers (**Supplementary Table 4**, Supplementary File 1). Additional putative satDNAs were identified and analysed based on their repeat pattern in heatmap analyses (Supplementary File 1).

SatDNA were identified in all *Saccharum* genotypes. The number of satDNA families per genome varied widely. SsinUba, cv. SP80–3280 and cv. Q208 exhibited 5 satDNA, while *M. floridulus* PI295762 exhibited 15 satDNAs. The genomic abundance of individual satDNA clusters ranged from 0.01% to 3.4%, with total satDNA proportion ranging from 0.70% to 6.35%, in cv. Q208 and *S. spontaneum* SES196, respectively. Monomer lengths ranged from 7 to 10,819 bp (**Supplementary Table 4**).

**Figure 5:**
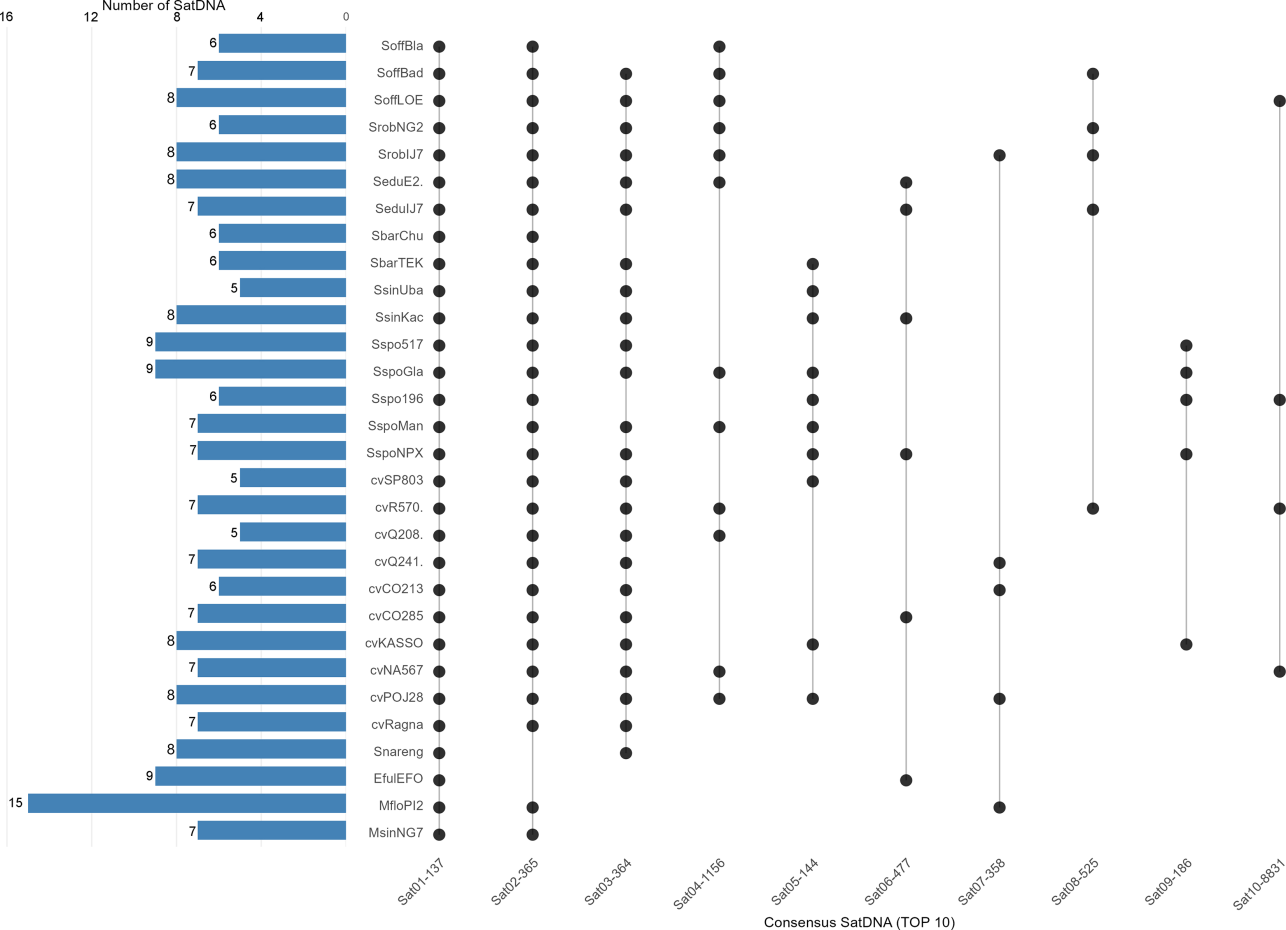
Conservation of satellite DNA families across the *Saccharum* complex.

Homology-based analyses identified 16 variants of the same monomer (>95% of homology), 5 variants of the same family (80-95% of homology) and 14 different families within the same superfamily (<80%), totalling 22 families. The most shared satDNA was Sat01-137, shared with all genotypes, followed by Sat02–365 shared with 28 genotypes (**Figure 5**).

The 137 bp monomer satDNA is common to all analyzed genotypes (Supplementary File 1). The genomic abundance of this satDNA ranged from 0.71% in *M. floridulus* to 3.4% in cultivar SP80-3280. Sequence comparison using BLAST corroborated the homology of these clusters to the previously described sugarcane centromeric repeat, *CENT2* (Vieira et al., 2018; Huang et al., 2021). This indicates strong centromeric conservation across the “*Saccharum* complex”.

### 3.4 Comparative repeat profiling across genotypes

**Figure 6:**
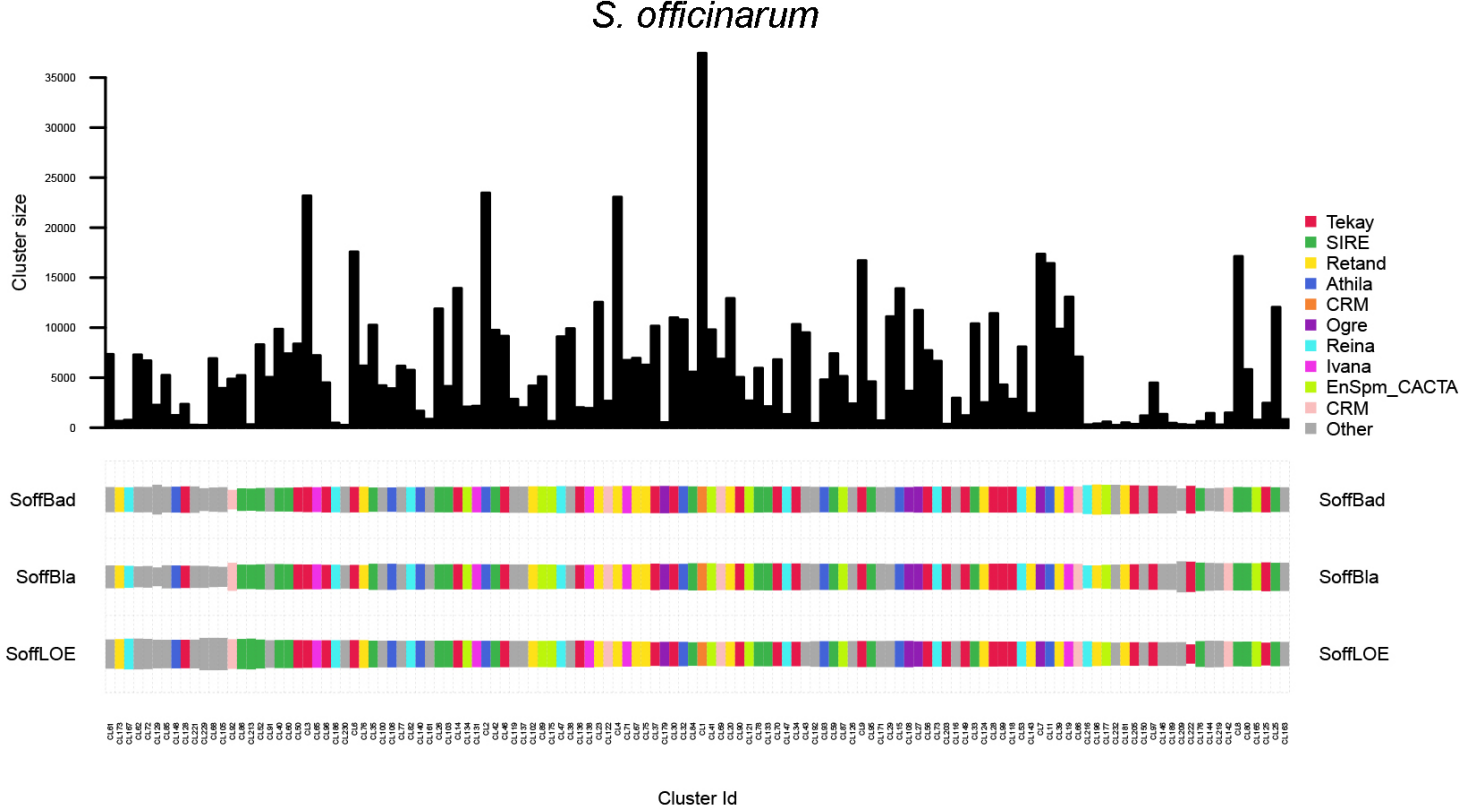
Comparison of repeat composition in *S. officinarum* genotypes inferred from a comparative RepeatExplorer2 analysis. The columns represent different clusters, the colors different repeat classes, and the size of the rectangles is proportional to the number of reads in a cluster for each species. ‘Other’ includes repeat types not among the most abundant categories, such as minor LTR-RT lineages and low-abundance DNA transposons below the display threshold.

The clustering pattern demonstrated that *S. officinarum* genotypes share nearly identical repeat profiles, with strong dominance of Tekay and SIRE and similar number of reads per cluster (**Figure 6**).

**Figure 7:**
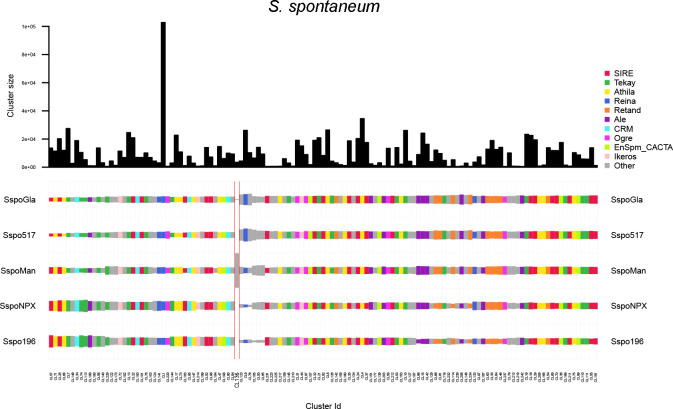
Comparison of repeat composition in *S. spontaneum* genotypes inferred from a comparative RepeatExplorer2 analysis. The columns represent different clusters, the colors different repeat classes, and the size of the rectangles is proportional to the number of reads in a cluster for each species. ‘Other’ includes repeat types not among the most abundant categories, such as minor LTR-RT lineages and low-abundance DNA transposons below the display threshold.

*S. spontaneum* genotypes exhibited intra-species diversity, primarily driven by variation in Athila, Retand, and low-abundance clusters. Notably, CL136 (5S rDNA) absent in *S. spontaneum* SES196 and *S. spontaneum* NP-X, and CL228 (Unknown) is present only in *S. spontaneum* Glagah and *S. spontaneum* SES 517 (**Figure 7**).

**Figure 8:**
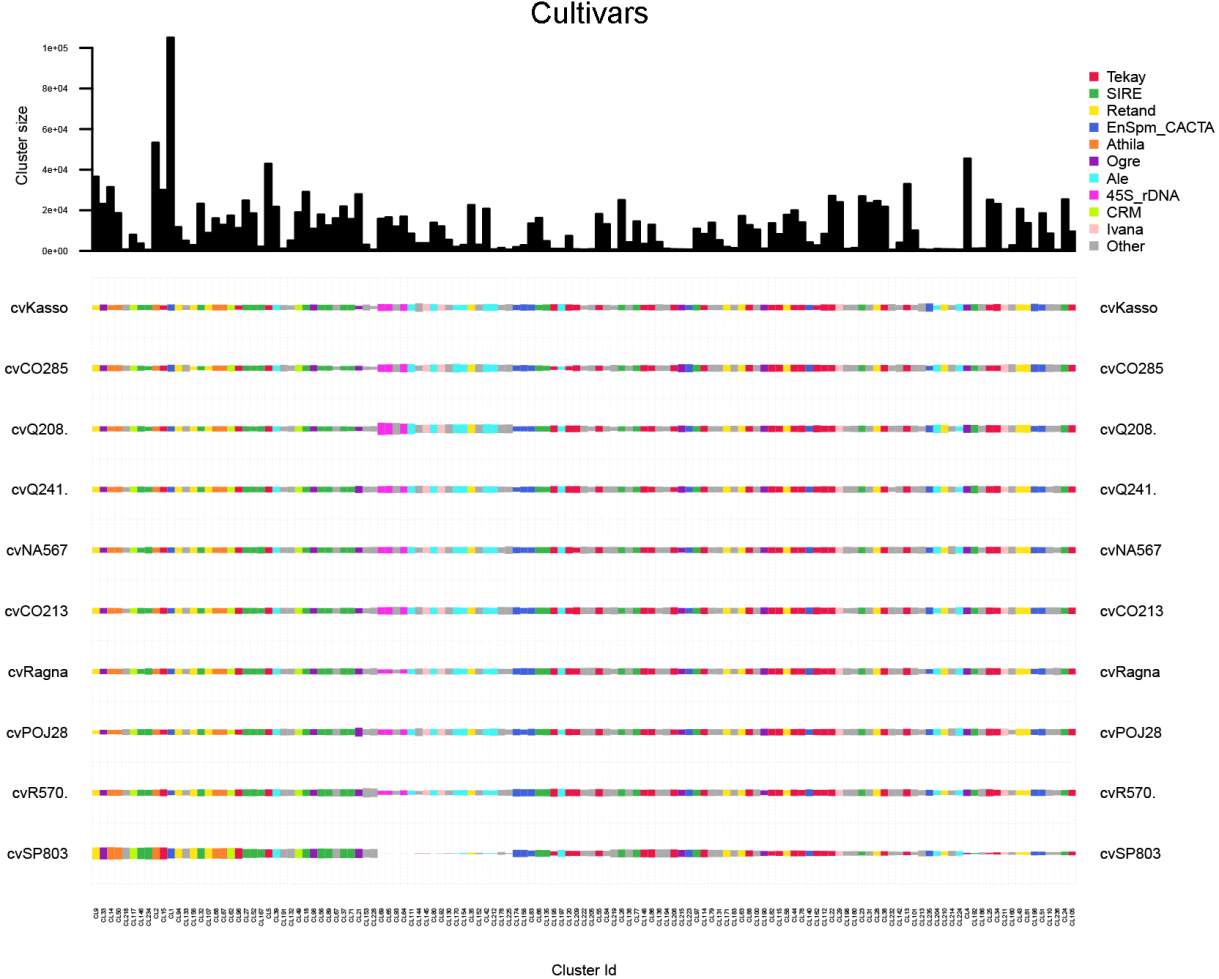
Comparison of repeat composition in cultivars inferred from a comparative RepeatExplorer2 analysis. The columns represent different clusters, the colors different repeat classes, and the size of the rectangles is proportional to the number of reads in a cluster for each species. ‘Other’ includes repeat types not among the most abundant categories, such as minor LTR-RT lineages and low-abundance DNA transposons below the display threshold.

Cultivars derived from interspecific hybridization displayed intermediate repeat profiles, retaining repeat families from both parental species, *S. officinarum* and *S. spontaneum*. The most shared clusters belong to Tekay and SIRE. A few rare clusters showed restricted distribution among cultivars: CL189 (Unknown) is present only in cvCO213 and cvNA567; CL155 (Unknown) occurred in cvQ241, cvRagna and cvQ208; CL116 (Unknown) is absent in cvSP803; and CL173 (Unknown) is present only in cvPOJ28 (**Figure 8**).

**Figure 9:**
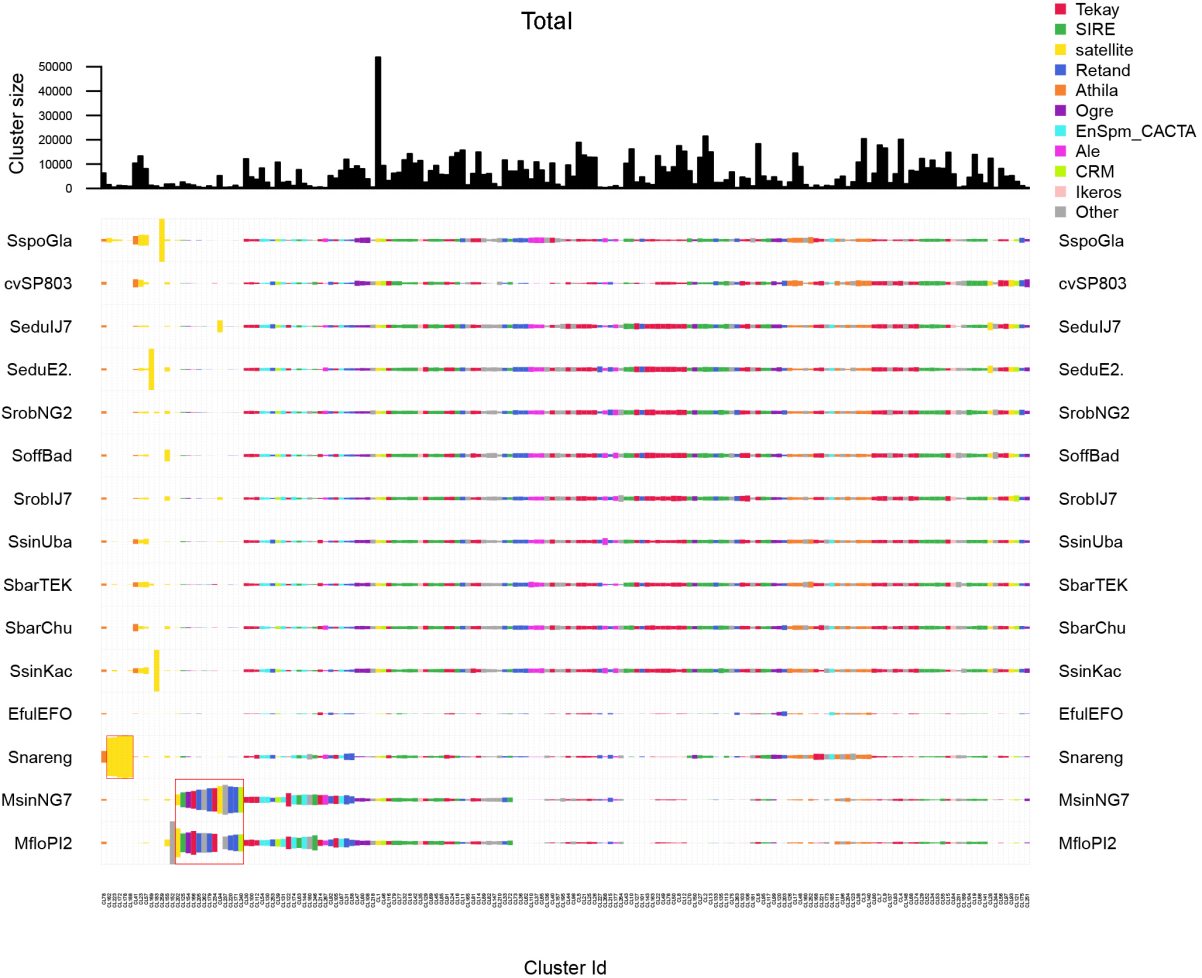
Comparison of repeat composition in all *Saccharum* genotypes inferred from a comparative RepeatExplorer2 analysis. The columns represent different clusters, the colors different repeat classes, and the size of the rectangles is proportional to the number of reads in a cluster for each species. ‘Other’ includes repeat types not among the most abundant categories, such as minor LTR-RT lineages and low-abundance DNA transposons below the display threshold. Only one representative genotype of *S. officinarum*, *S. spontaneum* and cultivars is shown for clarity, as profiles within groups were highly similar.

A comparative analysis of all genotypes reveals a similar pattern for most clusters. The most shared clusters belong to Tekay, SIRE and Athila (**Figure 9**). The *Miscanthus* species (MsinNG7 and MfloPl2) exhibited a more specific repeat pattern, with several SIRE (CL125), Retand (CL205, CL179, CL220, CL171), Ogre (CL154), Tekay (CL166, CL234), CRM (CL240) and SatDNA (CL94, CL202) clusters. *S. narenga* contained several exclusive clusters, CL162, CL223, CL172, CL178 and CL188, all annotated as SatDNA, suggesting independent repeat diversification (**Figure 9**).

### 3.5 Repeatome-based phylogenetic relationships

**Figure 10:**
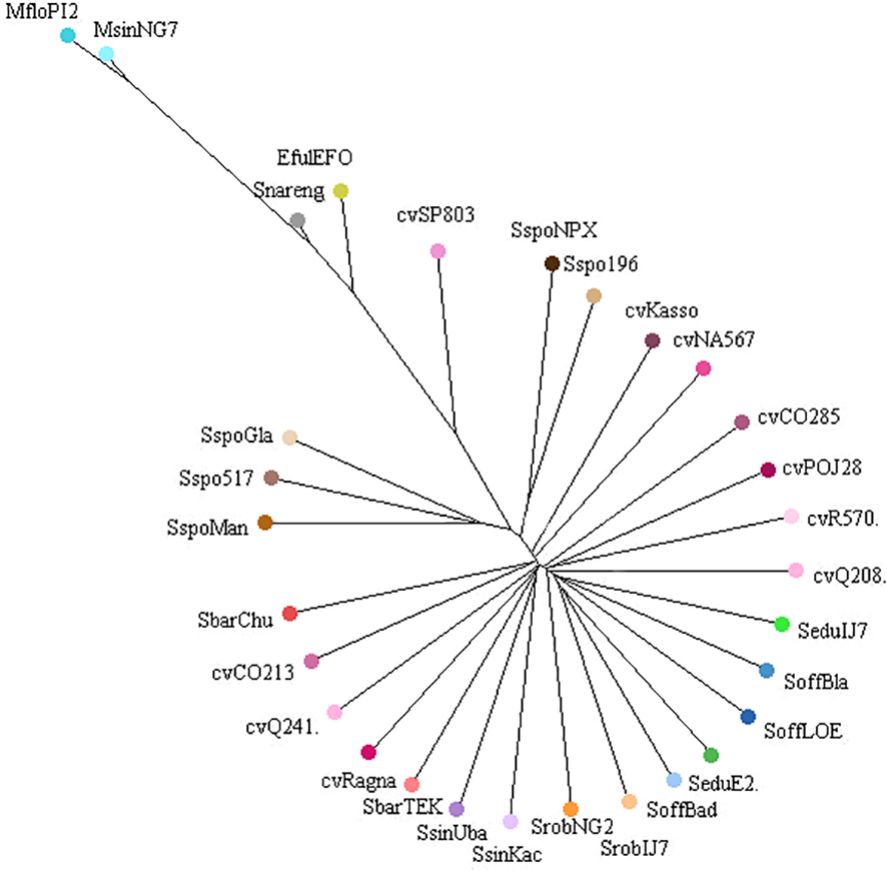
Neighbor-Joining consensus tree based on the similarity of repetitive DNA profiles. Different colors correspond to genotypes from the same species or cultivars.

The 100 most abundant clusters were used to generate an NJ consensus tree, which clearly delineated the major groups within the *Saccharum* complex (**Figure 10**). The tree showed a large central clade predominantly composed of cultivars, *S. officinarum, S. barberi, S. robustum*, *S. sinense* and *S. edule*, characterized by highly similar repetitive DNA profiles, reflecting their close genomic relationships. *S. spontaneum* genotypes formed two distinct branches: (i) *S. spontaneum* NP-X and *S. spontaneum* SES196, (ii) *S. spontaneum* Glagah, *S. spontaneum* SES517 and *S. spontaneum* Mandalay. Intermediate positioning of *S. narenga* and *E. fulvus* indicates partial sharing of repeat families with the main group, whereas *M. sinensis* and *M. floridulus* formed a distinct, long-branched cluster, suggesting a markedly divergent repetitive DNA composition (**Figure 10**).

### 3.6 Comparison of the LTR-RT fraction in *Saccharum* assembled genomes

**Figure 11:**
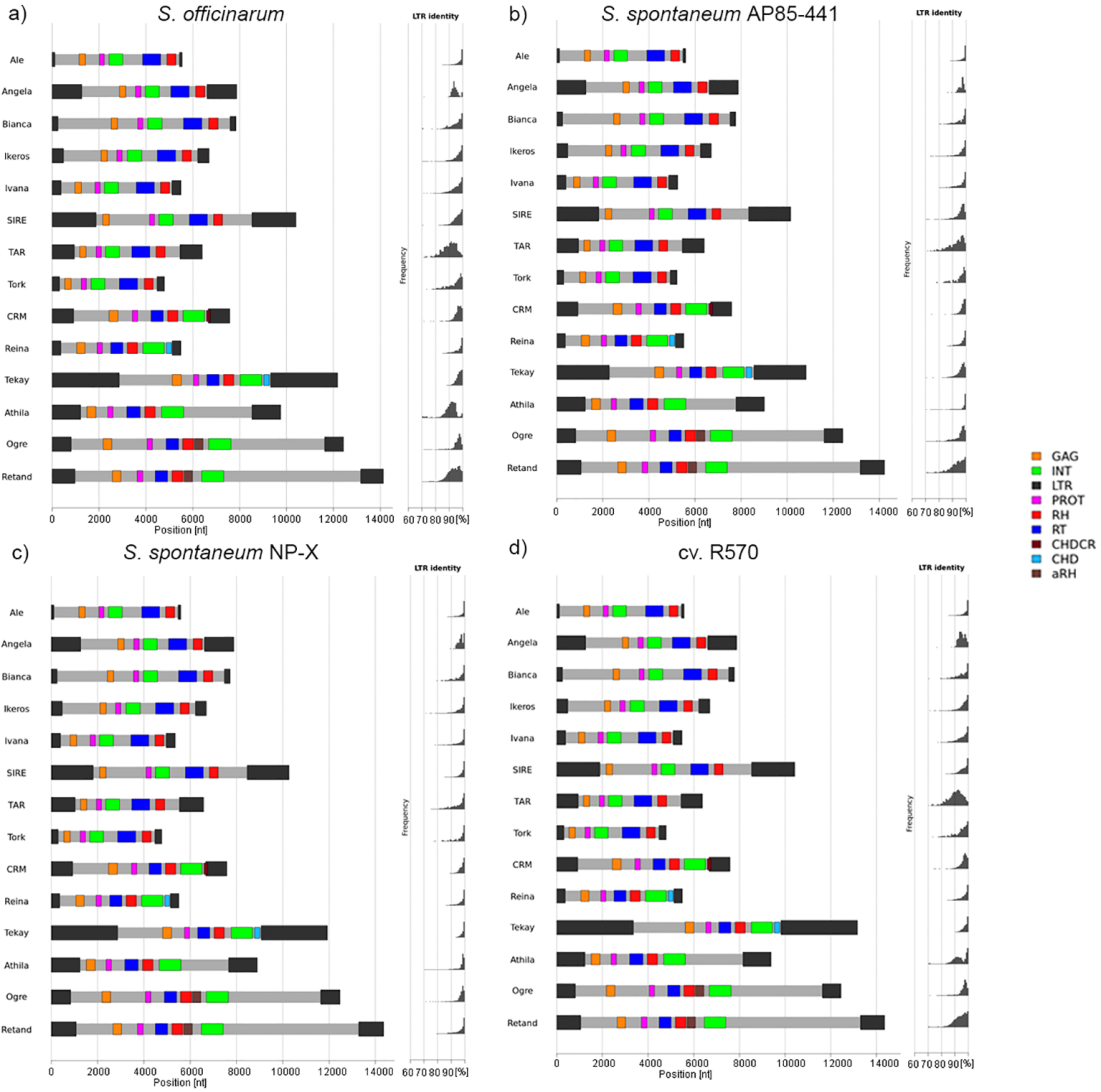
Structural annotation and LTR identity distribution of LTR retrotransposons in *S. officinarum* LA-Purple **(a)**, *S. spontaneum* AP85-441 **(b)**, *S. spontaneum* Np-X **(c)** and cultivar R570 **(d)**. The horizontal axis represents the nucleotide position along the consensus sequence (bp), while colored blocks indicate the main retrotransposon domains (LTR, GAG, PROT, INT, RT, RH, CHD). To the right of each panel, histograms depict the distribution of LTR identity values (%), providing an estimate of relative insertion ages.

DANTE-based annotation of complete genomes of *S. officinarum* LA-Purple*, S. spontaneum* AP85-441*, S. spontaneum* NP-X and cultivar R570 identified distinct evolutionary patterns among species (**Figure 11**, **Supplementary Table 6**, **Supplementary File 2**). In *S. officinarum* (**Figure 11A**), SIRE elements exhibited high sequence coverage across their entire length, with well-preserved domain architecture. LTR identity peaks were close to 100%, indicating recent amplification events. Tekay elements were abundant and highly conserved, showing high LTR identity values. Athila and TAR exhibited lower LTR identities (80-90%), consistent with older insertions.

In *S. spontaneum* AP85-441 (**Figure 11B**), SIRE elements remained abundant and highly conserved, with a similar domain structure and high LTR identity peak, as in *S. officinarum*, supporting ongoing or recent insertions. Ale elements, although less abundant, also presented well-preserved structures and a predominance of high LTR identity values, indicating recent insertions. In *S. spontaneum* NP-X (**Figure 11C**), Tekay elements were the most abundant, with a high LTR identity, suggesting very recent insertions. SIRE elements were present at lower amounts but still exhibit a high LTR identity peak.

In cultivar R570 (**Figure 11D**), SIRE elements remained abundant and highly conserved, with a similar domain structure and high LTR identity peak, as in *S. officinarum* and *S. spontaneum.* Tekay elements were the second most abundant and were highly conserved with a high LTR identity. Athila exhibited bimodal LTR identity distributions, likely reflecting insertions from both *S. officinarum* and *S. spontaneum* ancestry, whereas TAR elements appeared to represent older, less active insertions.

## 4 Discussion

The repetitive fraction of plant genomes is highly dynamic, with retrotransposons undergoing rapid evolution that drives extensive sequence divergence and lineage-specific distributions across related species (Wicker et al., 2018). Consequently, species-specific repetitive elements frequently arise. In *Saccharum* the analysis of repetitive DNA was previously performed by Wang et al. (2022), who analyzed repeats from *S. officinarum* and *S. spontaneum* using RepeatExplorer. The total repeat content was 44.9% and 42.4% for *S. officinarum* LA Purple and *S. spontaneum* SES208. In both species, Ty3 LTR-RTs were the most enriched, representing 22.7% in *S. officinarum* and 22.1% in *S. spontaneum*, followed by Ty1 LTR-RTs, accounting for 16.0% and 12.6%, respectively.

Our analysis across a broader panel of accessions corroborates these patterns. Total repetitive DNA ranged from 51.56 to 52.52% in *S. officinarum* and from 42.92 to 46.08% in *S. spontaneum* genotypes (as shown in **Table 1**). Ty3 LTR-RTs elements were the dominant TE lineage, 25.9 - 27.8% in *S. officinarum* and 20.1 - 23.4% in *S. spontaneum*, followed by Ty1 LTR-RTs, 14.7 - 15.5% in *S. officinarum* and 10.3 - 18.8% in *S. spontaneum*. The absence of detectable clusters of 5S rDNA in cvSP803, should be interpreted with caution. RepeatExplorer2 analyses rely on similarity-based clustering of subsampled genomic reads and primarily recover repeats that exceed a minimal abundance threshold in the analyzed dataset. Consequently, repeats with lower genomic representation or uneven sampling may fail to form distinct clusters, even if present in the genome. Similar limitations have been reported in RepeatExplorer-based repeatome analyses (Novák et al., 2020).

It is important to note that repeat abundances estimated from low-coverage sequencing data should be interpreted as approximations rather than exact genomic proportions, as subsampling effects may influence the representation of specific repeat families. However, graph-based clustering approaches such as RepeatExplorer2 have been shown to provide robust and reproducible insights into repeatome composition, particularly for moderately to highly abundant elements (Novák et al., 2020; Macas et al., 2015). In this study, the use of a standardized number of reads across all genotypes minimizes comparative bias, allowing reliable assessment of relative differences in repeat composition among taxa. Therefore, our conclusions are primarily based on comparative patterns and major trends in repeat abundance, rather than on absolute quantitative estimates.

*Saccharum officinarum* exhibited remarkably homogeneous repeat content. This consistency aligns with the known genetic stability and fixed ploidy level (2n = 8x = 80) and domestication history (Piperidis and D’Hont, 2020). In contrast, *S. spontaneum* exhibited a wider range of repeat proportions, reflecting its genomic plasticity and extensive cytotype variation (2n = 8x, 9x, 10x = 40 – 128) (Zhang et al., 2022). Genotype-specific repeats, such as PIF/Harbinger DNA transposon were detected exclusively in *S. spontaneum* SES196 and Mandalay. The emergence of such lineage-specific repeats may be related to the chromosomal restructuring that characterizes *S. spontaneum* evolution and wider geographic distribution. We hypothesize that such large-scale chromosomal rearrangements could have triggered the *de novo* amplification of specific TE families, such as PIF/Harbinger. TE amplification or *de novo* amplification due to chromosome rearrangements has also been noticed in *Aegilops* and *Phaseolus* (Raskina et al., 2008; Ferraz et al., 2023; Badaeva et al., 2025).

A key finding is the strong positive correlation between genome size and repetitive DNA content (r = 0.96, *p* < 0.001), with repeat accumulation explaining ~96% of the observed variation. This confirms that TE proliferation—driven primarily by LTR retrotransposons such as Tekay (Ty3 LTR-RTs) and SIRE (Ty1 LTR-RTs)—is the main force underlying genome expansion in *Saccharum* (Mhiri et al., 2022; Hassan et al., 2024). Similar patterns have been reported in other Poaceae such as *Oryza* (Dai et al., 2022) and in *Loliinae* (Moreno-Aguilar et al., 2022).

In *S. officinarum*, *S. robustum*, and *S. edule*, all fixed at 2n = 8x = 80 and x = 10, repeat content ranges from 48–52%, consistent with TE expansion following polyploidization. In *S. spontaneum*, genotypes with higher chromosome numbers (>100; e.g., Glagah’, Mandalay’, SES517’) contained ~43% repetitive DNA, whereas those with fewer chromosomes (e.g., SES19., 54 chromosomes; Np-X’, 40 chromosomes) contained ~46%. Whole-genome duplication (WGD) triggers genetic and epigenetic regulatory adjustments required to stabilize genome function (Bird et al., 2018; Edger et al., 2019), as well as potential disruptions to genome integrity caused by WGD (Scarlett et al., 2023).

In some angiosperms, rapid repeat expansion occurs after polyploidization (Chen et al., 2020). In others, polyploid genomes maintain repeat contents similar to those of their diploid progenitors (McCann et al., 2018). In a third scenario, high-level polyploids exhibit substantial repeatome reduction relative to diploid and low-level polyploid relatives (Parisod et al., 2010; Moreno-Aguilar et al., 2022). *Saccharum spontaneum* appears to follow the latter pattern; despite some genotypes reaching 2n = 14x = 112, their repeat content remains comparatively low. This suggests a history of genome contraction or efficient suppression of TE proliferation—phenomena observed in other polyploid systems where mechanisms such as non-homologous end joining and single-strand annealing promote repeat removal (Pulido and Casacuberta, 2023; Hyvönen et al., 2025). Thus, the “*Saccharum* complex” exemplifies the evolutionary balance between TE-driven genome expansion and contraction, with distinct lineages following divergent trajectories. The repeatome also records hybridization events, clearly evidenced in modern sugarcane cultivars. Comparative clustering and PCA analyses show that cultivars form an intermediate group between *S. officinarum* and *S. spontaneum*, retaining the major TE lineages from both progenitors. Analysis of LTR-RT complete genomes using DANTE shows that in the cultivar R570, LTR-RT composition closely mirrors *S. officinarum*, with high proportions of SIRE and Tekay elements. This reflects the nobilization process, in which successive backcrossing to *S. officinarum* enriched its genome contribution to ~80% (D’Hont et al., 1996; Piperidis and D’Hont, 2020). The bimodal LTR identity distributions observed within the Athila and Angela of R570 likely reflect distinct element populations inherited from their divergent parental subgenomes. Additionally, the presence of rare cultivar-specific repeat clusters suggests post-hybridization restructuring, and artificial selection mobilized or preserved certain TE families, leaving a repeat-based domestication signature.

SatDNA analysis revealed extensive diversity, identifying 91 high-confidence satDNA families. Their number and abundance varied markedly, from 5 in SsinUba, cvSP803 and cvQ208 to 15 SatDNA in *M. floridulus*, with total satDNA content ranging from 0.70% to 6.35% in cv. Q208 and *S. spontaneum* SES196, respectively. Despite this variability, a striking pattern of conservation emerged: a 137 bp satDNA homologous to the CENT2 repeat (Vieira et al., 2018; Huang et al., 2021), was identified in all genotypes. Its conservation across ~1.5–2 million years of divergence (Jannoo et al., 2007; Pompidor et al., 2021), underscores its essential role in centromere function. Given that centromeric satDNA is subject to strong evolutionary constraint (Naish and Henderson, 2024), its prevalence across the “*Saccharum* complex” highlights a conserved centromeric architecture that remains stable despite dynamic changes in other repeat families and the extensive polyploidy and aneuploidy in these genomes.

Phylogenetic reconstruction based on similarity profiles of the 100 most abundant repeat clusters largely recapitulated known relationships within the “*Saccharum* complex” (Garsmeur et al., 2025). *Miscanthus* species, along with *S. narenga* and *E. fulvus*, formed a distinct outgroup. *S. spontaneum* genotypes clustered into two well-supported monophyletic groups, with structure mirroring the intraspecific diversity revealed by cluster analysis. Meanwhile, *S. officinarum*, *S. robustum*, *S. edule*, *S. barberi*, *S. sinense*, and modern cultivars clustered together, reflecting their shared recent evolutionary history. The cultivar cvSP803 was the only genotype not included in this cluster, which may be associated with the absence of 5S rDNA and reduced abundance of 35S rDNA. These results highlight the capacity of repeat-based phylogenomics to resolve relationships within recently diverged, hybridization-rich groups. Since repeats represent abundant, rapidly evolving genomic features that are less prone to homogenization through breeding than single-copy genes, they can express deeper architectural signals (Dodsworth et al., 2015; Vitales et al., 2020).

Repeat-based phylogeny differs from conventional phylogenetic analyses in that it is based on the composition and abundance of repetitive DNA rather than on homologous gene sequences. Conventional phylogenetic reconstruction typically relies on aligned nuclear or organellar gene sequences and reflects species evolutionary history through nucleotide substitutions and vertical inheritance. In contrast, repeat-based phylogeny uses genome-wide repeat profiles, including transposable elements and satellite DNA, which evolve primarily through amplification, deletion, and homogenization processes. As a result, repeat-based phylogenies reflect genome evolution dynamics, such as transposable element bursts, genome size variation, polyploidization, and chromosomal rearrangements, rather than strictly species divergence. Consequently, the topology obtained from repeat-based analyses may differ from conventional phylogenies and usually provides lower taxonomic resolution, but it is particularly useful for studying genome evolution and relationships among closely related or polyploid species (Novák et al., 2010; Novák et al., 2013; Dodsworth et al., 2015; Vitales et al., 2020).

## 5 Conclusion

This comprehensive comparative analysis of 30 accessions spanning the “*Saccharum* complex”, provides an extensive characterization of repeatome diversity. Repetitive DNA—particularly LTR retrotransposons—comprise the dominant component of these large, highly polyploid genomes, and its abundance varies predictably with genome size. Although *S. officinarum* displays a highly conserved and homogeneous repeat composition consistent with domestication and stable ploidy, *S. spontaneum* exhibits heterogeneity regarding certain repeats, reflecting its cytotypic range and geographical dispersion history. Cultivars retain repeat signatures from both progenitors, with SIRE and Tekay contributing significantly to their shared genomic architecture. SatDNA reveal a mixture of conserved and lineage-specific families, including a prevalent 137 bp centromeric repeat, indicating strong conservation of centromeric structure across *Saccharum*. Repeat-based phylogenetic inference successfully recovers major taxonomic groupings, corroborating species relationships inferred from whole genome sequence data. Structural annotation of LTR-RTs in fully assembled genomes further demonstrates lineage-specific dynamics, with SIRE and Tekay showing evidence of recent or ongoing activity, particularly in *S. officinarum* and modern cultivars. Overall, our findings illustrate how the repeatome has shaped genome, divergence, and hybridization outcomes in *Saccharum*.

## Data Availability

The datasets presented in this study can be found in online repositories. The names of the repository/repositories and accession number(s) can be found in the article/**Supplementary Material**.

## Supplementary material

Supplementary Figure 1 Contribution of major repetitive element classes to PCA analysis. Higher absolute loading values indicate greater influence of that repeat class on the respective axis.
