# Supplementary figures and images for: The repeatome landscape in the “Saccharum complex”
Source: Front Plant Sci. 2026 May 21;17:1809735. doi: 10.3389/fpls.2026.1809735 (PMC13233458; doi:10.3389/fpls.2026.1809735)

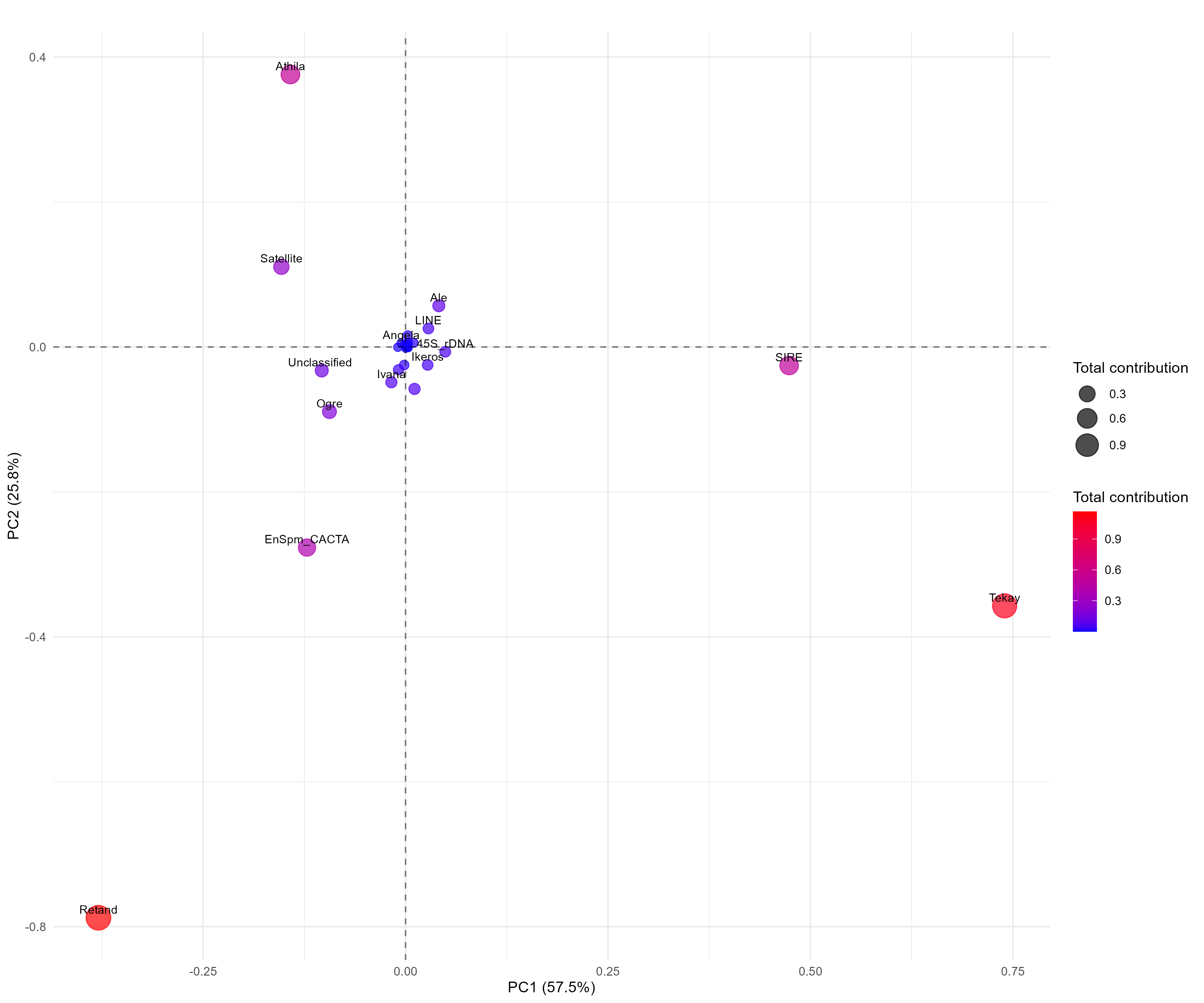

Supplement: Supplementary Figure 1 — Contribution of major repetitive element classes to PCA analysis. Higher absolute loading values indicate greater influence of that repeat class on the respective axis. [file Image1.jpeg]
