# Supplementary material for: The repeatome landscape in the “Saccharum complex”
Source: Front Plant Sci. 2026 May 21;17:1809735. doi: 10.3389/fpls.2026.1809735 (PMC13233458; doi:10.3389/fpls.2026.1809735)
Supplement: Supplementary file 2 [file Presentation1.pdf]

Supplementary file 2 – Complete DANTE Analysis

S. officinarum

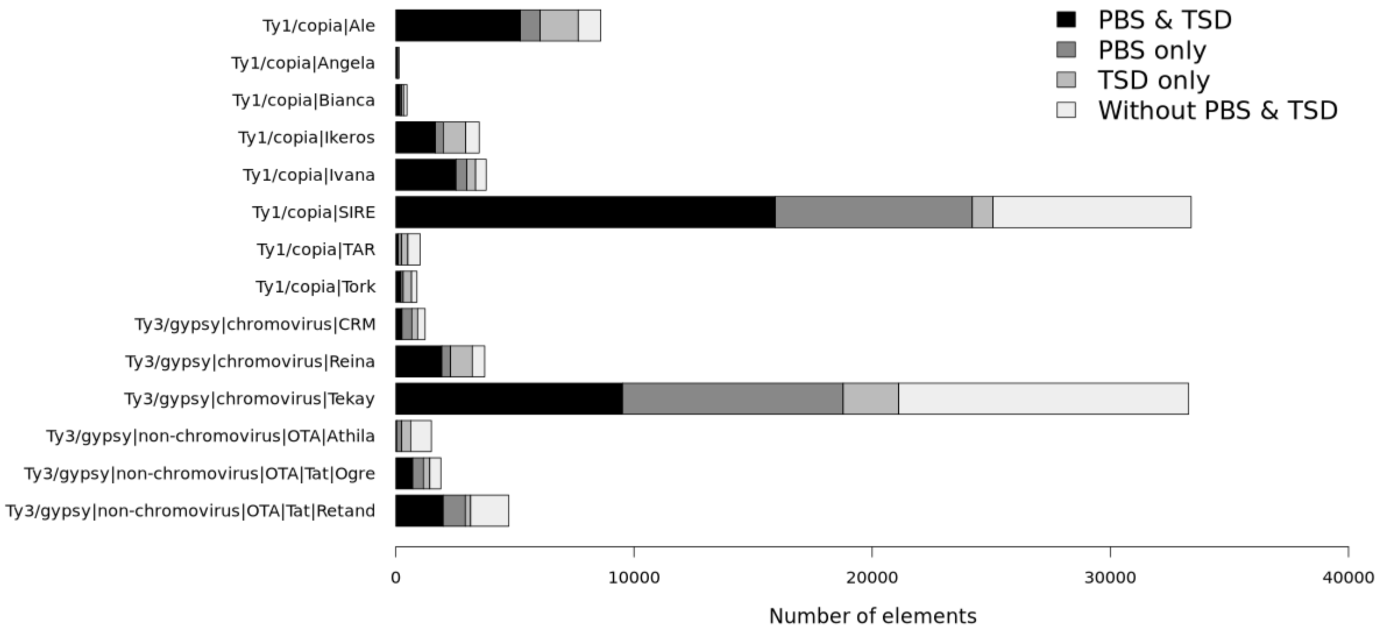

Number of elements of LTR-RT lineages in the complete genome of *Saccharum officinarum*.

General description of the supplementary figures below:

Each supplementary figure consists of two panels. The left panel shows the structural organization of the LTR lineage identified using DANTE, where each horizontal line represents an individual element aligned according to its length. Colored segments indicate conserved protein domains, including long terminal repeats (LTRs, black), GAG (orange), protease (PROT, pink), integrase (INT, green), reverse transcriptase (RT, blue), and RNase H (RH, red), while gray regions correspond to sequences lacking detectable domains. Most elements exhibit the canonical LTR retrotransposon structure (LTR–GAG–PROT–INT–RT–RH–LTR), although variation in length and domain composition is evident, reflecting structural diversity and different levels of element degradation.

The right panel presents summary statistics and structural features of the same elements. The upper panels show the distribution of element length, LTR length, and LTR identity, as well as the frequency of the most common primer binding sites (PBS). The middle panel displays the number of elements with missing domains, highlighting differential conservation among domains. The lower panel illustrates variation in domain size across elements and the consensus structure of the average element, including LTRs, GAG, PROT, INT, RT, and RH domains. Overall, the elements display a conserved canonical organization, with high LTR identity suggesting recent insertion events and notable variability in domain composition and size.

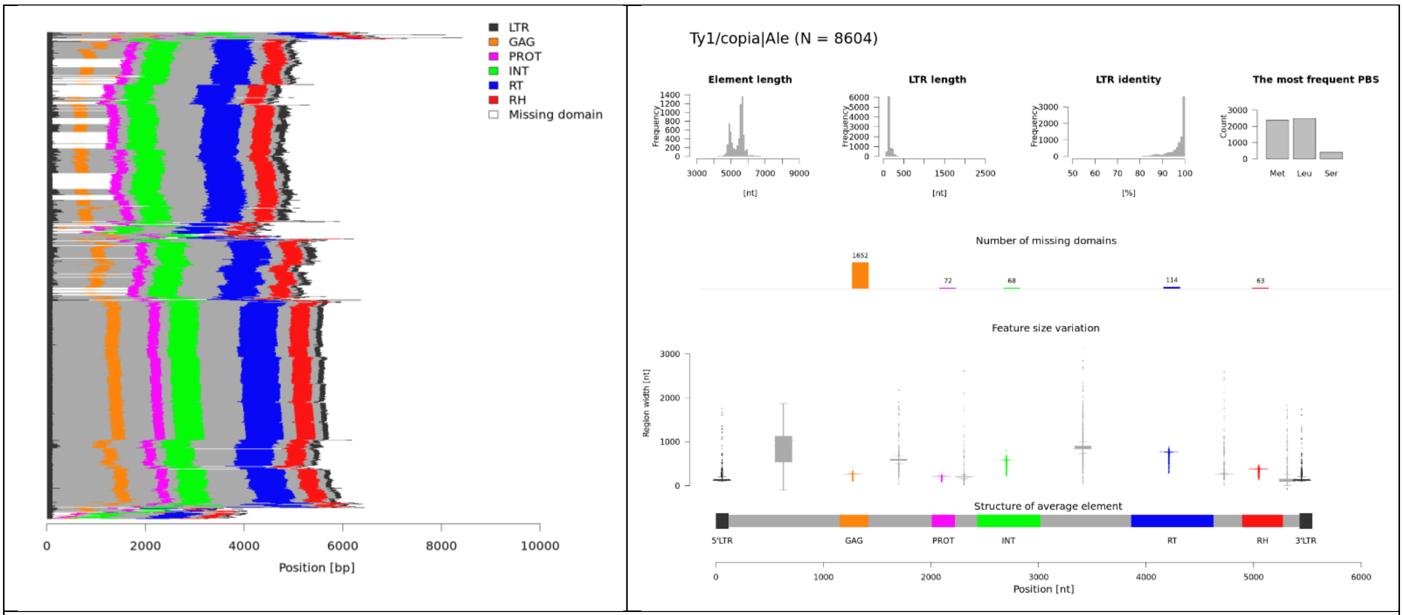

The left panel shows the structural organization of Ty1/copia-Ale elements, whereas the right panel presents the summary statistics and structural features of these elements.

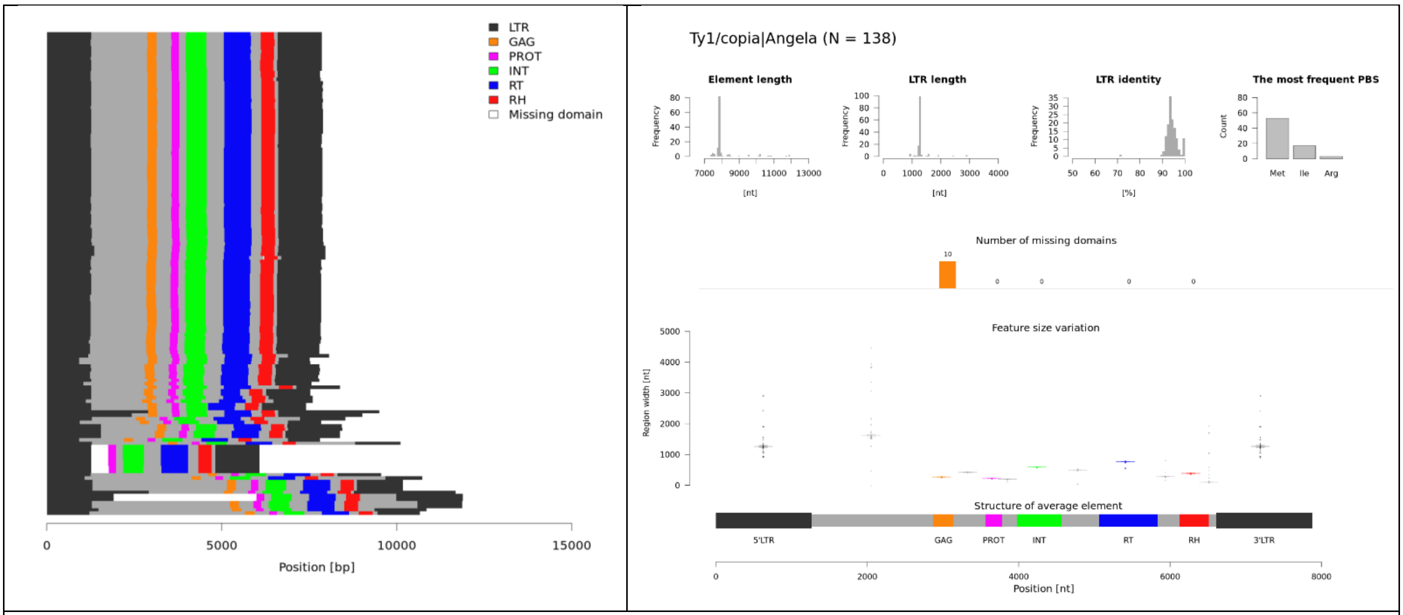

The left panel shows the structural organization of Ty1/copia-Angela elements, whereas the right panel presents the summary statistics and structural features of these elements.

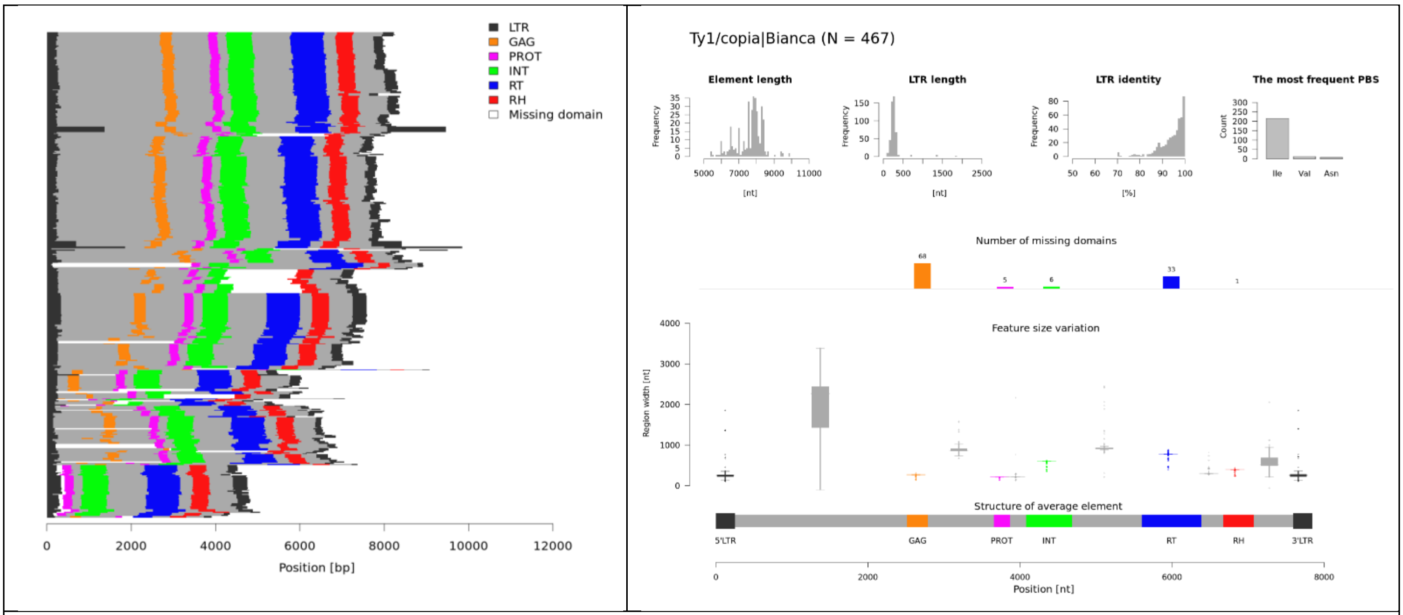

The left panel shows the structural organization of Ty1/copia-Bianca elements, whereas the right panel presents the summary statistics and structural features of these elements.

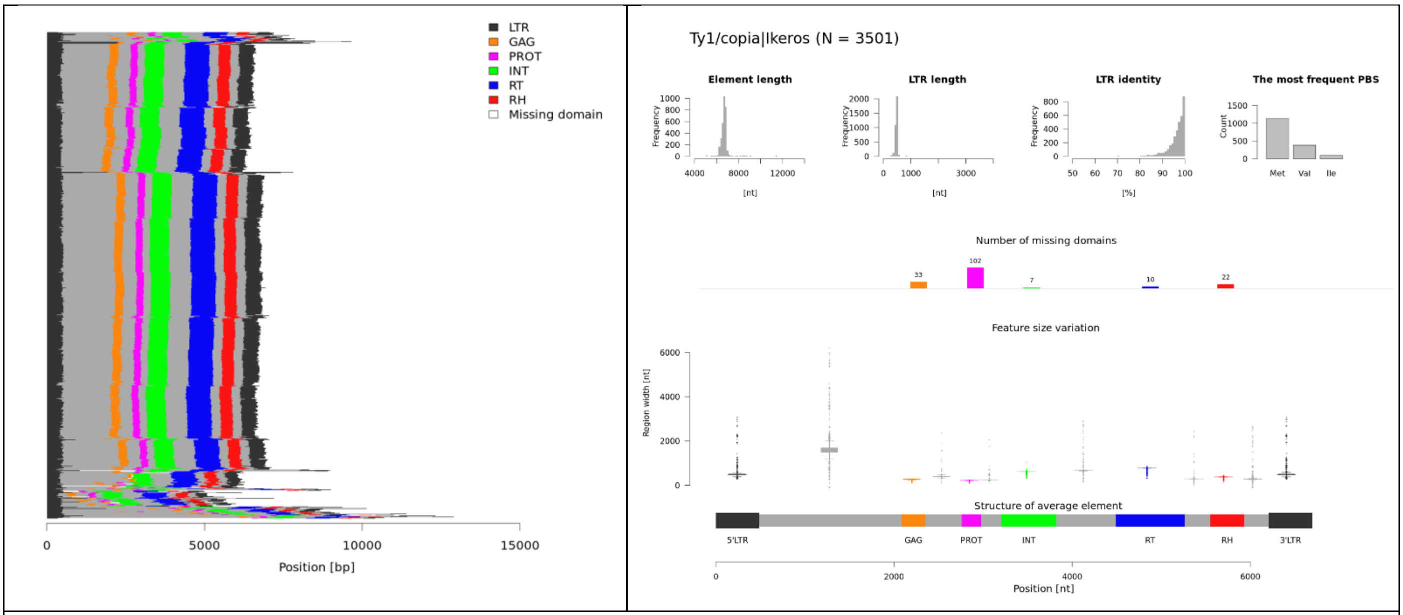

The left panel shows the structural organization of Ty1/copia-Ikeros elements, whereas the right panel presents the summary statistics and structural features of these elements.

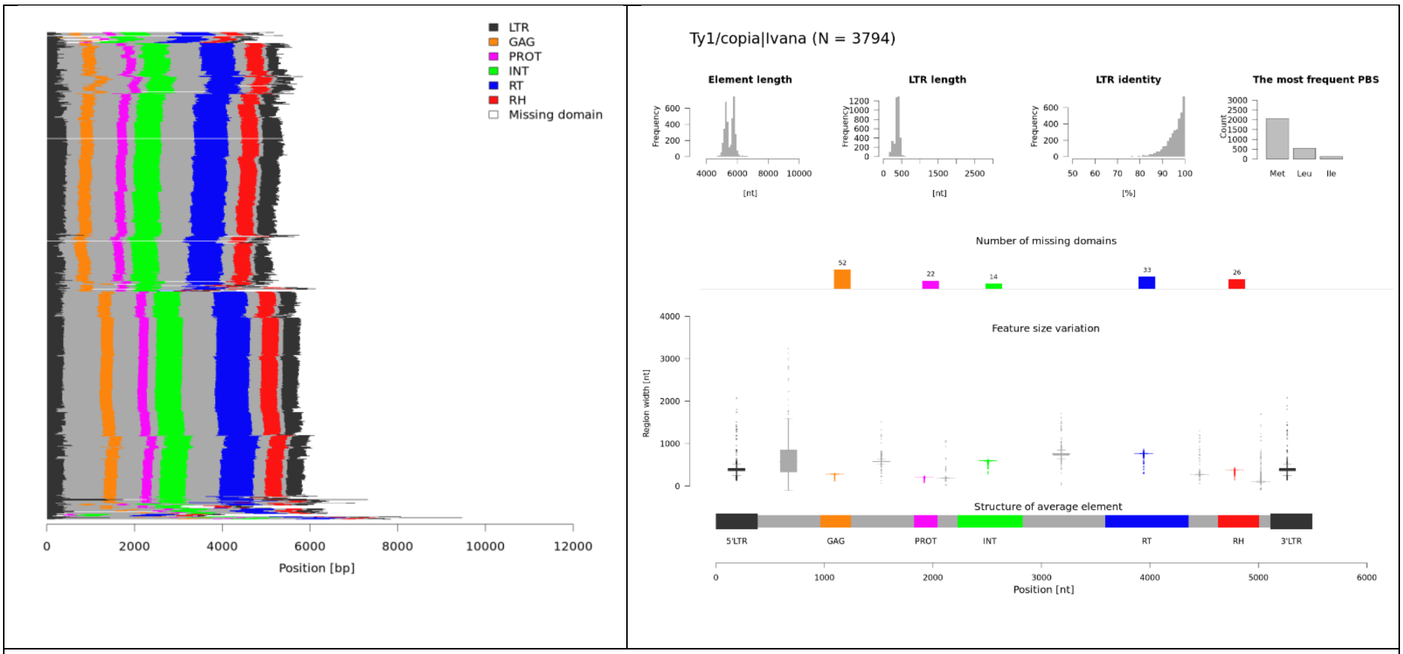

The left panel shows the structural organization of Ty1/copia-Ivana elements, whereas the right panel presents the summary statistics and structural features of these elements.

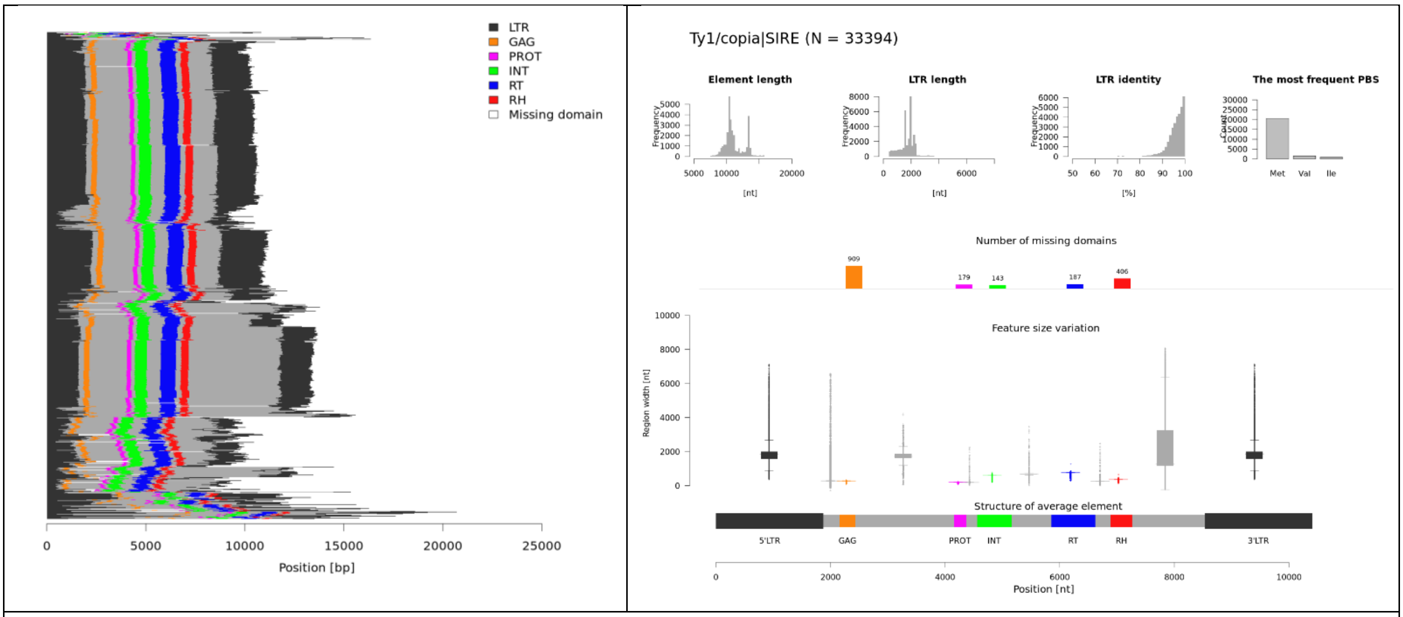

The left panel shows the structural organization of Ty1/copia-Sire elements, whereas the right panel presents the summary statistics and structural features of these elements.

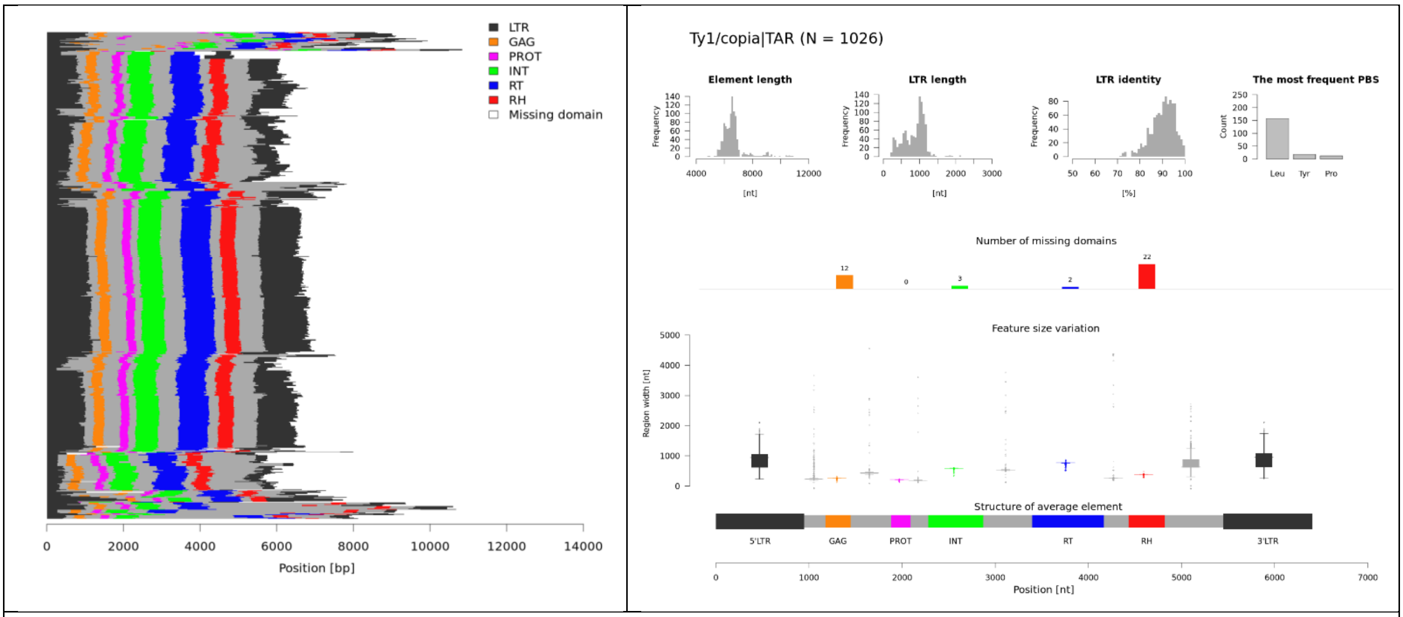

The left panel shows the structural organization of Ty1/copia-TAR elements, whereas the right panel presents the summary statistics and structural features of these elements.

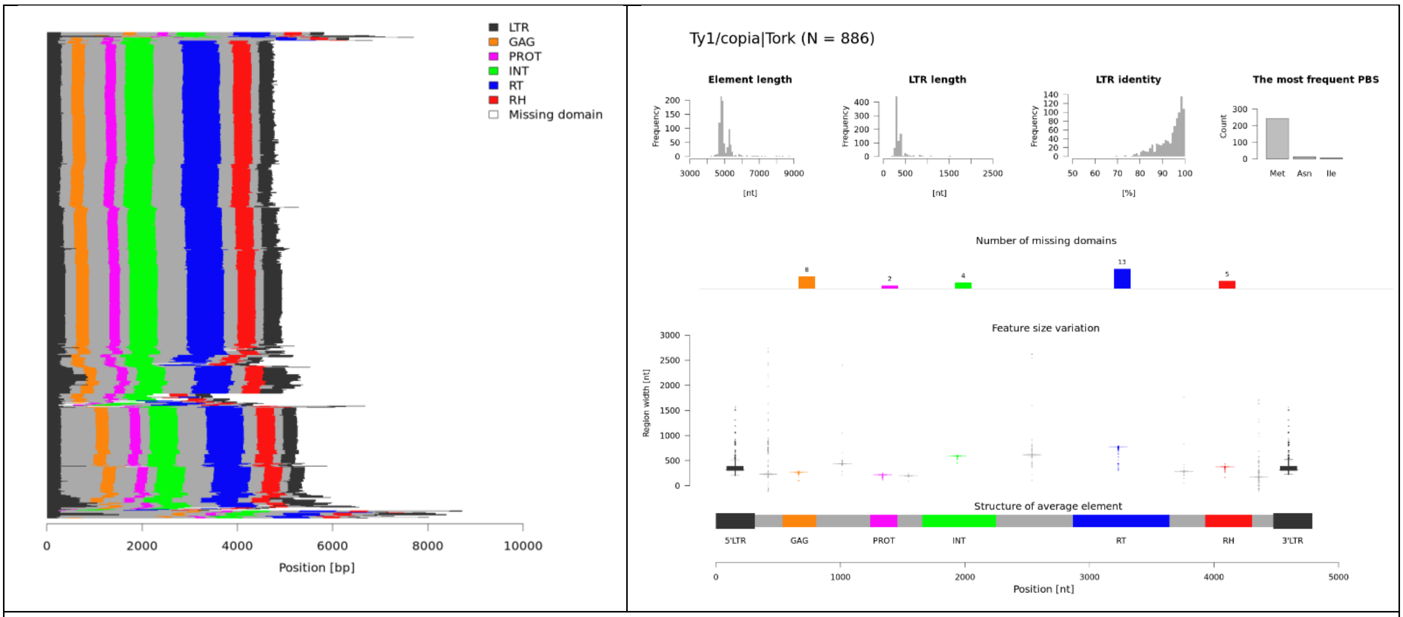

The left panel shows the structural organization of Ty1/copia-Tork elements, whereas the right panel presents the summary statistics and structural features of these elements.

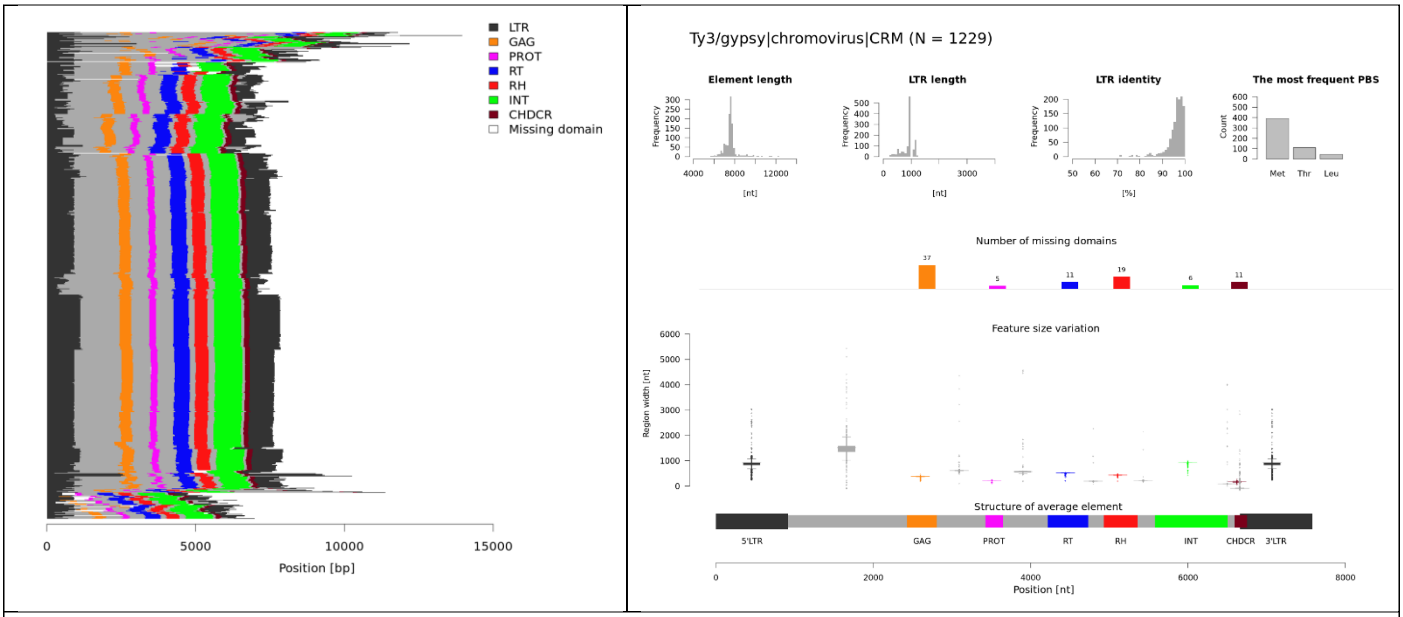

The left panel shows the structural organization of Ty3/gypsy-CRM elements, whereas the right panel presents the summary statistics and structural features of these elements.

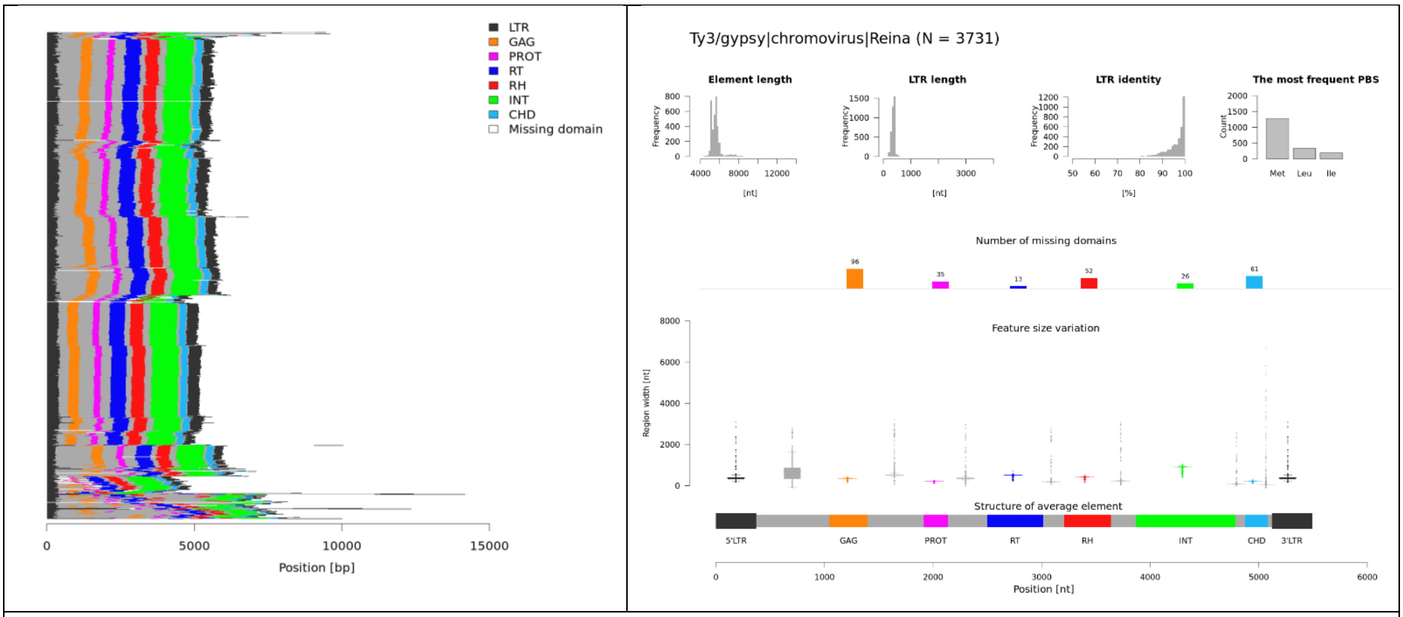

The left panel shows the structural organization of Ty3/gypsy-Reina elements, whereas the right panel presents the summary statistics and structural features of these elements.

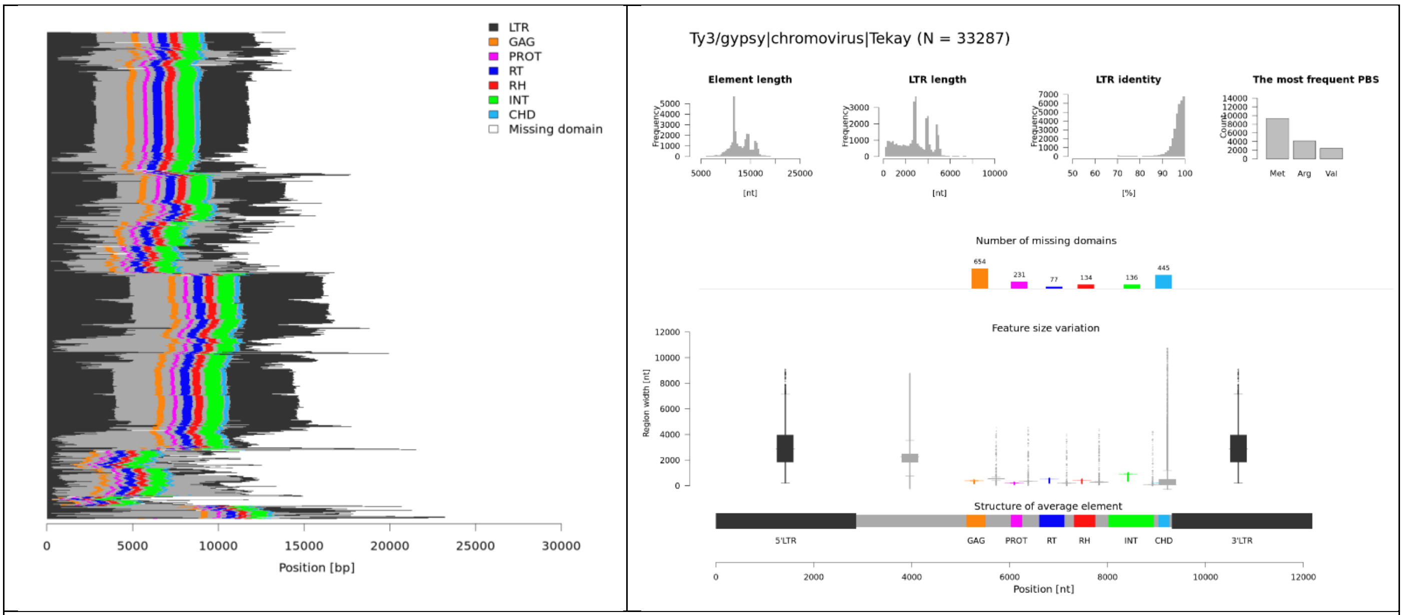

The left panel shows the structural organization of Ty3/gypsy-Tekay elements, whereas the right panel presents the summary statistics and structural features of these elements.

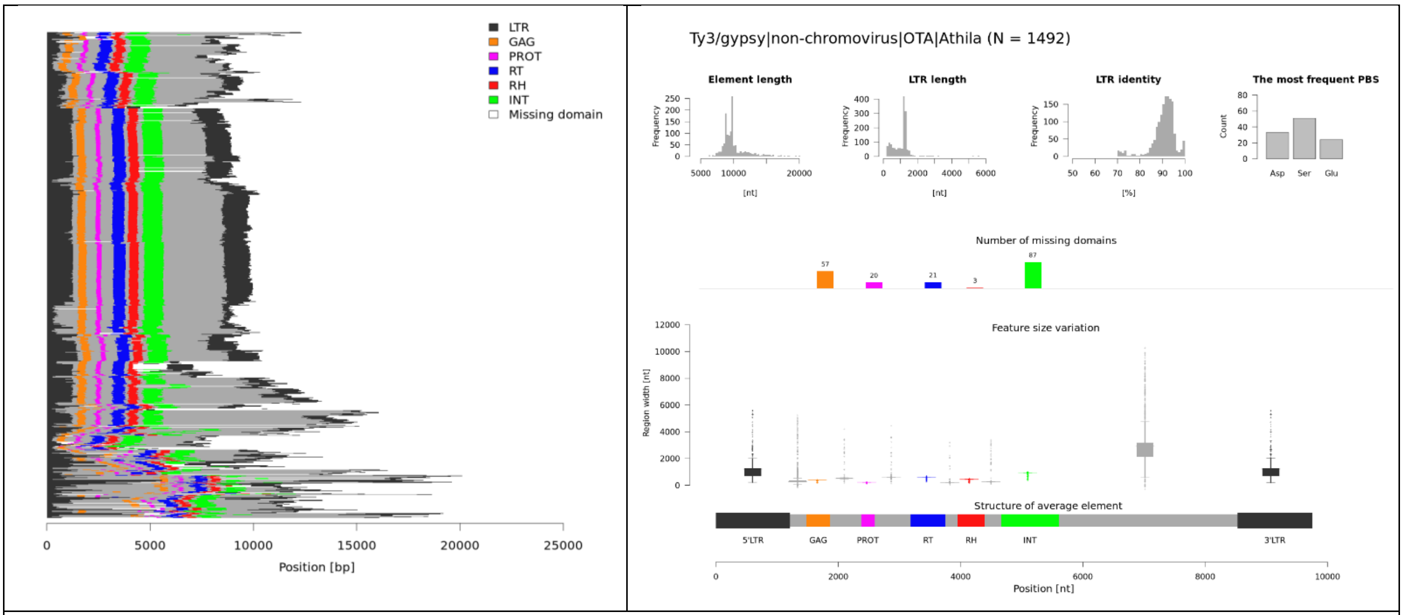

The left panel shows the structural organization of Ty3/gypsy-Athila elements, whereas the right panel presents the summary statistics and structural features of these elements.

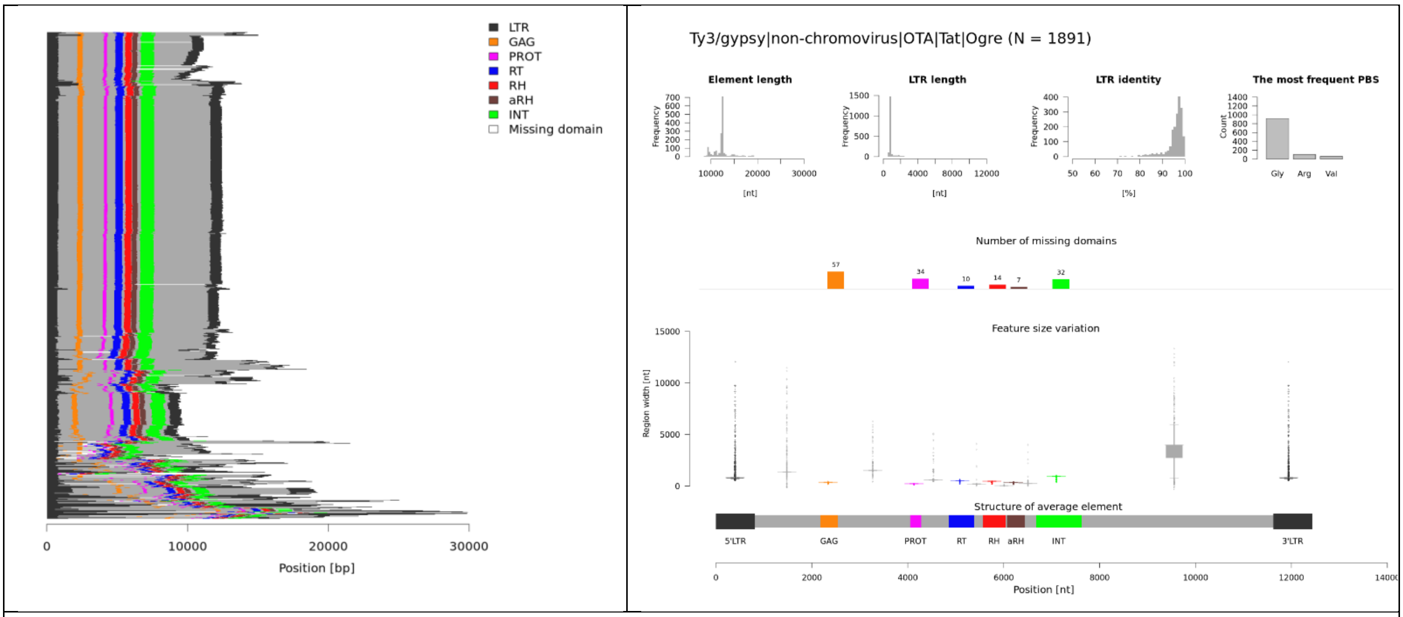

The left panel shows the structural organization of Ty3/gypsy-Ogre elements, whereas the right panel presents the summary statistics and structural features of these elements.

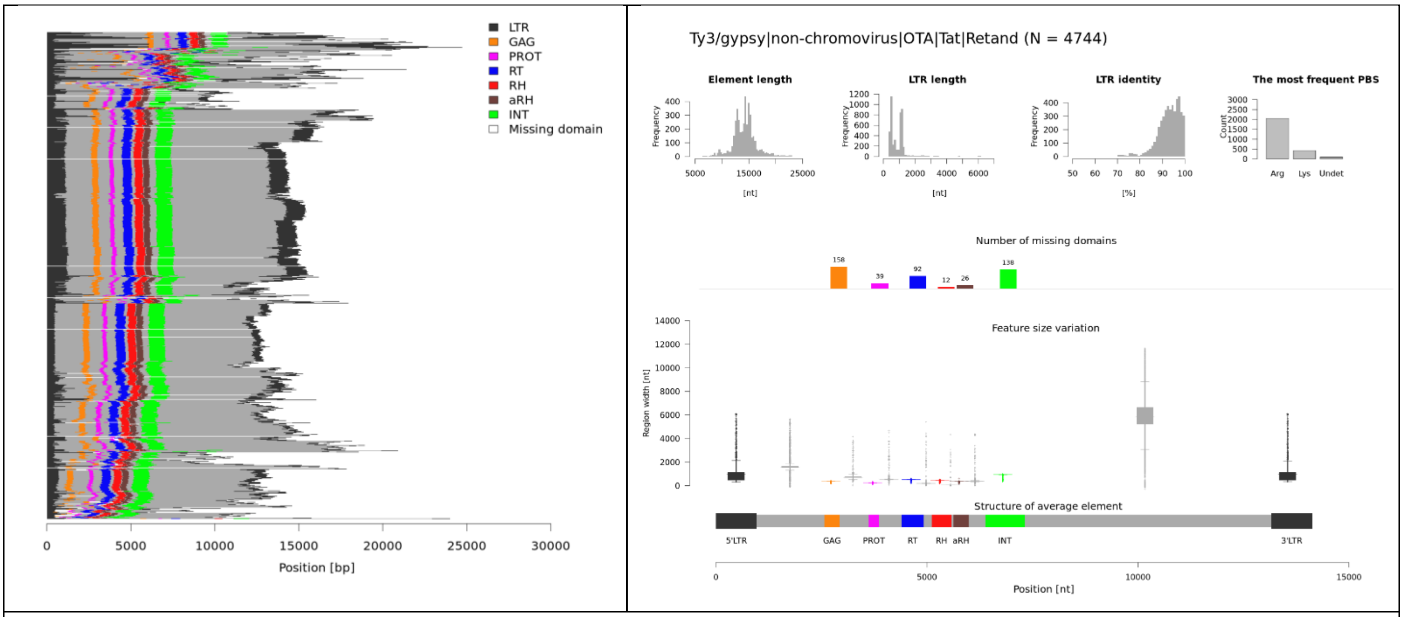

The left panel shows the structural organization of Ty3/gypsy-Retand elements, whereas the right panel presents the summary statistics and structural features of these elements.

***S. spontaneum* AP85-441**

Number of elements of LTR-RT lineages in the complete genome of *Saccharum spontaneum* AP85-441.

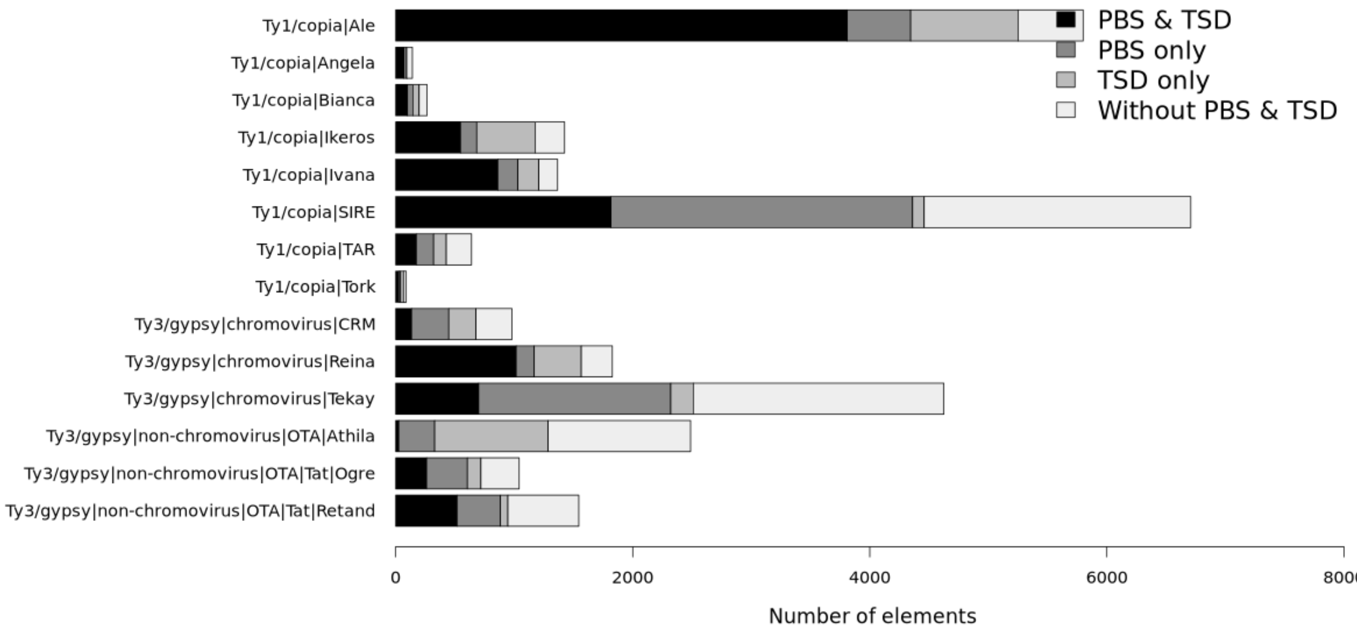

General description of the supplementary figures below:

Each supplementary figure consists of two panels. The left panel shows the structural organization of the LTR lineage identified using DANTE, where each horizontal line represents an individual element aligned according to its length. Colored segments indicate conserved protein domains, including long terminal repeats (LTRs, black), GAG (orange), protease (PROT, pink), integrase (INT, green), reverse transcriptase (RT, blue), and RNase H (RH, red), while gray regions correspond to sequences lacking detectable domains. Most elements exhibit the canonical LTR retrotransposon structure (LTR–GAG–PROT–INT–RT–RH–LTR), although variation in length and domain composition is evident, reflecting structural diversity and different levels of element degradation.

The right panel presents summary statistics and structural features of the same elements. The upper panels show the distribution of element length, LTR length, and LTR identity, as well as the frequency of the most common primer binding sites (PBS). The middle panel displays the number of elements with missing domains, highlighting differential conservation among domains. The lower panel illustrates variation in domain size across elements and the consensus structure of the average element, including LTRs, GAG, PROT, INT, RT, and RH domains. Overall, the elements display a conserved canonical organization, with high LTR identity suggesting recent insertion events and notable variability in domain composition and size.

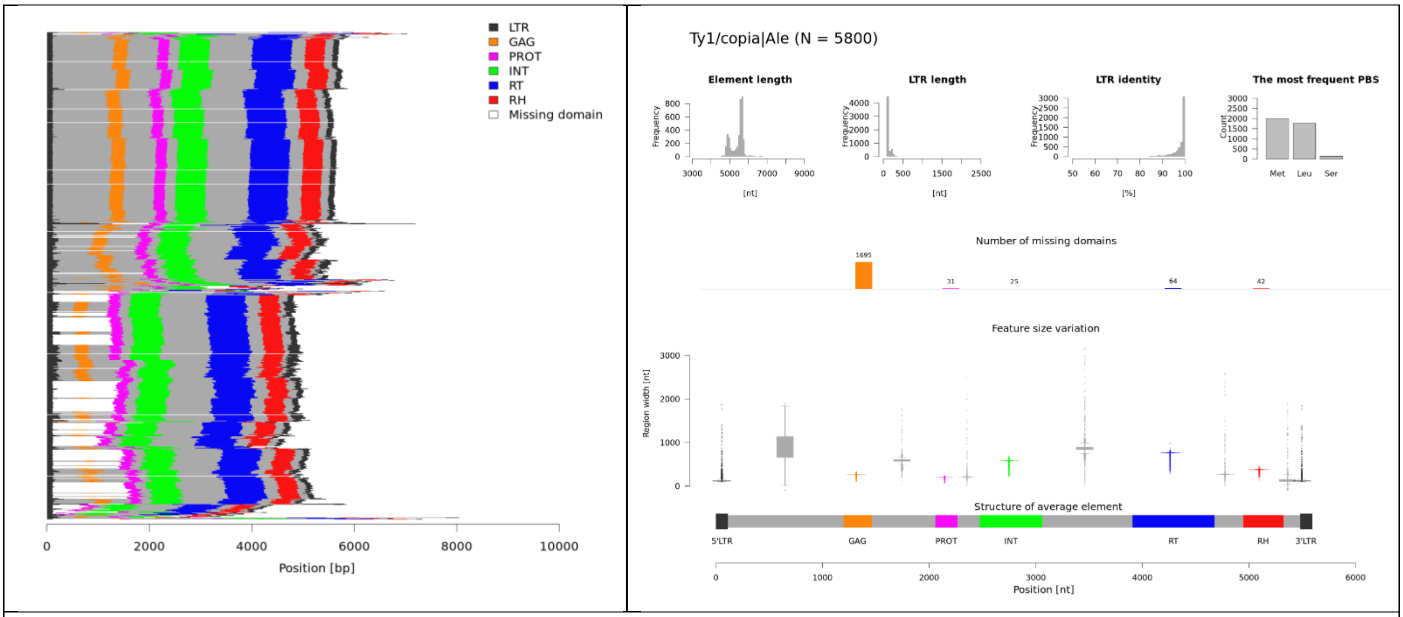

The left panel shows the structural organization of Ty1/copia-Ale elements, whereas the right panel presents the summary statistics and structural features of these elements.

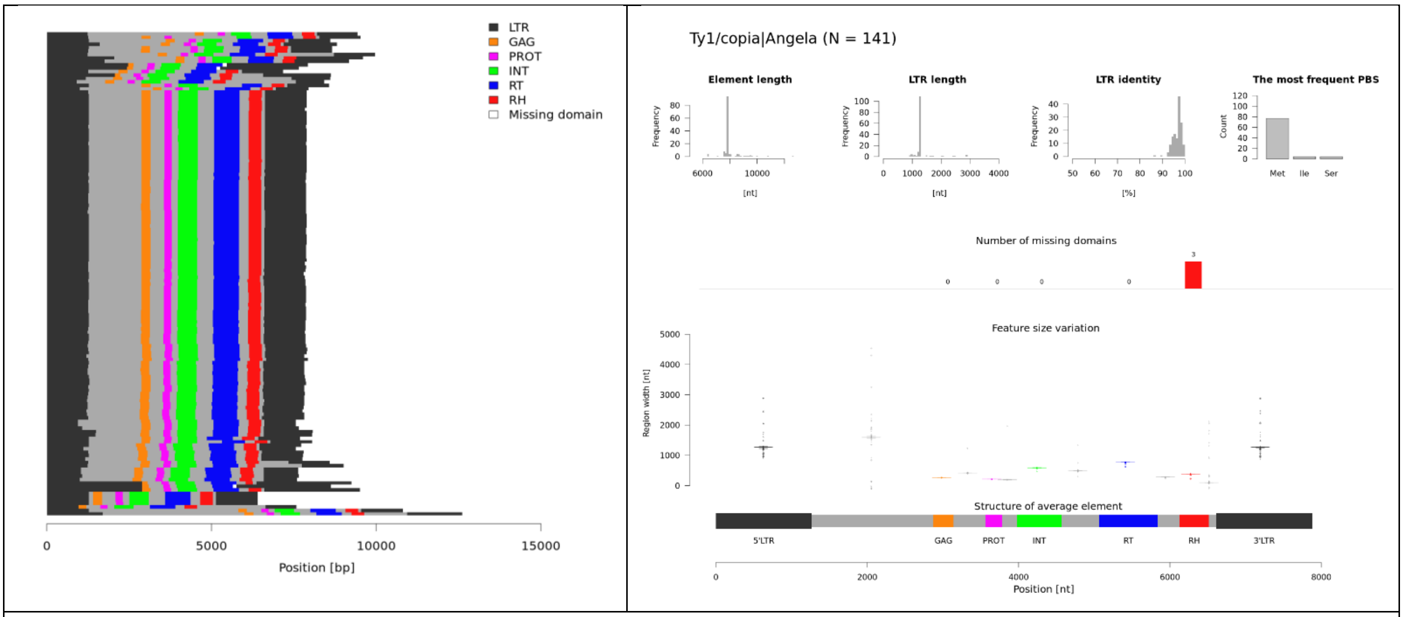

The left panel shows the structural organization of Ty1/copia-Angela elements, whereas the right panel presents the summary statistics and structural features of these elements.

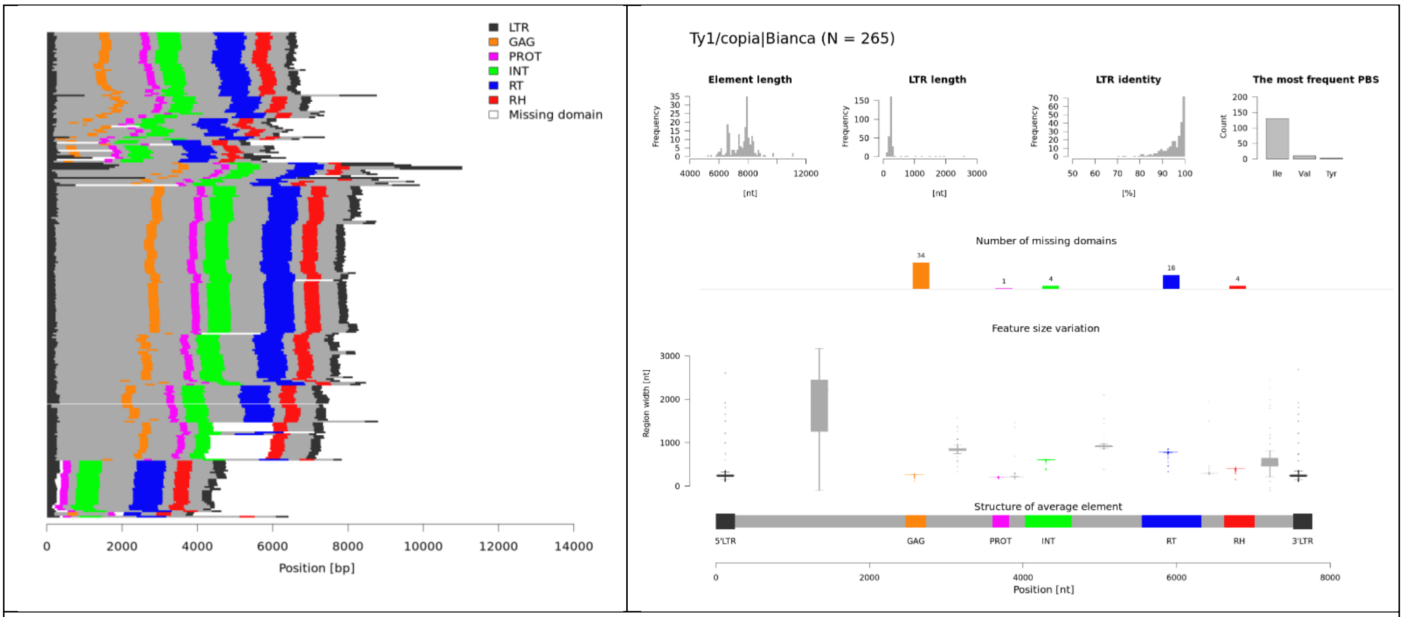

The left panel shows the structural organization of Ty1/copia-Bianca elements, whereas the right panel presents the summary statistics and structural features of these elements.

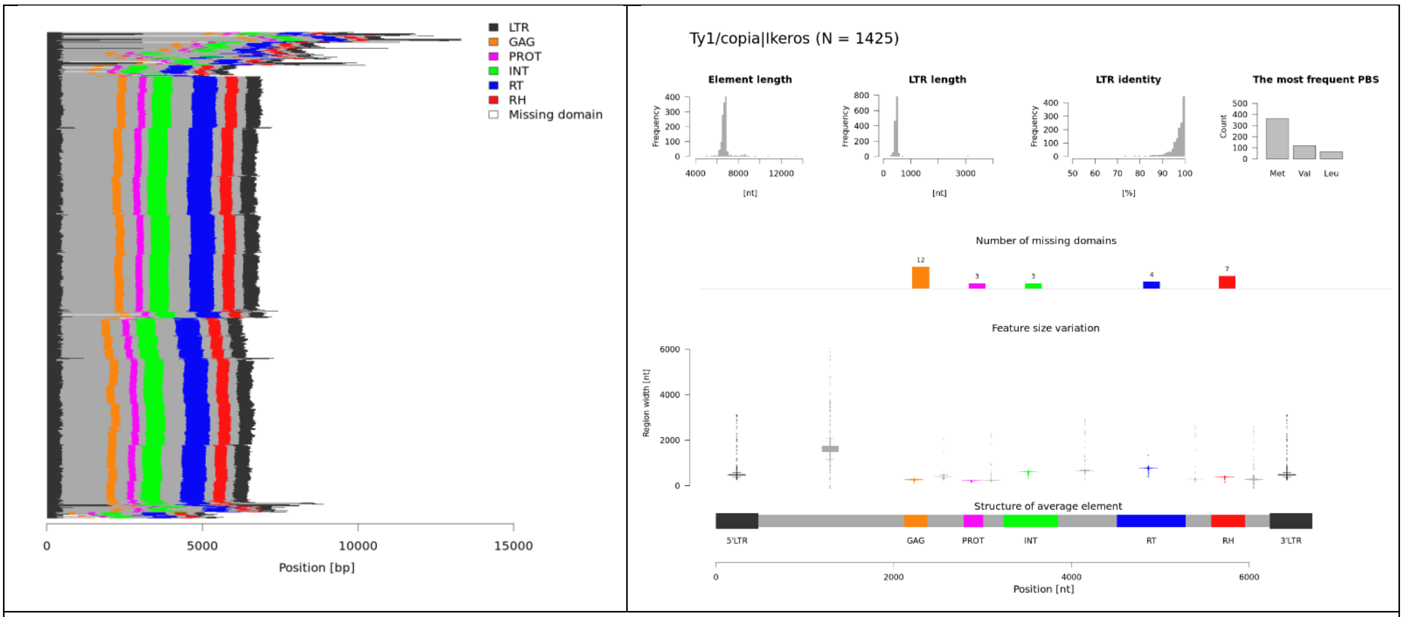

The left panel shows the structural organization of Ty1/copia-Ikeros elements, whereas the right panel presents the summary statistics and structural features of these elements.

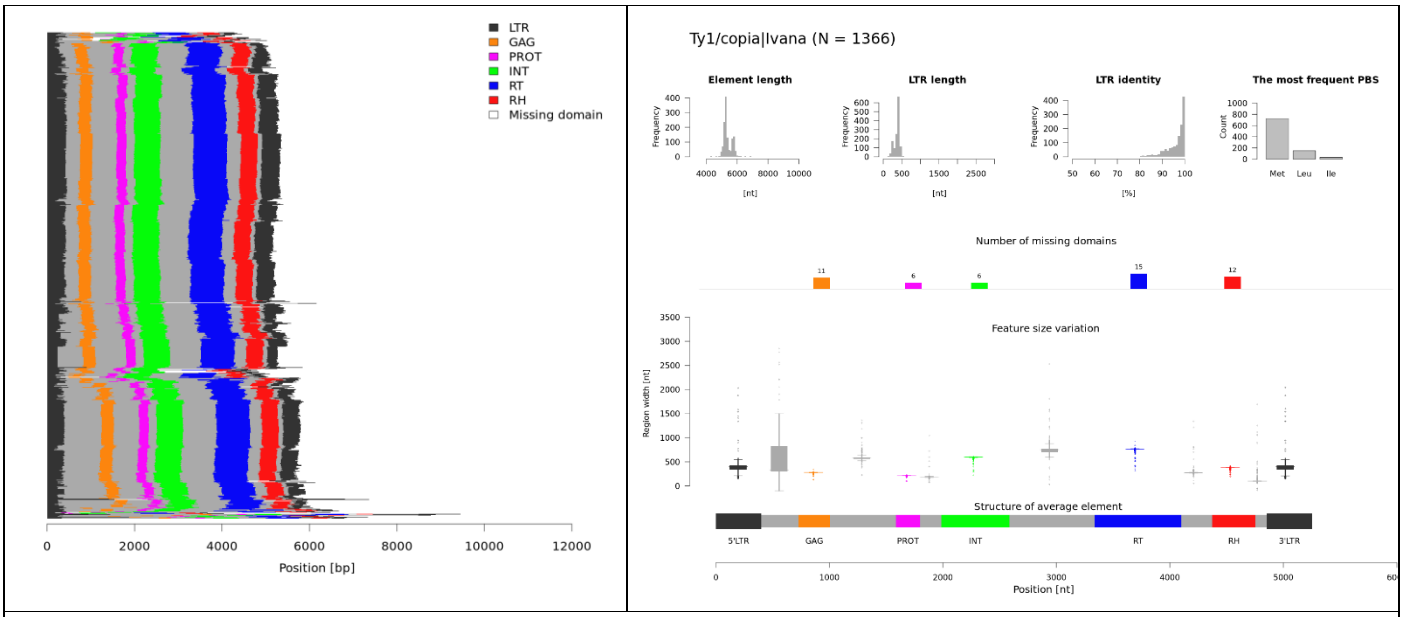

The left panel shows the structural organization of Ty1/copia-Ivana elements, whereas the right panel presents the summary statistics and structural features of these elements.

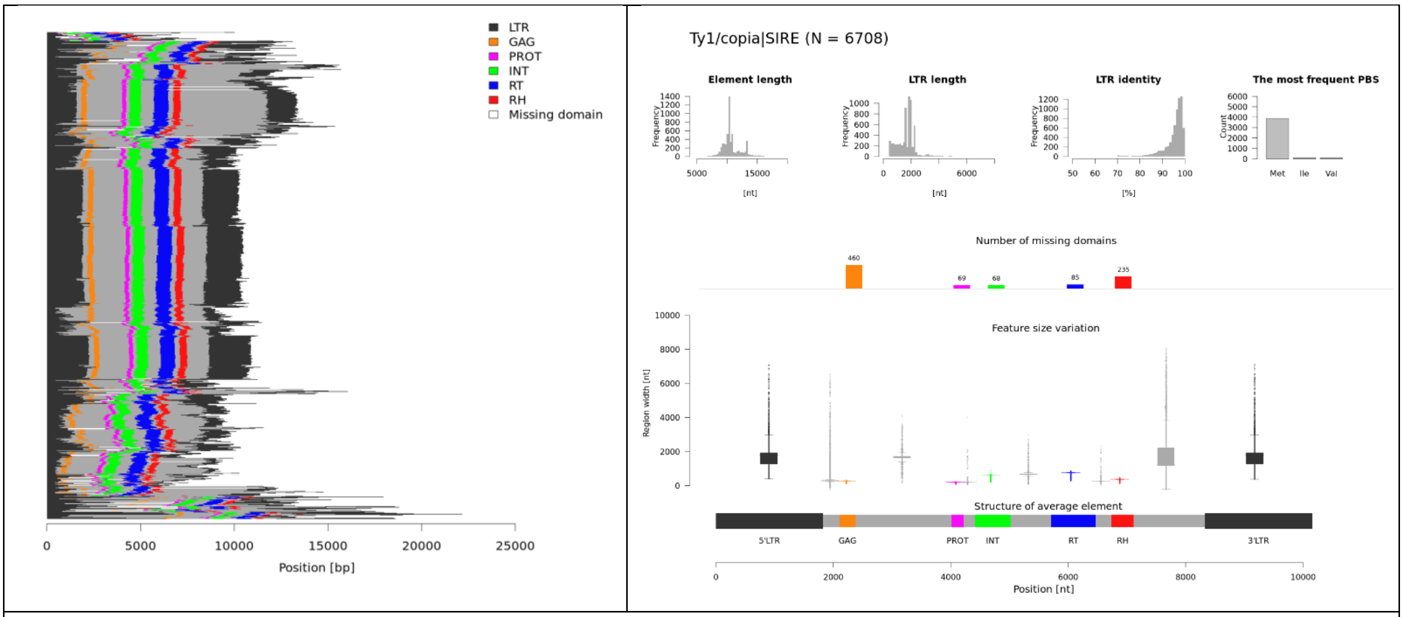

The left panel shows the structural organization of Ty1/copia-Sire elements, whereas the right panel presents the summary statistics and structural features of these elements.

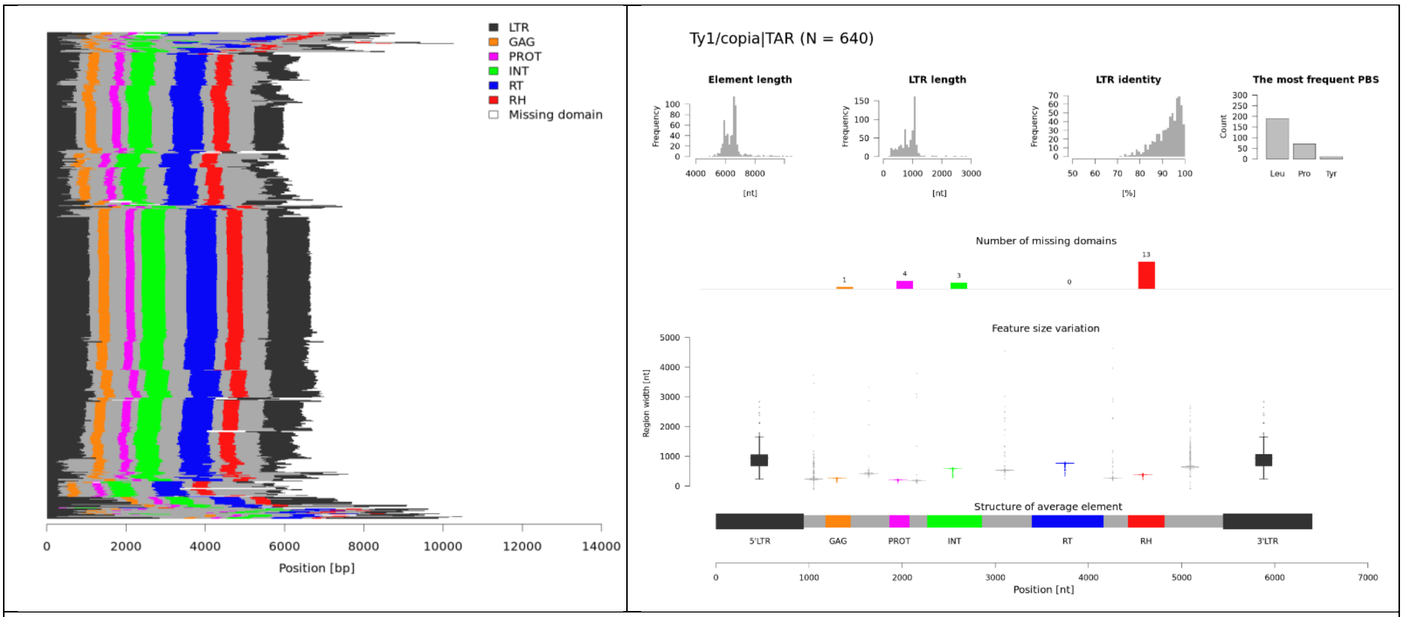

The left panel shows the structural organization of Ty1/copia-TAR elements, whereas the right panel presents the summary statistics and structural features of these elements.

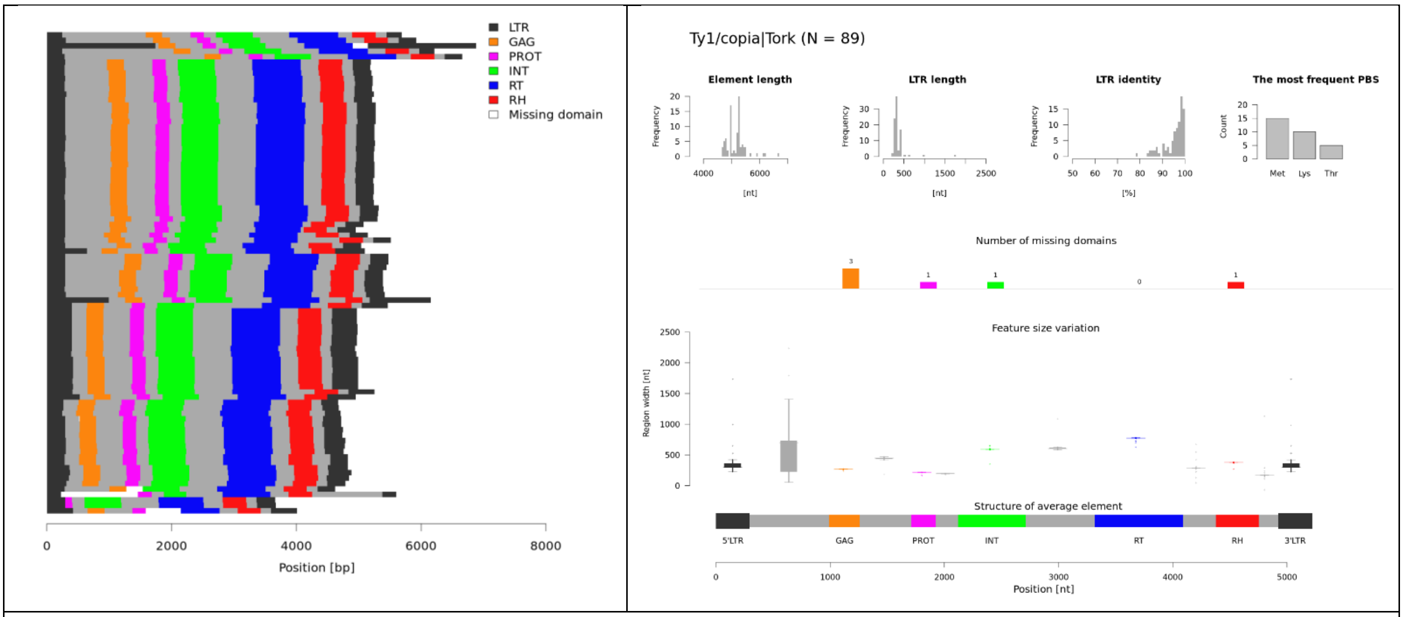

The left panel shows the structural organization of Ty1/copia-Tork elements, whereas the right panel presents the summary statistics and structural features of these elements.

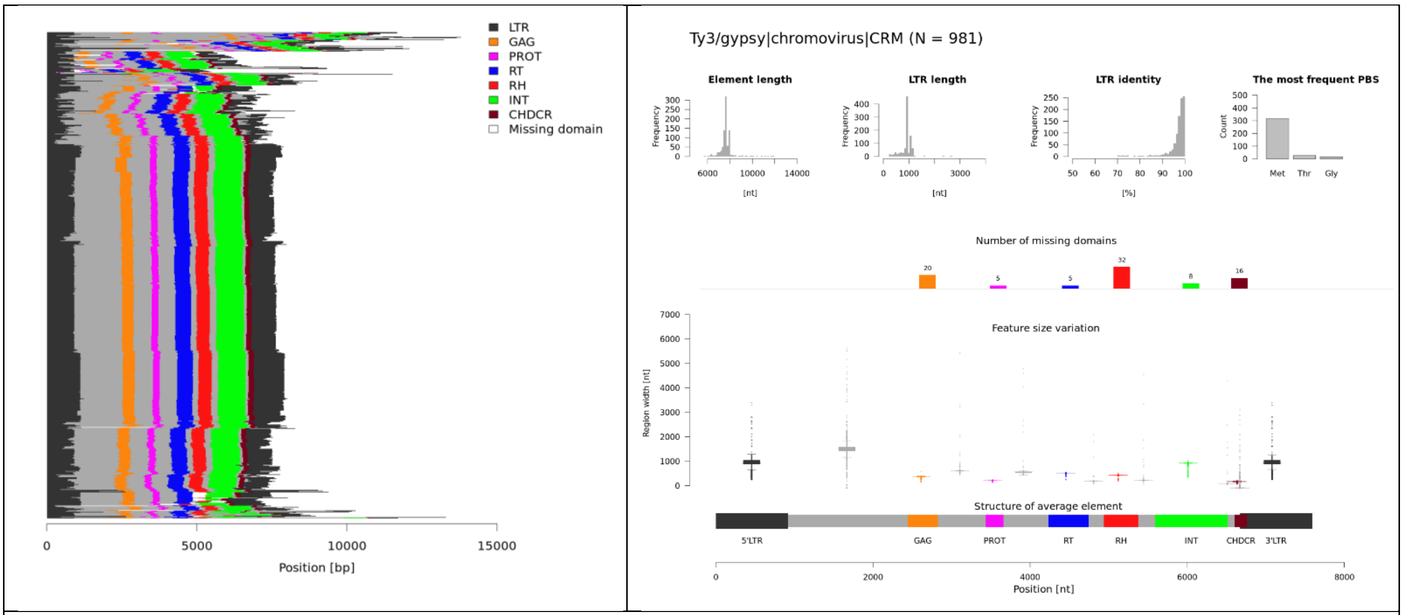

The left panel shows the structural organization of Ty3/gypsy-CRM elements, whereas the right panel presents the summary statistics and structural features of these elements.

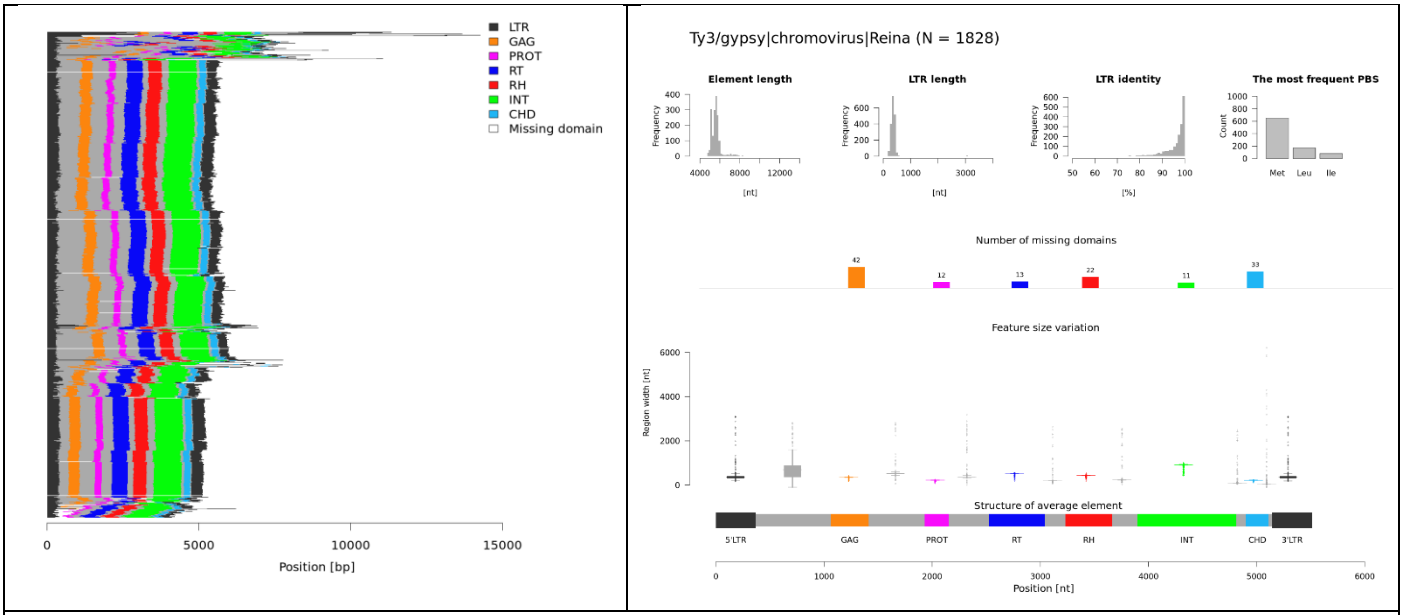

The left panel shows the structural organization of Ty3/gypsy-Reina elements, whereas the right panel presents the summary statistics and structural features of these elements.

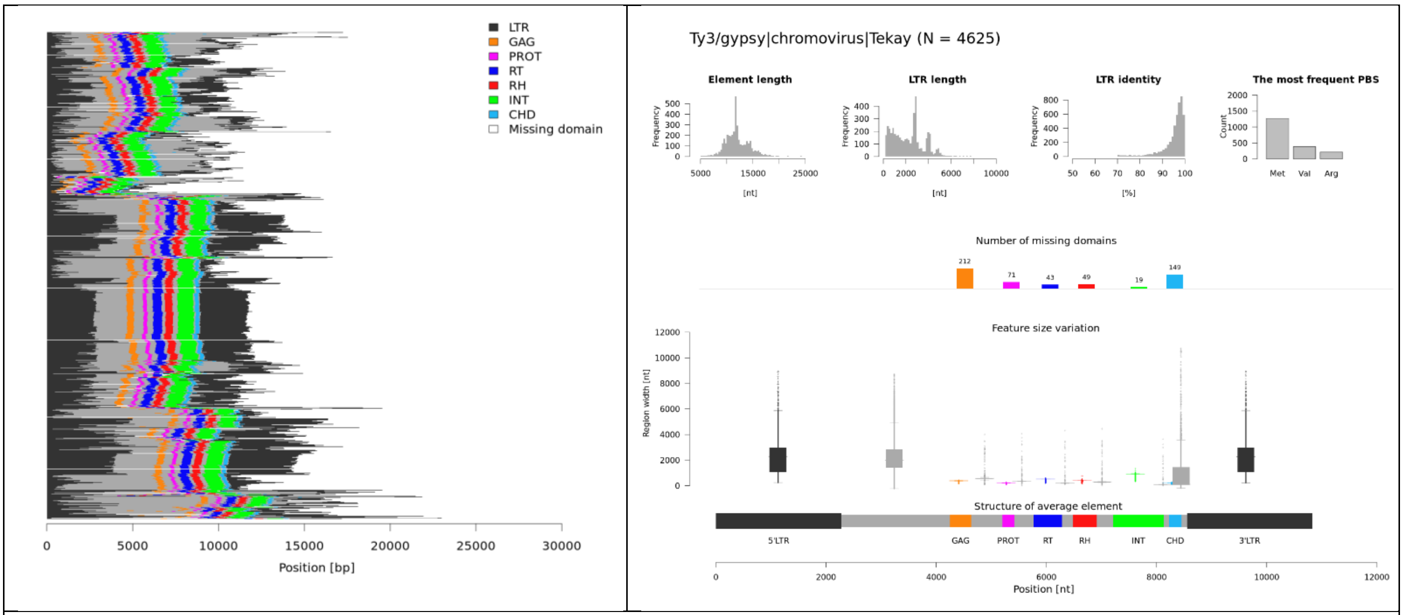

The left panel shows the structural organization of Ty3/gypsy-Tekay elements, whereas the right panel presents the summary statistics and structural features of these elements.

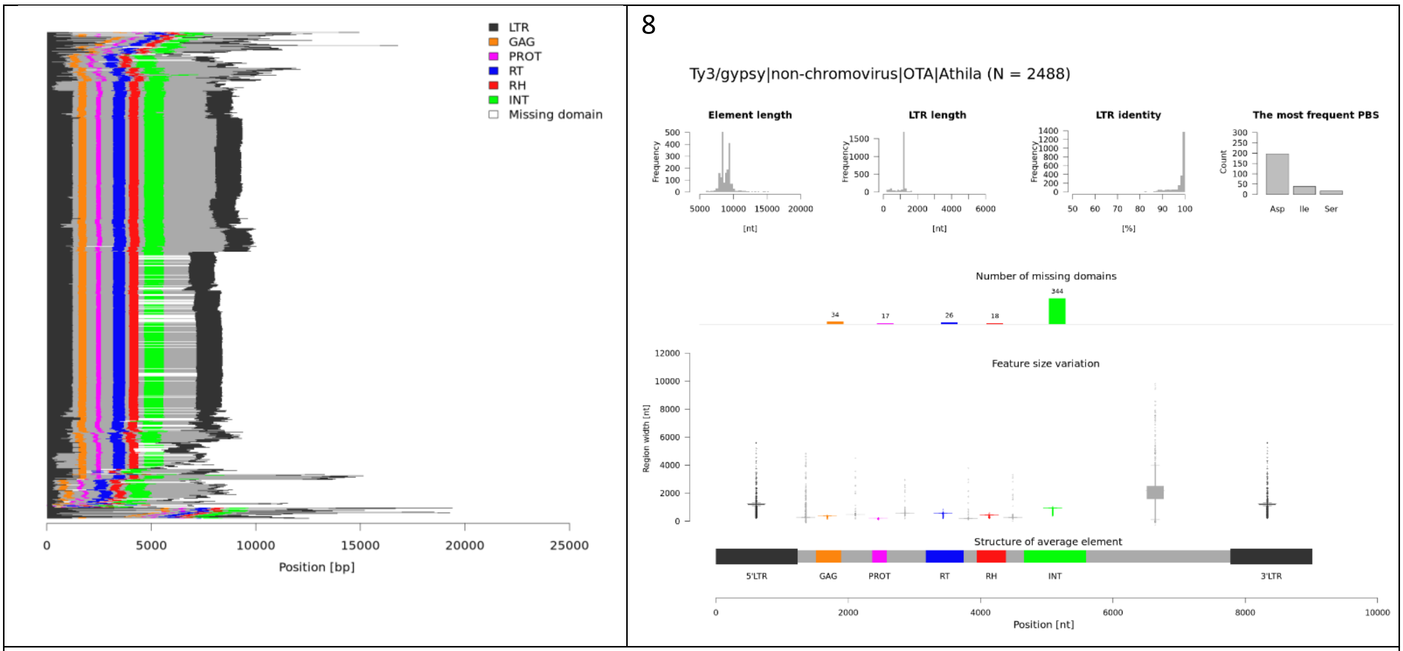

The left panel shows the structural organization of Ty3/gypsy-Athila elements, whereas the right panel presents the summary statistics and structural features of these elements.

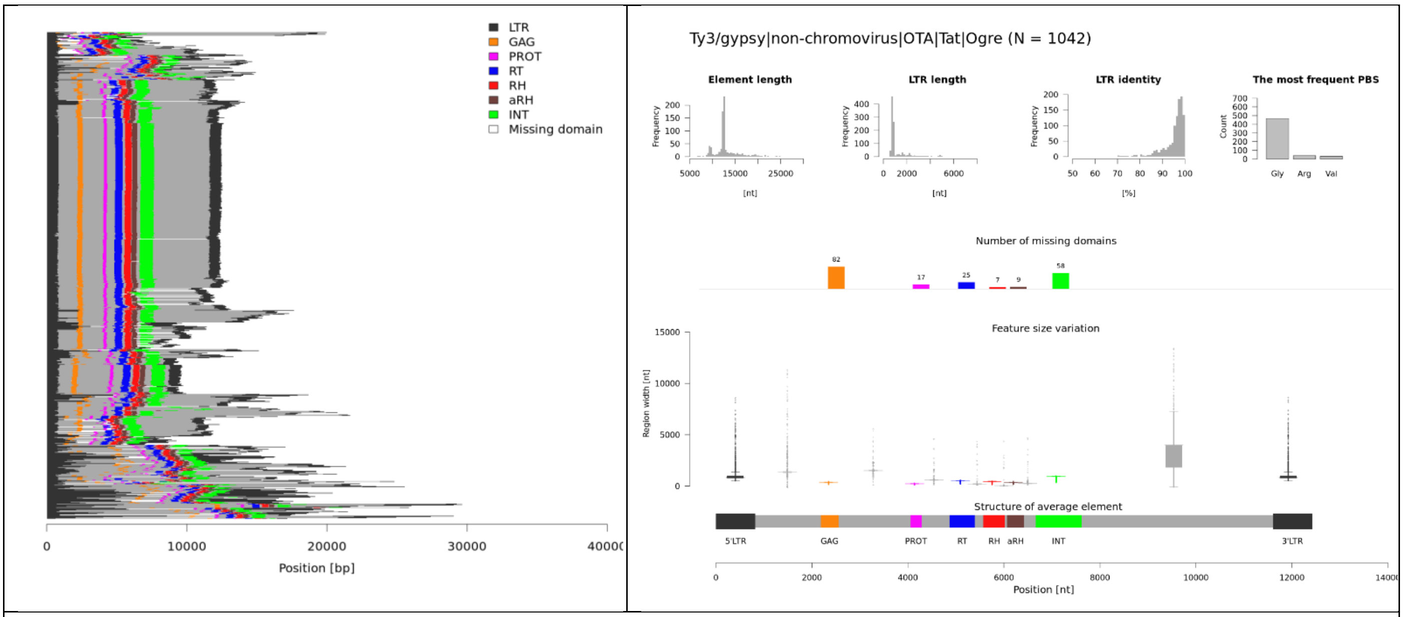

The left panel shows the structural organization of Ty3/gypsy-Ogre elements, whereas the right panel presents the summary statistics and structural features of these elements.

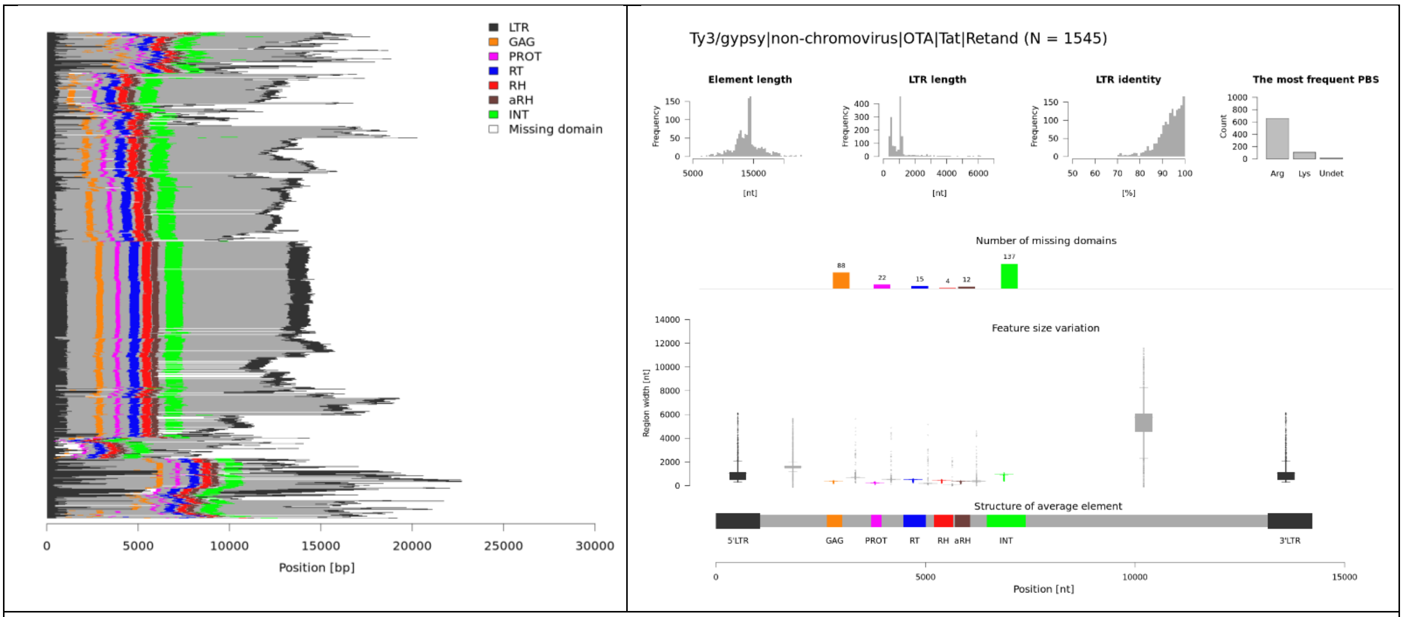

The left panel shows the structural organization of Ty3/gypsy-Retand elements, whereas the right panel presents the summary statistics and structural features of these elements.

***S. spontaneum* NP-X**

Number of elements of LTR-RT lineages in the complete genome of *Saccharum spontaneum* NP-X.

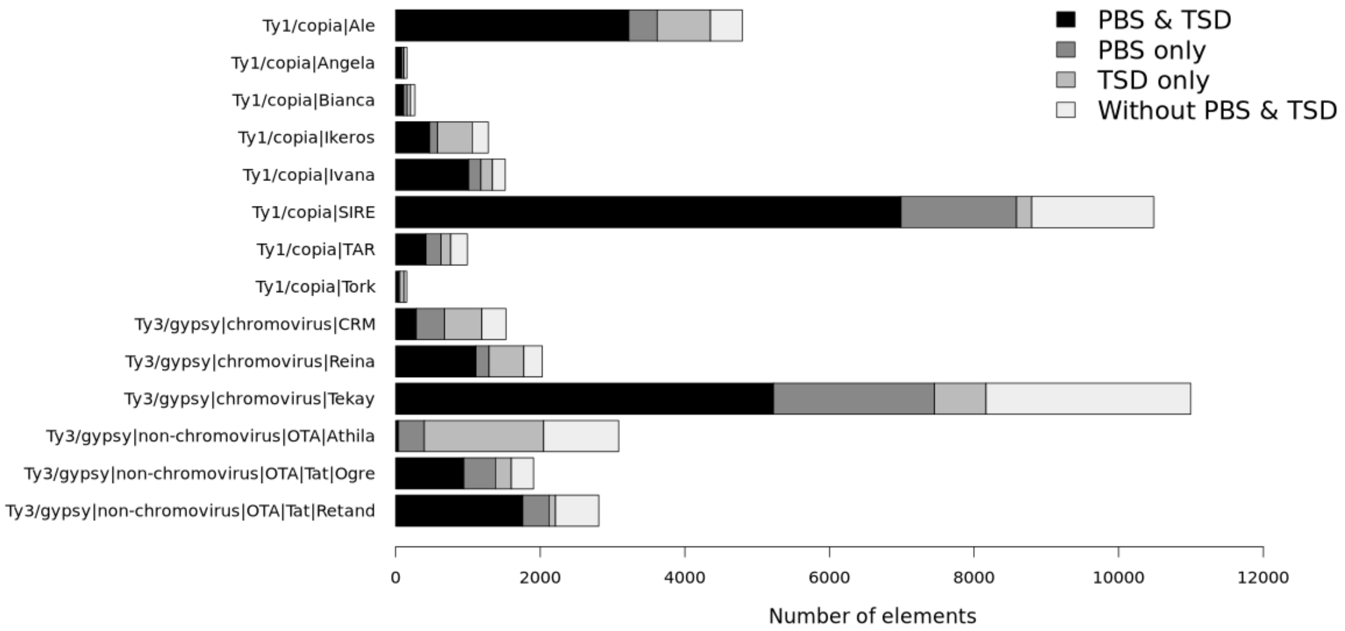

General description of the supplementary figures below:

Each supplementary figure consists of two panels. The left panel shows the structural organization of the LTR lineage identified using DANTE, where each horizontal line represents an individual element aligned according to its length. Colored segments indicate conserved protein domains, including long terminal repeats (LTRs, black), GAG (orange), protease (PROT, pink), integrase (INT, green), reverse transcriptase (RT, blue), and RNase H (RH, red), while gray regions correspond to sequences lacking detectable domains. Most elements exhibit the canonical LTR retrotransposon structure (LTR–GAG–PROT–INT–RT–RH–LTR), although variation in length and domain composition is evident, reflecting structural diversity and different levels of element degradation.

The right panel presents summary statistics and structural features of the same elements. The upper panels show the distribution of element length, LTR length, and LTR identity, as well as the frequency of the most common primer binding sites (PBS). The middle panel displays the number of elements with missing domains, highlighting differential conservation among domains. The lower panel illustrates variation in domain size across elements and the consensus structure of the average element, including LTRs, GAG, PROT, INT, RT, and RH domains. Overall, the elements display a conserved canonical organization, with high LTR identity suggesting recent insertion events and notable variability in domain composition and size.

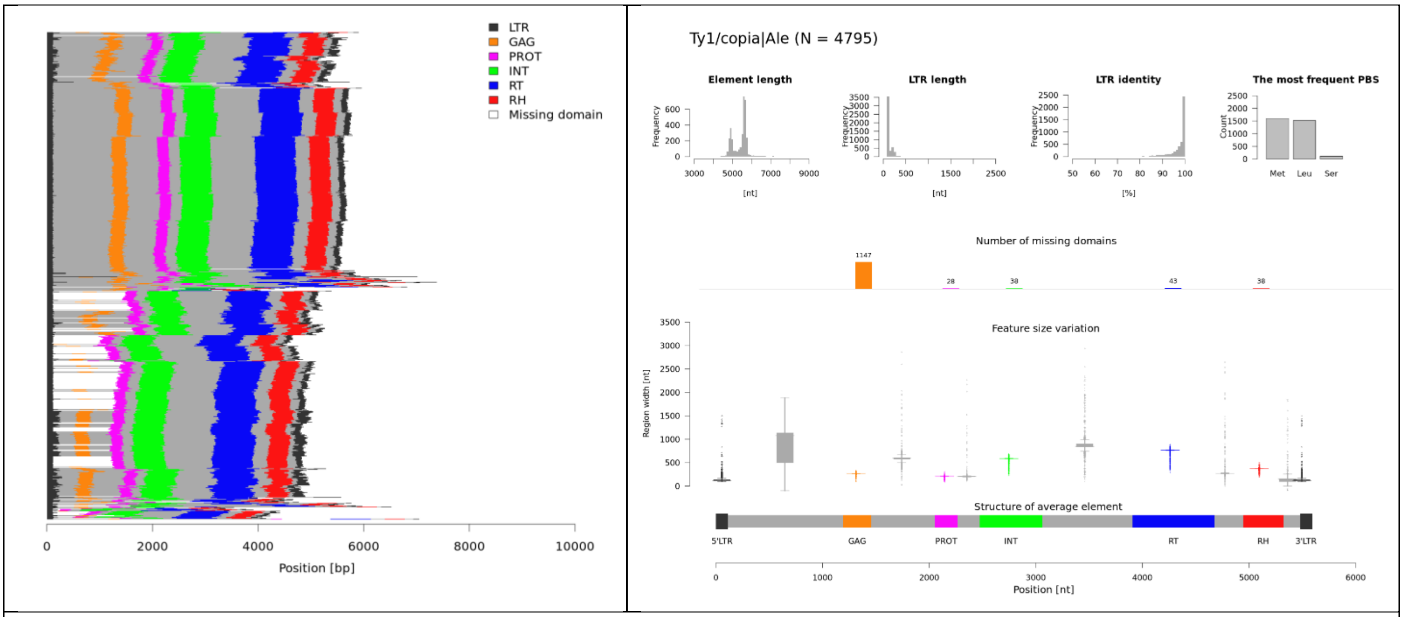

The left panel shows the structural organization of Ty1/copia-Ale elements, whereas the right panel presents the summary statistics and structural features of these elements.

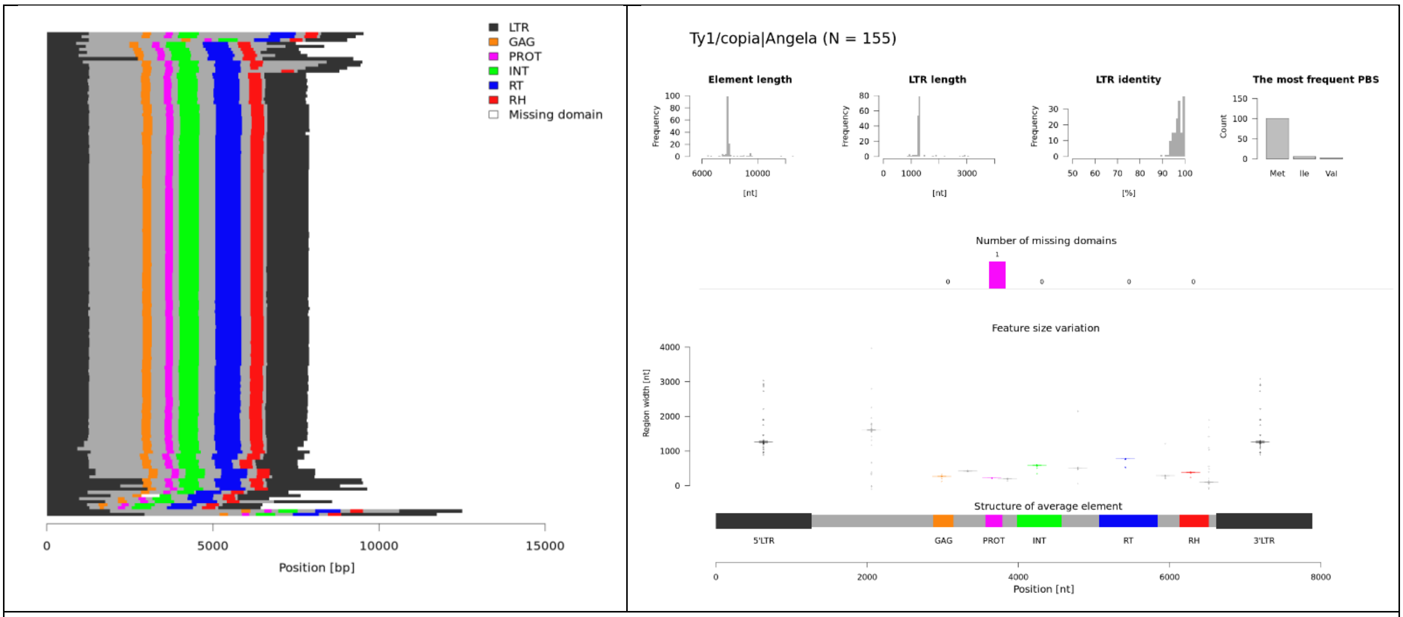

The left panel shows the structural organization of Ty1/copia-Angela elements, whereas the right panel presents the summary statistics and structural features of these elements.

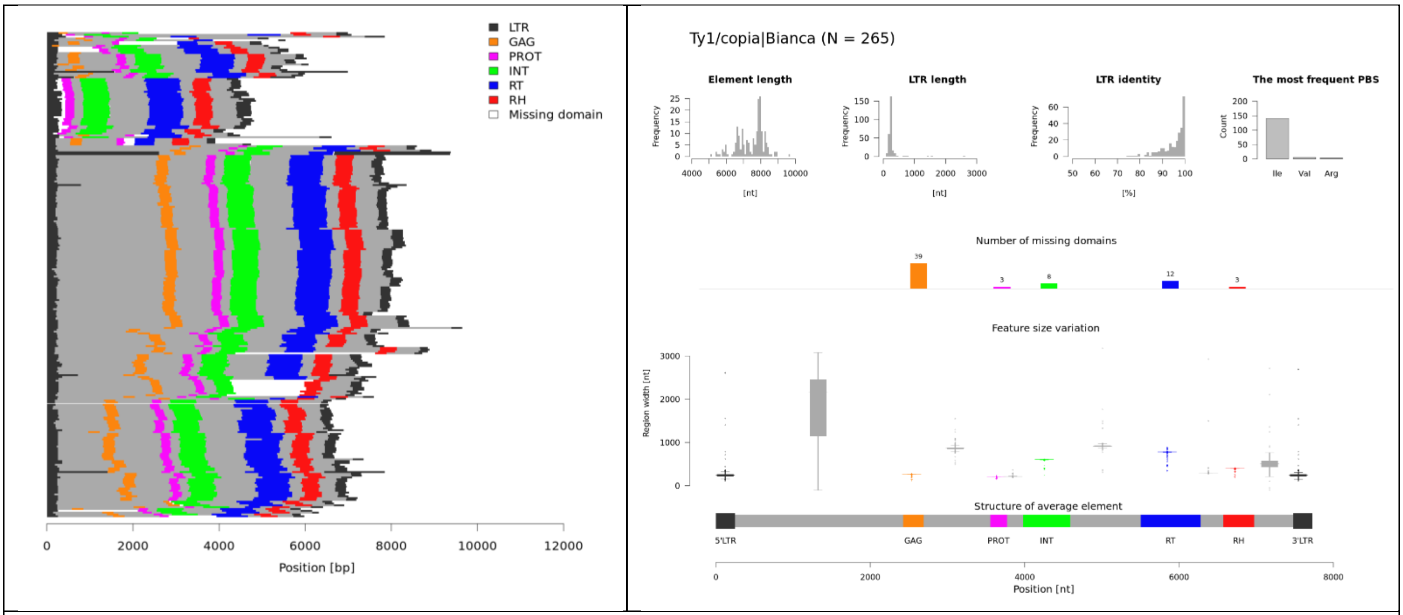

The left panel shows the structural organization of Ty1/copia-Bianca elements, whereas the right panel presents the summary statistics and structural features of these elements.

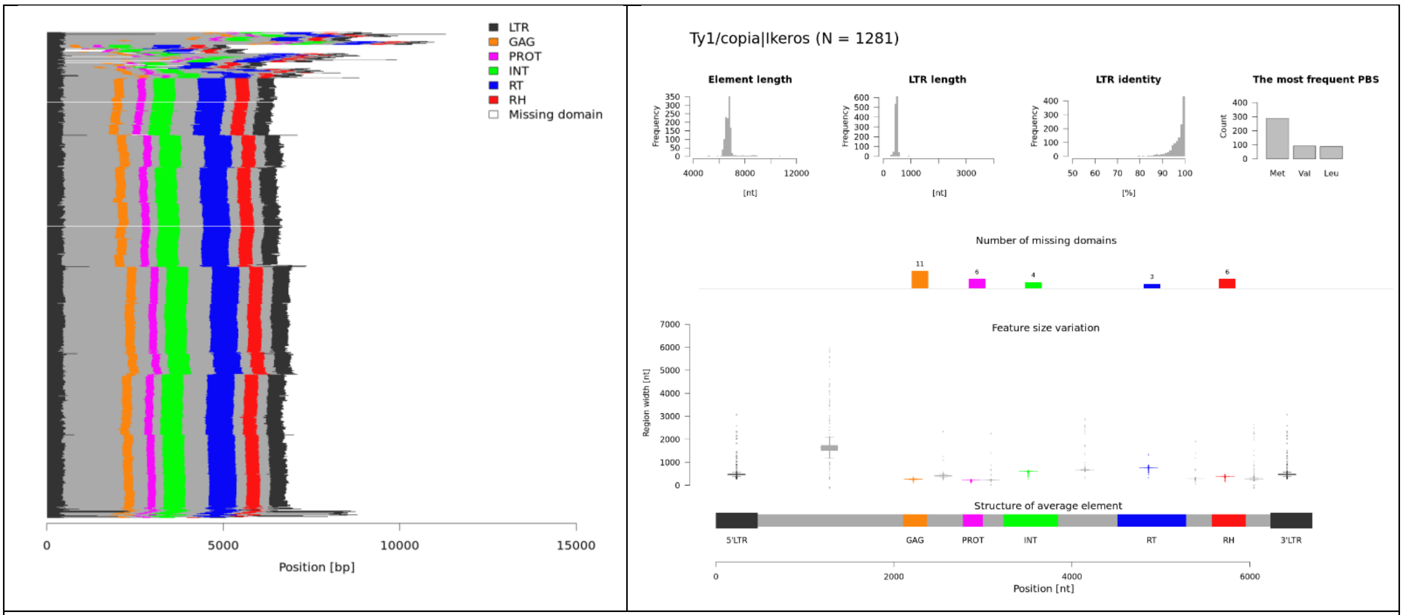

The left panel shows the structural organization of Ty1/copia-Ikeros elements, whereas the right panel presents the summary statistics and structural features of these elements.

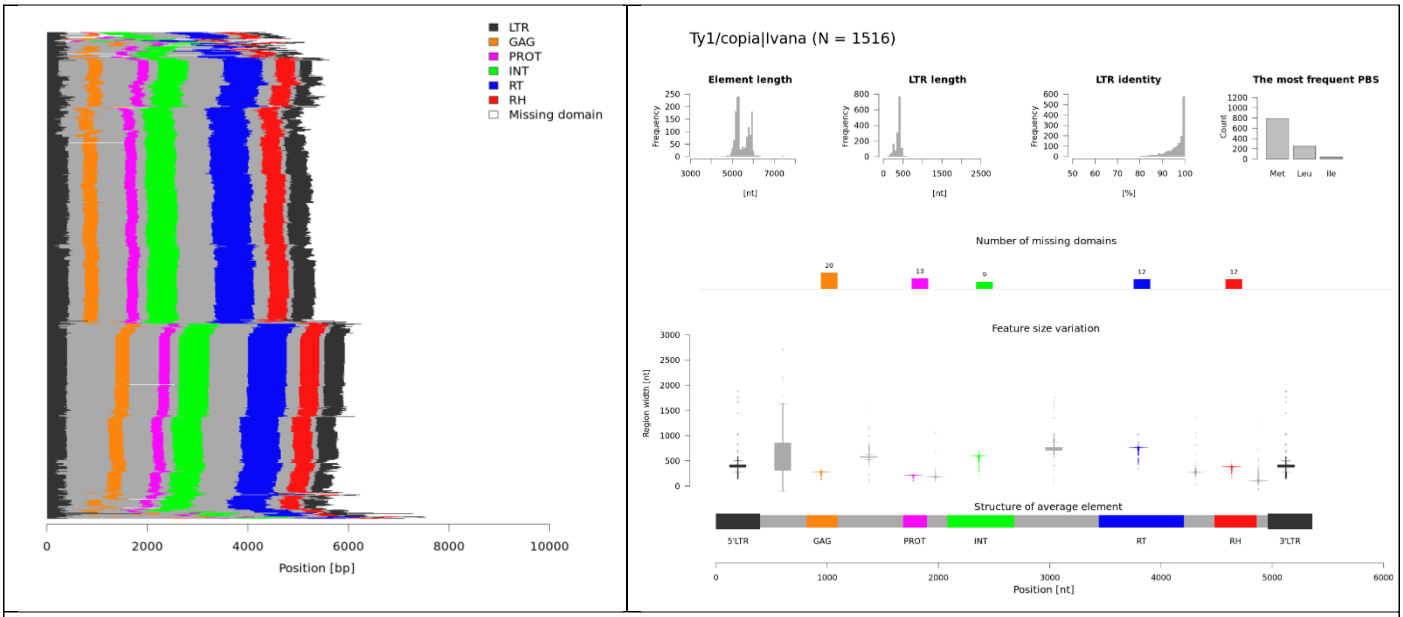

The left panel shows the structural organization of Ty1/copia-Ivana elements, whereas the right panel presents the summary statistics and structural features of these elements.

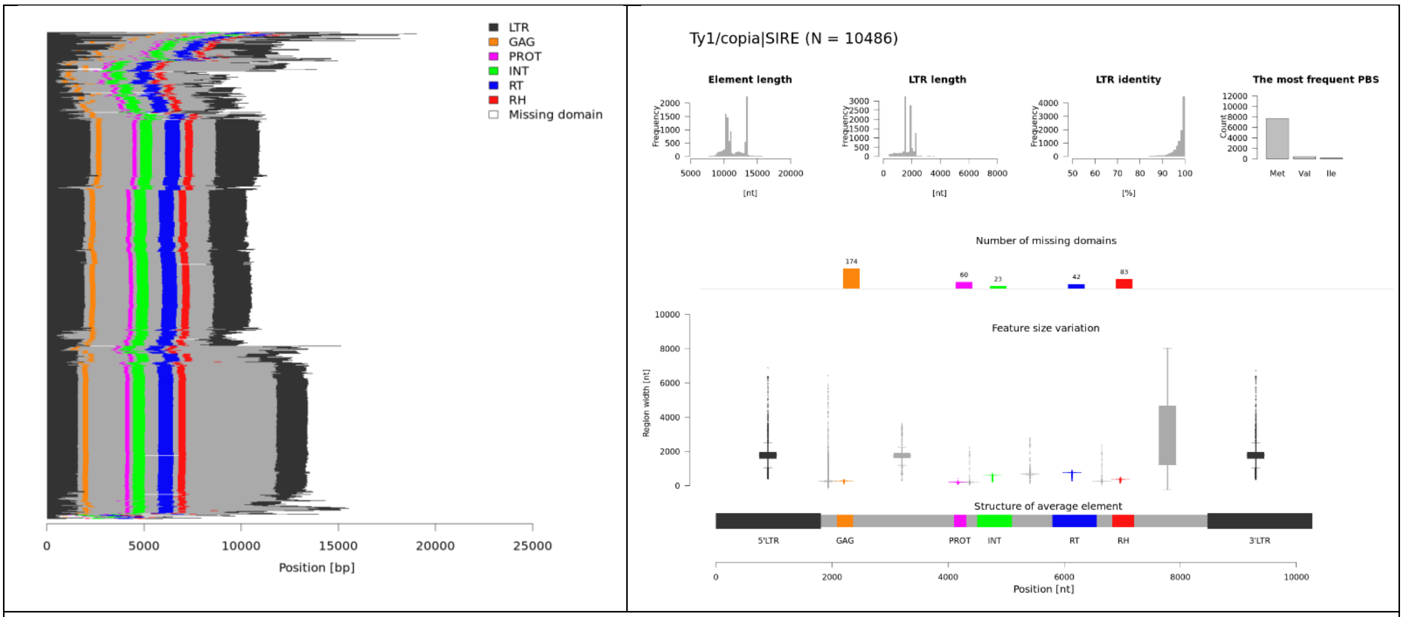

The left panel shows the structural organization of Ty1/copia-Sire elements, whereas the right panel presents the summary statistics and structural features of these elements.

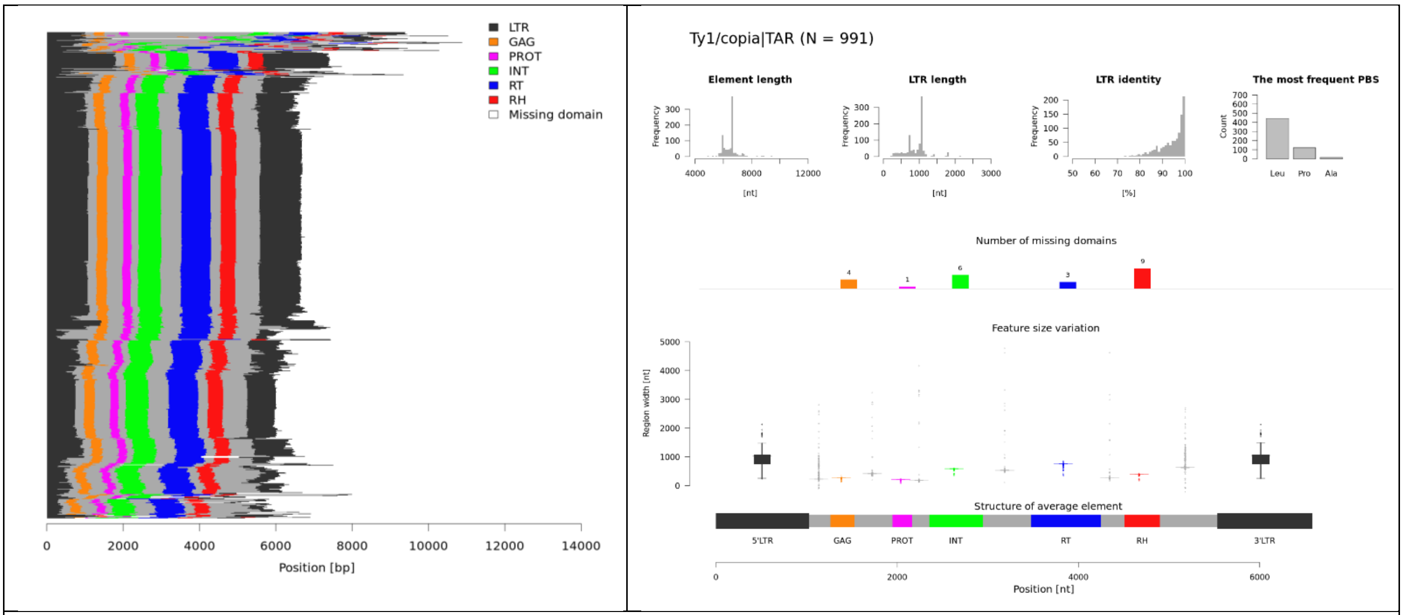

The left panel shows the structural organization of Ty1/copia-TAR elements, whereas the right panel presents the summary statistics and structural features of these elements.

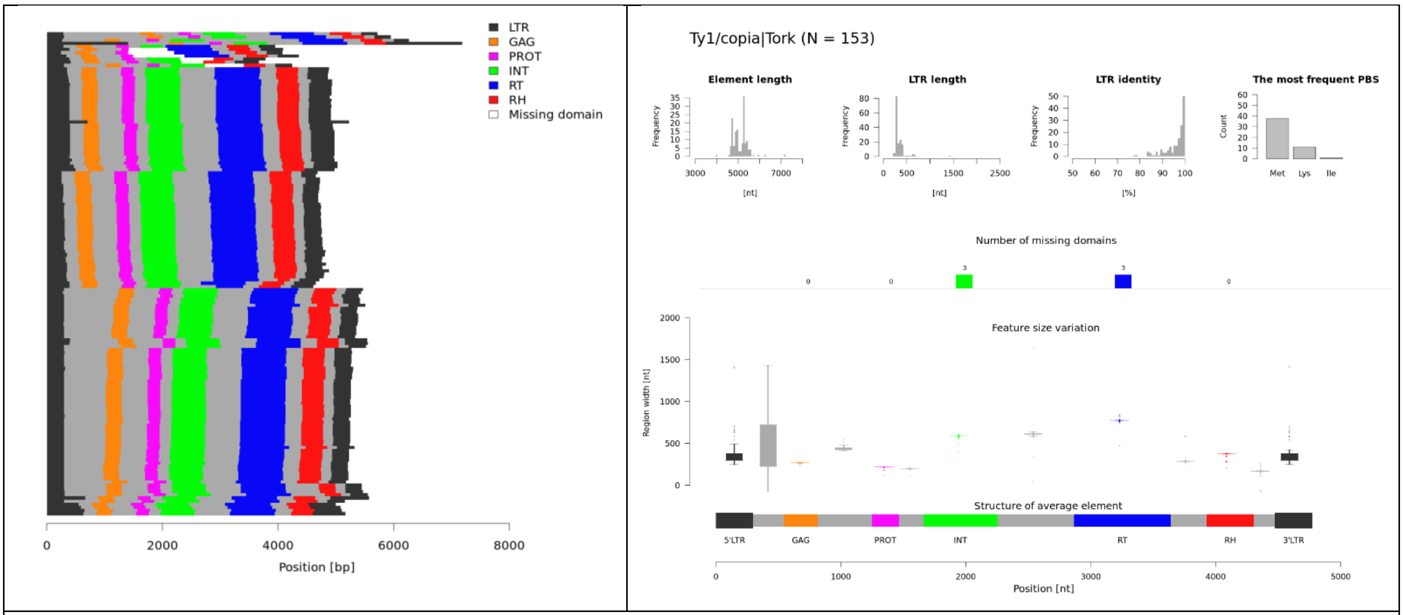

The left panel shows the structural organization of Ty1/copia-Tork elements, whereas the right panel presents the summary statistics and structural features of these elements.

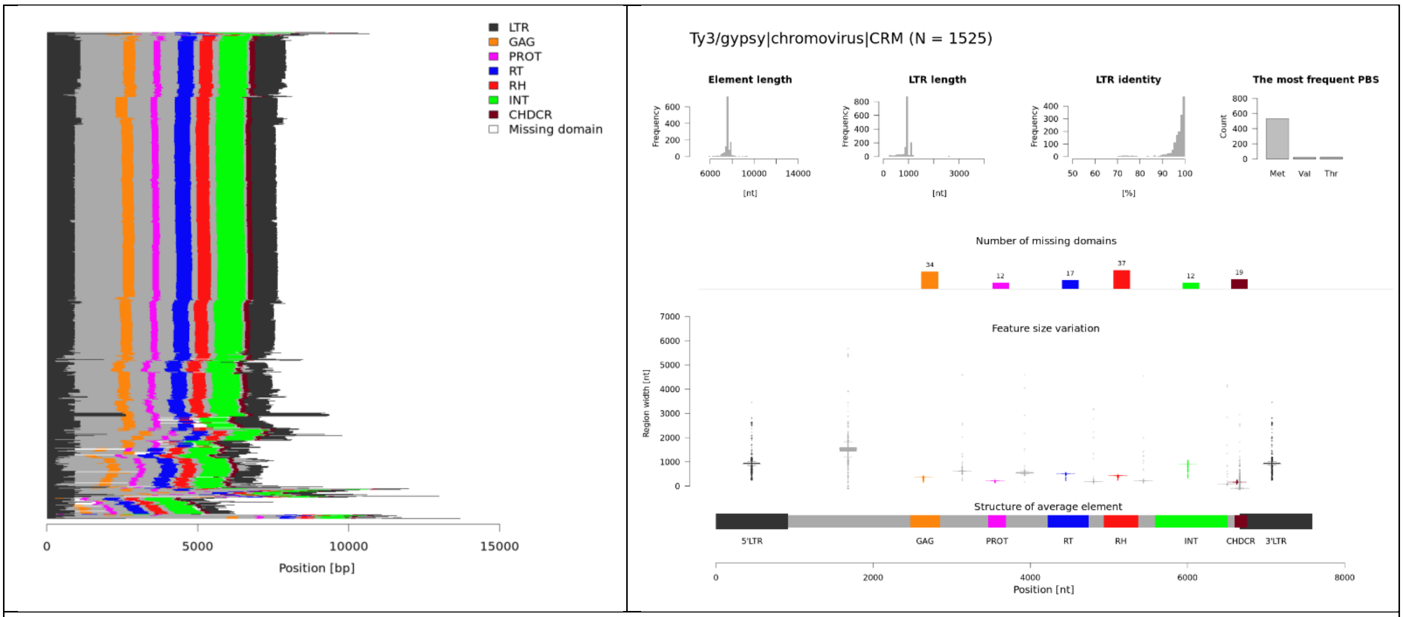

The left panel shows the structural organization of Ty3/gypsy-CRM elements, whereas the right panel presents the summary statistics and structural features of these elements.

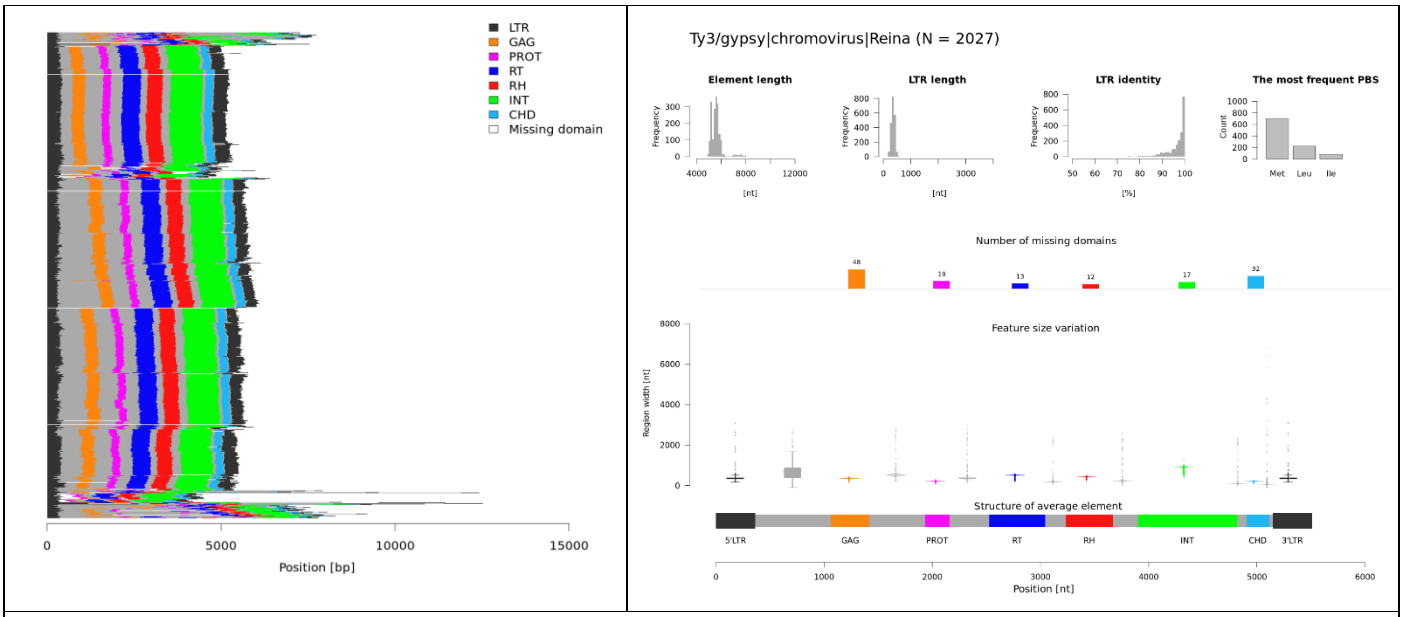

The left panel shows the structural organization of Ty3/gypsy-Reina elements, whereas the right panel presents the summary statistics and structural features of these elements.

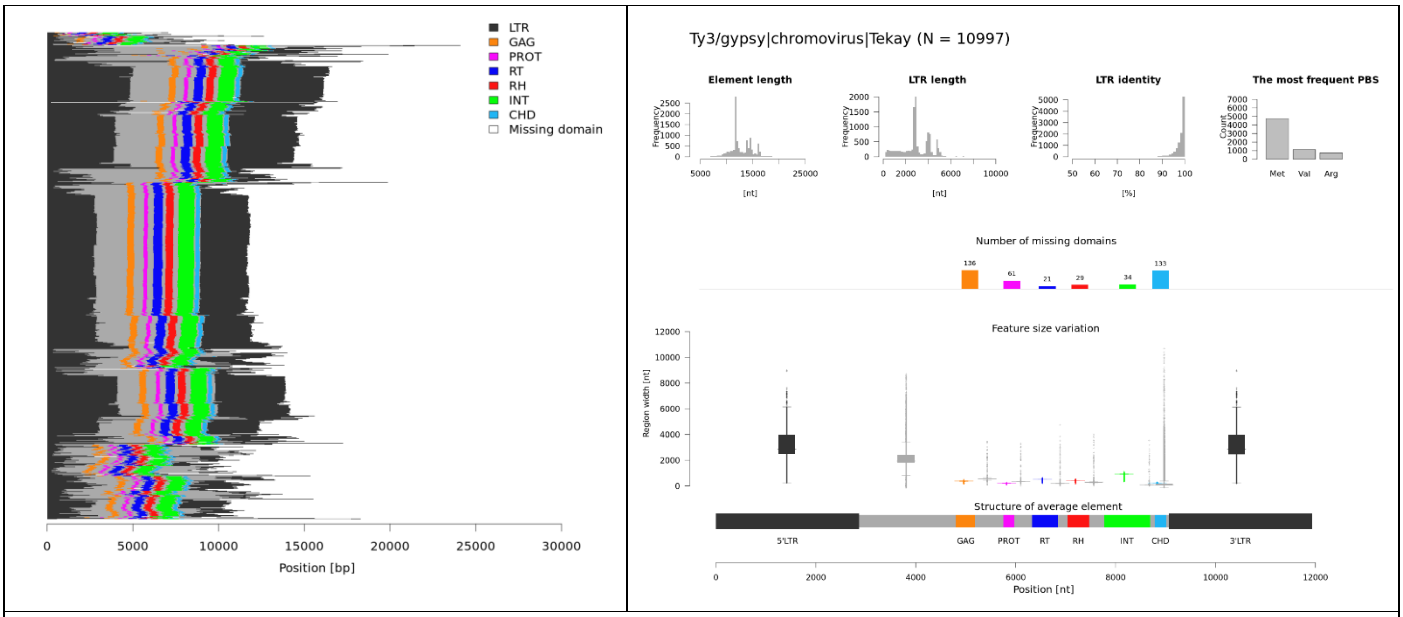

The left panel shows the structural organization of Ty3/gypsy-Tekay elements, whereas the right panel presents the summary statistics and structural features of these elements.

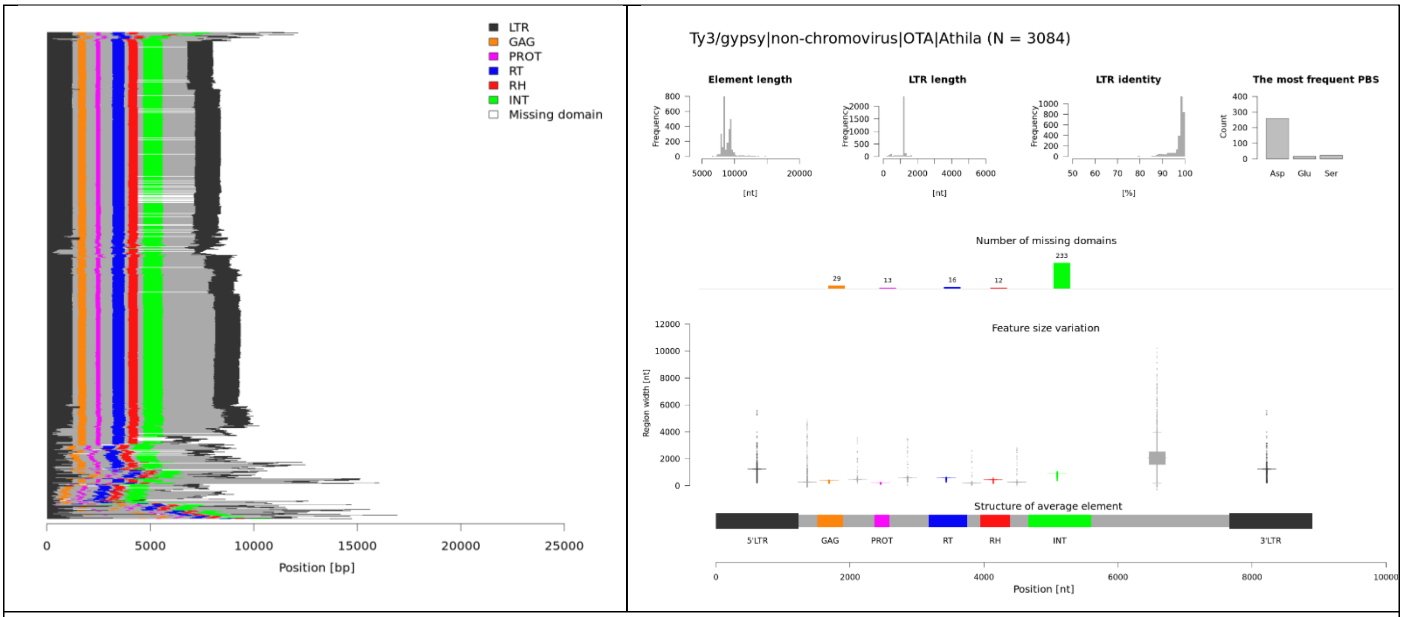

The left panel shows the structural organization of Ty3/gypsy-Athila elements, whereas the right panel presents the summary statistics and structural features of these elements.

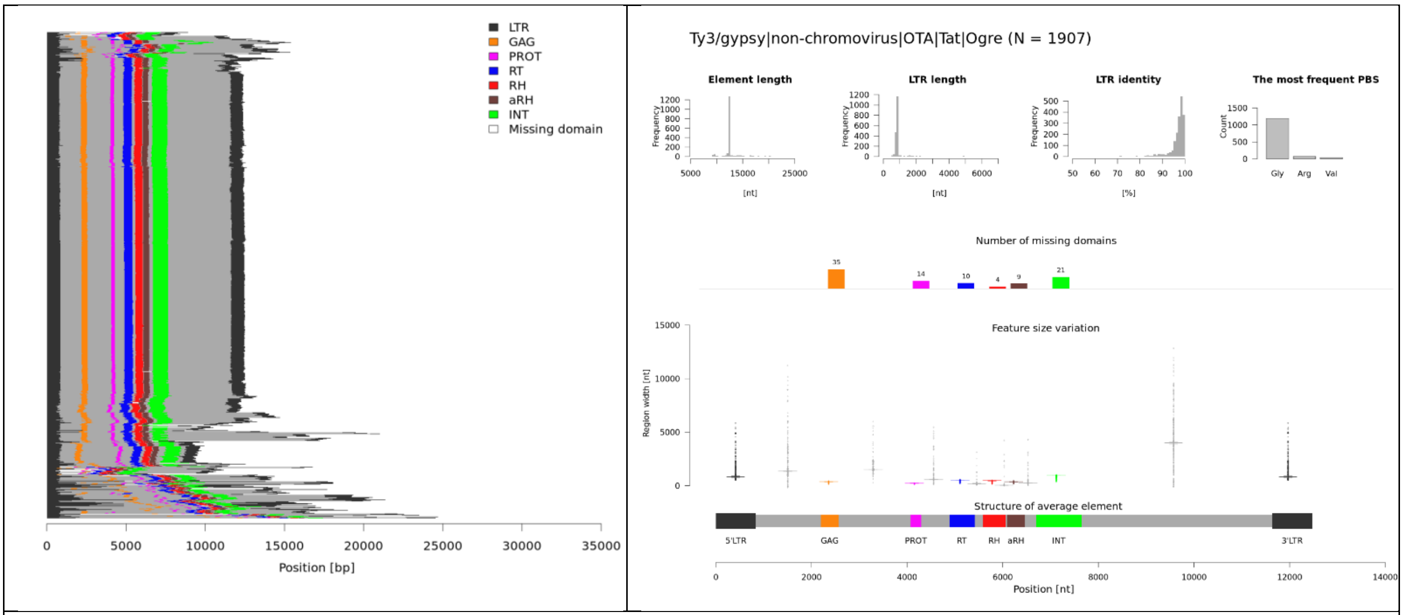

The left panel shows the structural organization of Ty3/gypsy-Ogre elements, whereas the right panel presents the summary statistics and structural features of these elements.

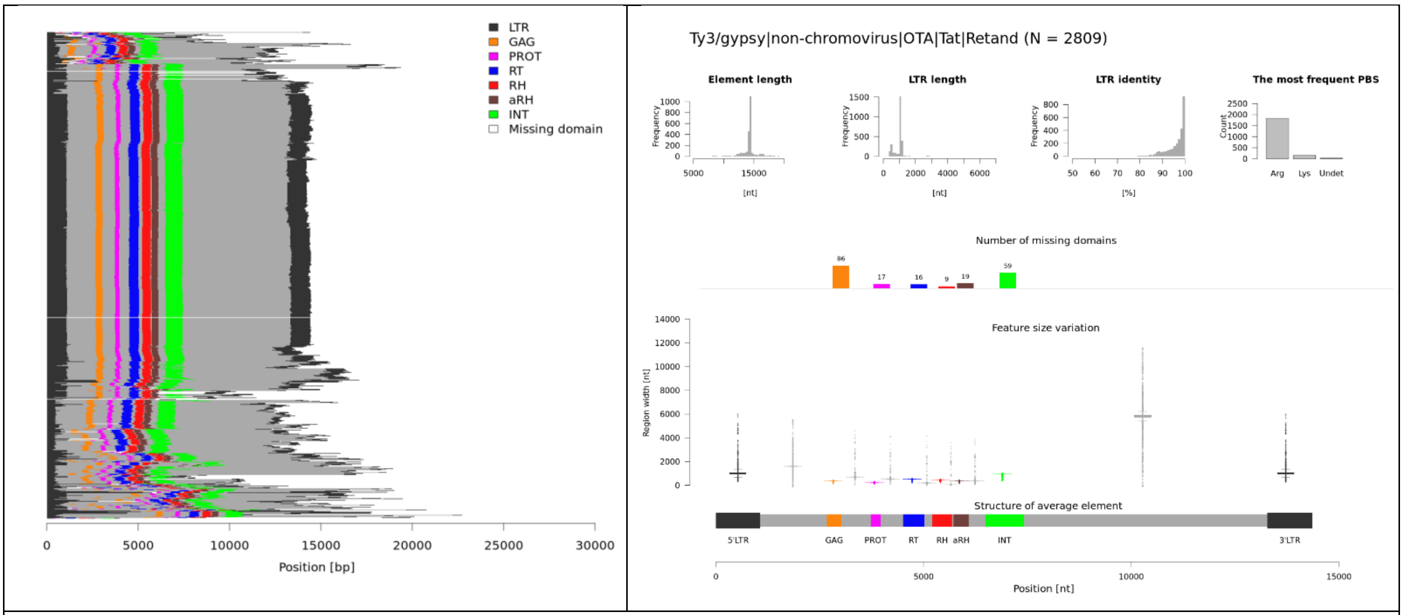

The left panel shows the structural organization of Ty3/gypsy-Retand elements, whereas the right panel presents the summary statistics and structural features of these elements.

cv. R570

Number of elements of LTR-RT lineages in the complete genome of *cv. R570*.

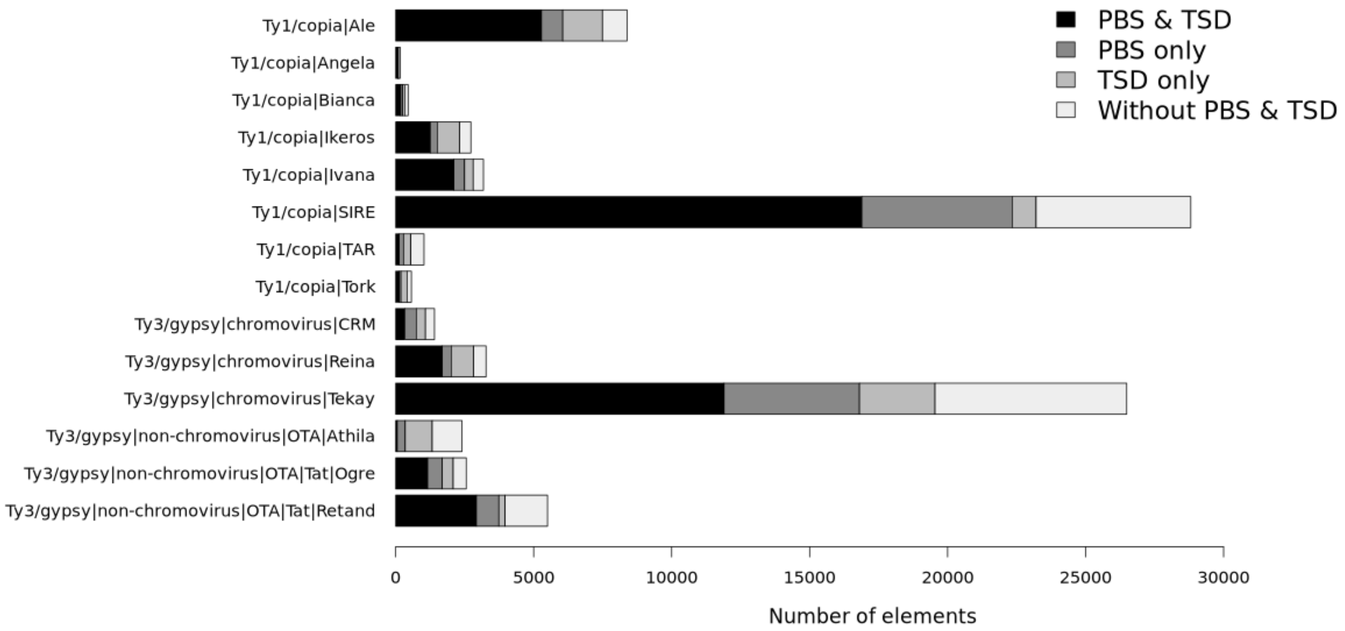

General description of the supplementary figures below:

Each supplementary figure consists of two panels. The left panel shows the structural organization of the LTR lineage identified using DANTE, where each horizontal line represents an individual element aligned according to its length. Colored segments indicate conserved protein domains, including long terminal repeats (LTRs, black), GAG (orange), protease (PROT, pink), integrase (INT, green), reverse transcriptase (RT, blue), and RNase H (RH, red), while gray regions correspond to sequences lacking detectable domains. Most elements exhibit the canonical LTR retrotransposon structure (LTR–GAG–PROT–INT–RT–RH–LTR), although variation in length and domain composition is evident, reflecting structural diversity and different levels of element degradation.

The right panel presents summary statistics and structural features of the same elements. The upper panels show the distribution of element length, LTR length, and LTR identity, as well as the frequency of the most common primer binding sites (PBS). The middle panel displays the number of elements with missing domains, highlighting differential conservation among domains. The lower panel illustrates variation in domain size across elements and the consensus structure of the average element, including LTRs, GAG, PROT, INT, RT, and RH domains. Overall, the elements display a conserved canonical organization, with high LTR identity suggesting recent insertion events and notable variability in domain composition and size.

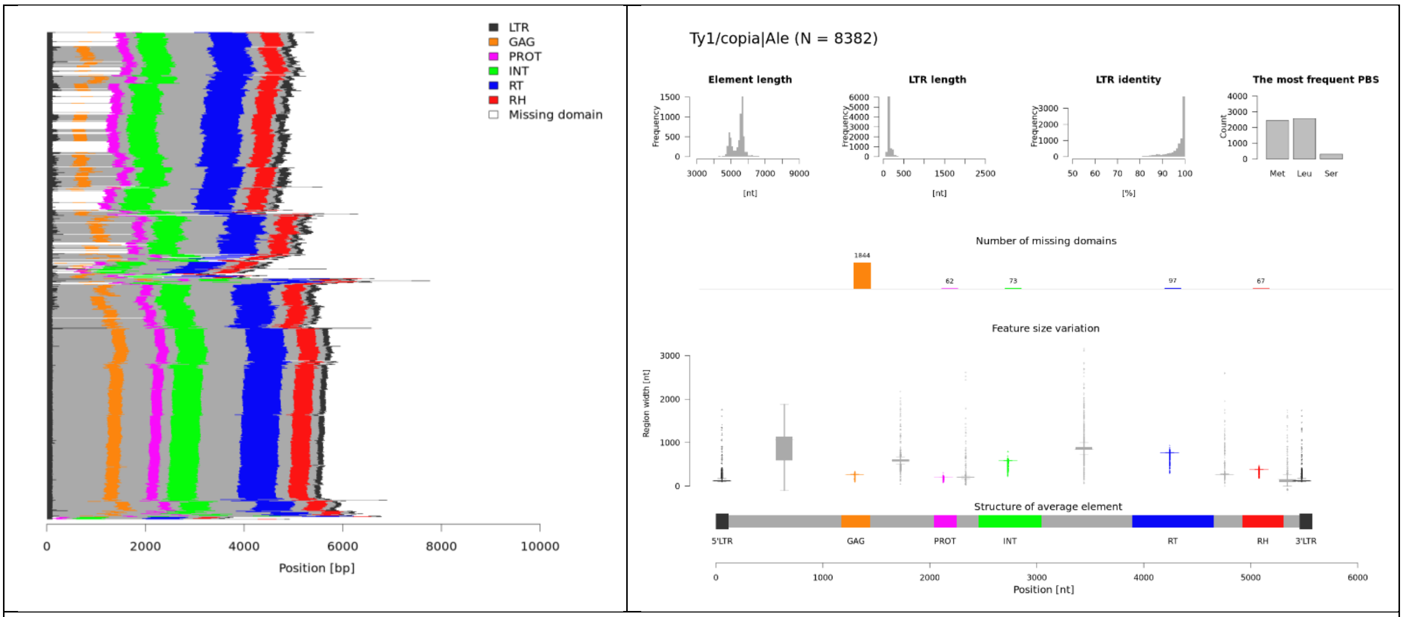

The left panel shows the structural organization of Ty1/copia-Ale elements, whereas the right panel presents the summary statistics and structural features of these elements.

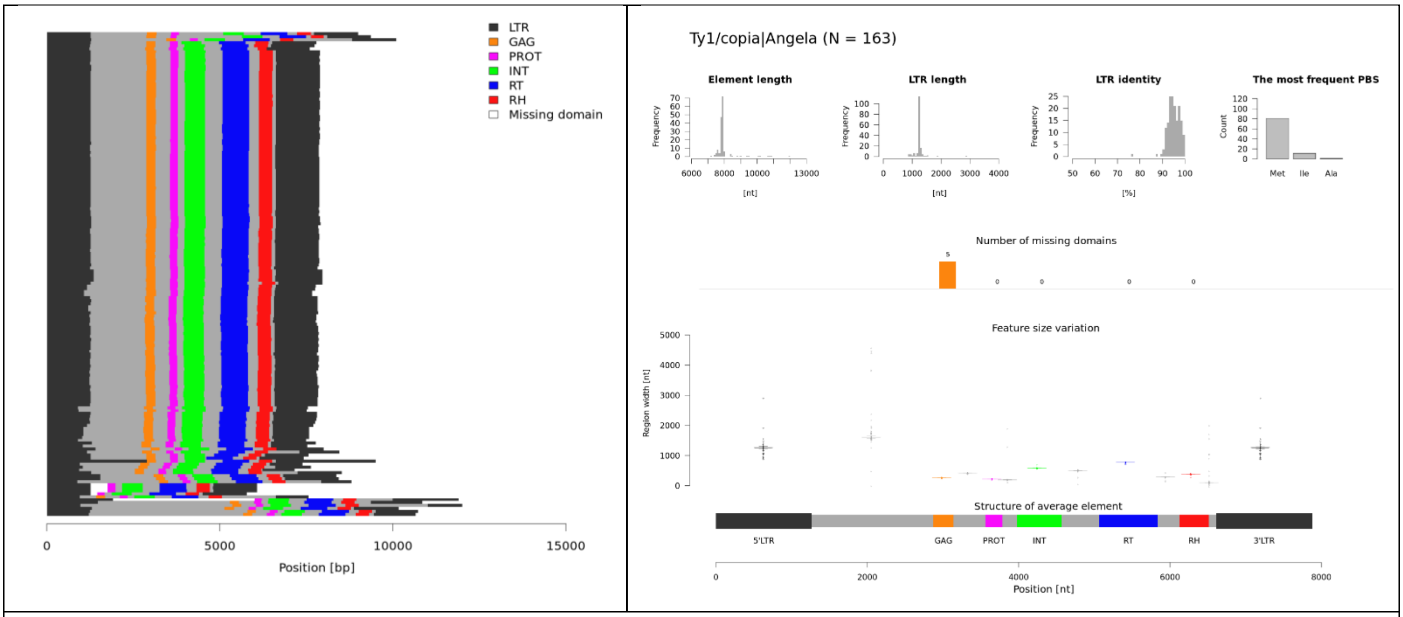

The left panel shows the structural organization of Ty1/copia-Angela elements, whereas the right panel presents the summary statistics and structural features of these elements.

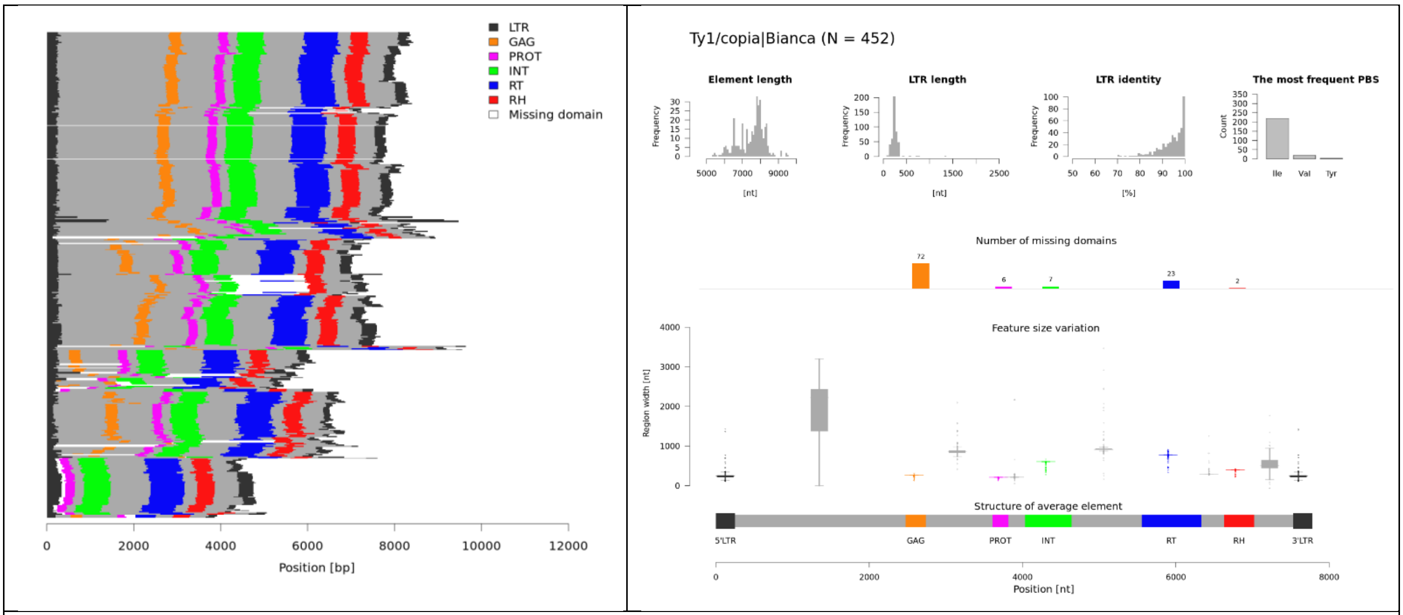

The left panel shows the structural organization of Ty1/copia-Bianca elements, whereas the right panel presents the summary statistics and structural features of these elements.

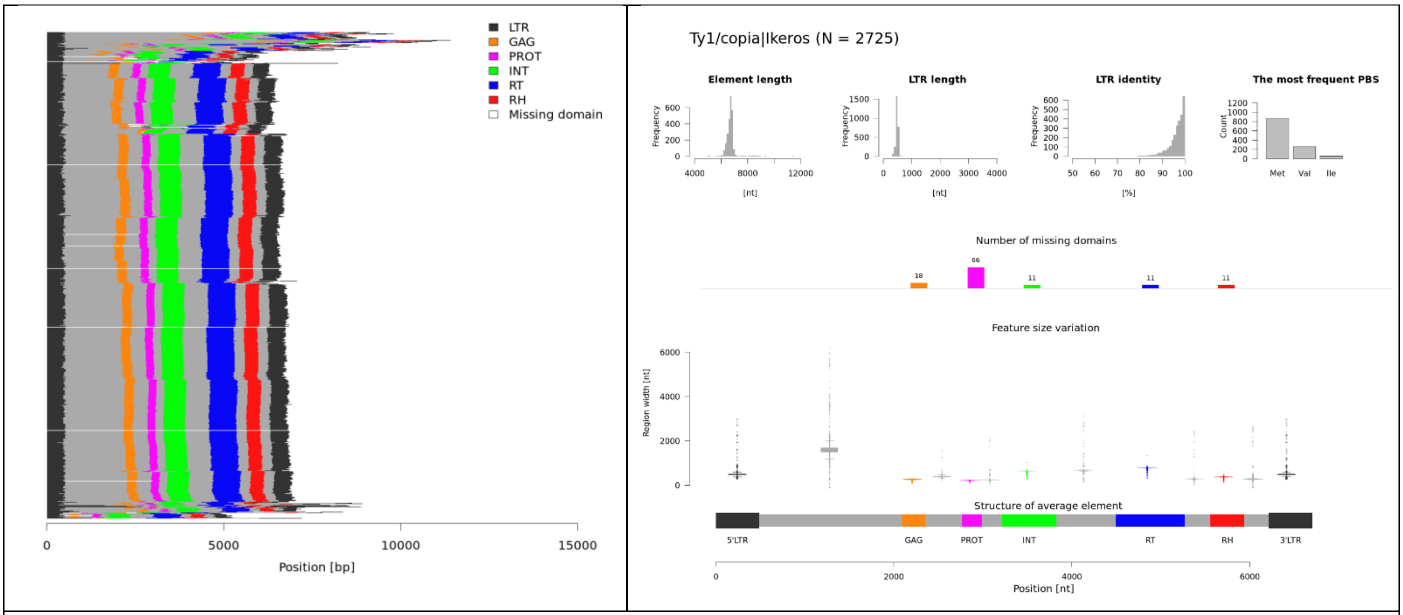

The left panel shows the structural organization of Ty1/copia-Ikeros elements, whereas the right panel presents the summary statistics and structural features of these elements.

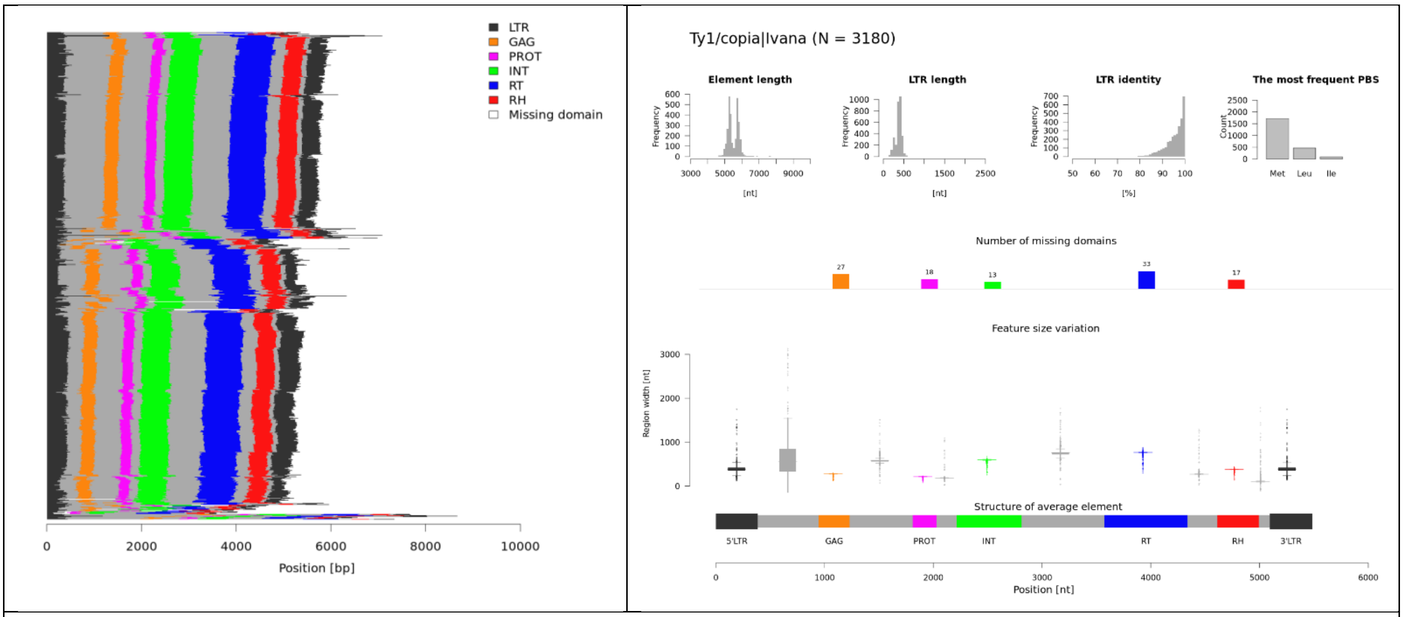

The left panel shows the structural organization of Ty1/copia-Ivana elements, whereas the right panel presents the summary statistics and structural features of these elements.

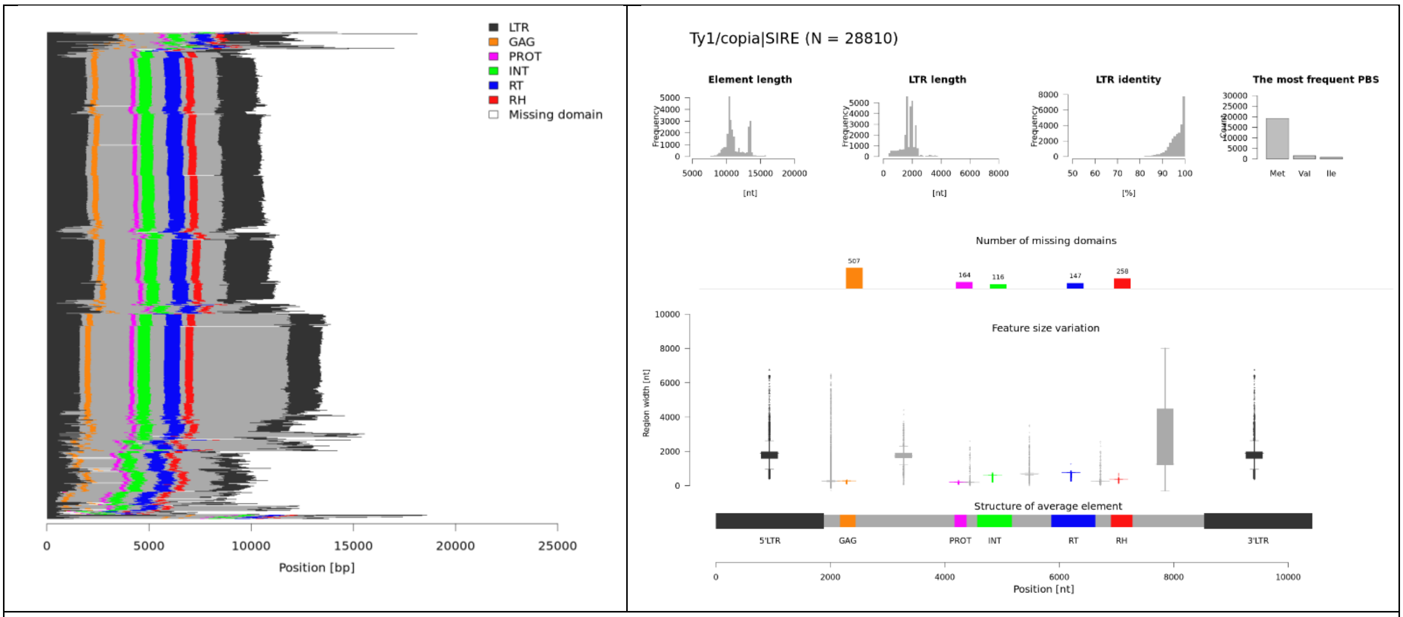

The left panel shows the structural organization of Ty1/copia-Sire elements, whereas the right panel presents the summary statistics and structural features of these elements.

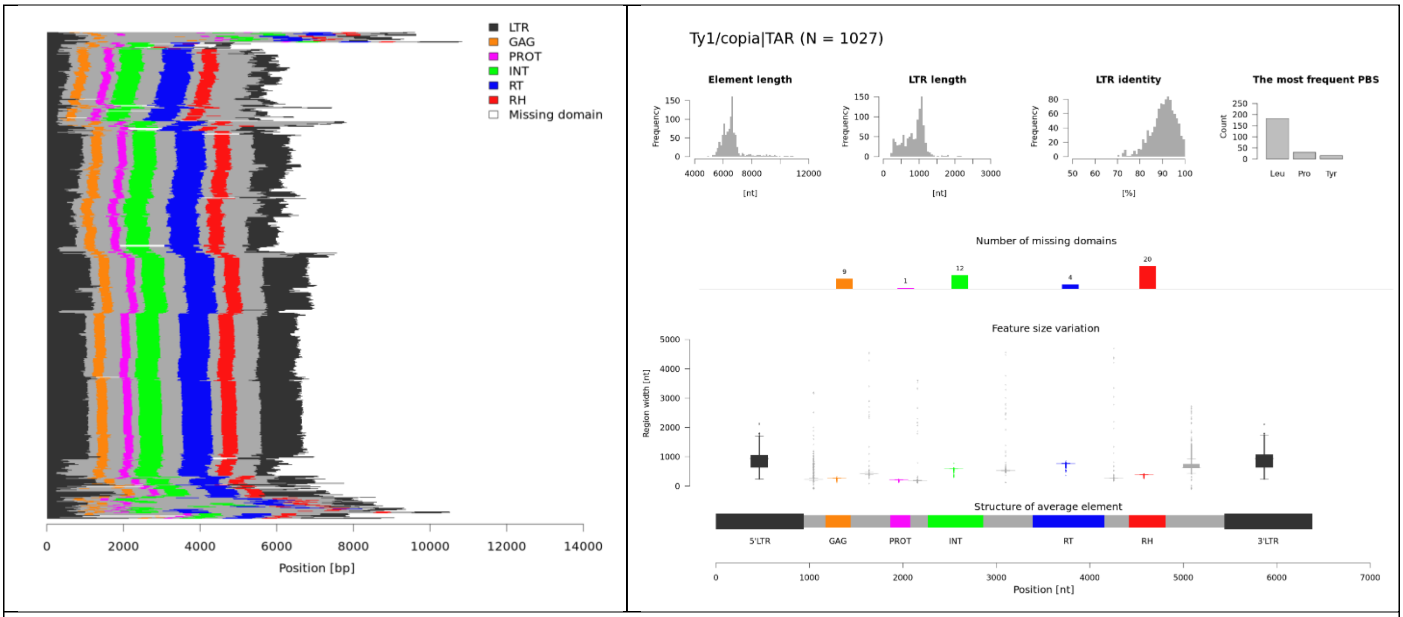

The left panel shows the structural organization of Ty1/copia-TAR elements, whereas the right panel presents the summary statistics and structural features of these elements.

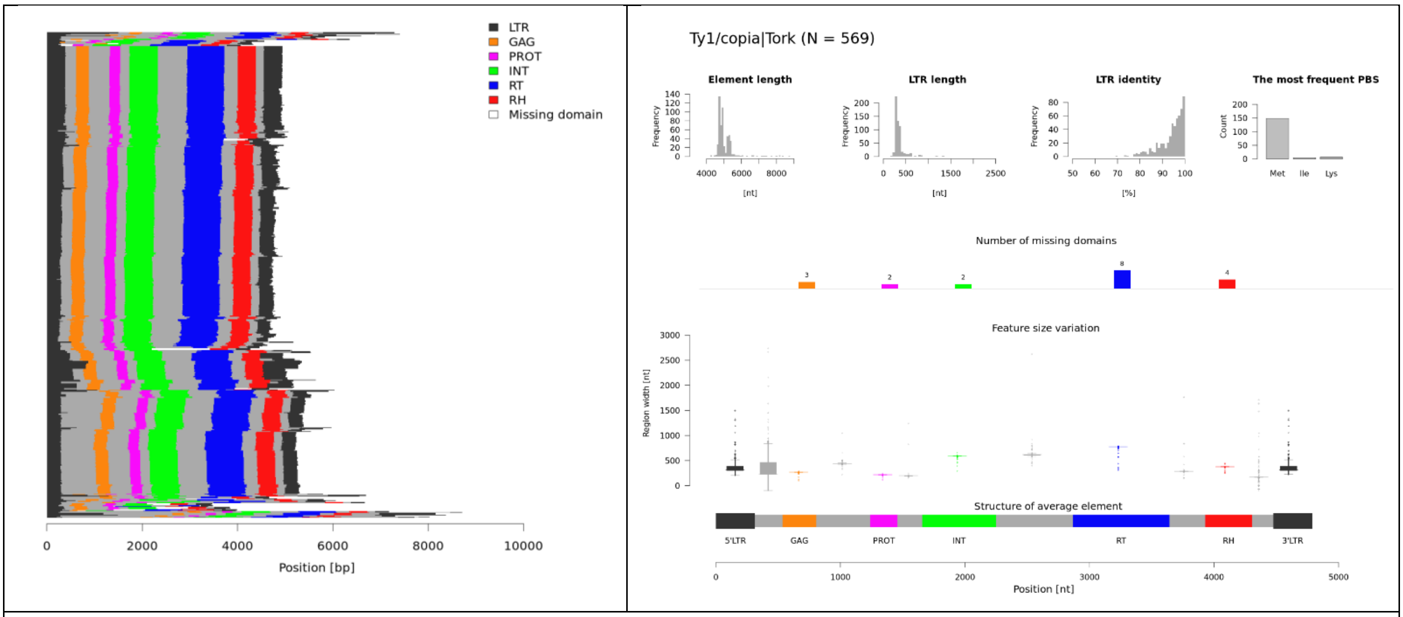

The left panel shows the structural organization of Ty1/copia-Tork elements, whereas the right panel presents the summary statistics and structural features of these elements.

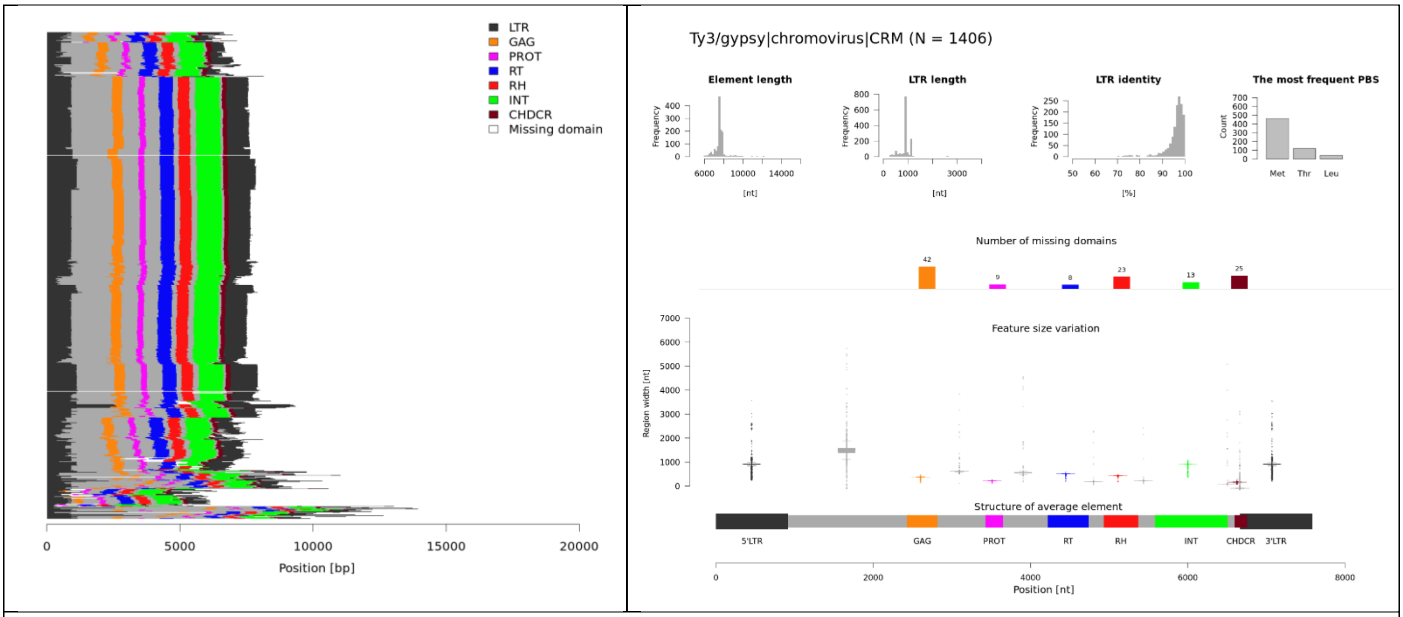

The left panel shows the structural organization of Ty3/gypsy-CRM elements, whereas the right panel presents the summary statistics and structural features of these elements.

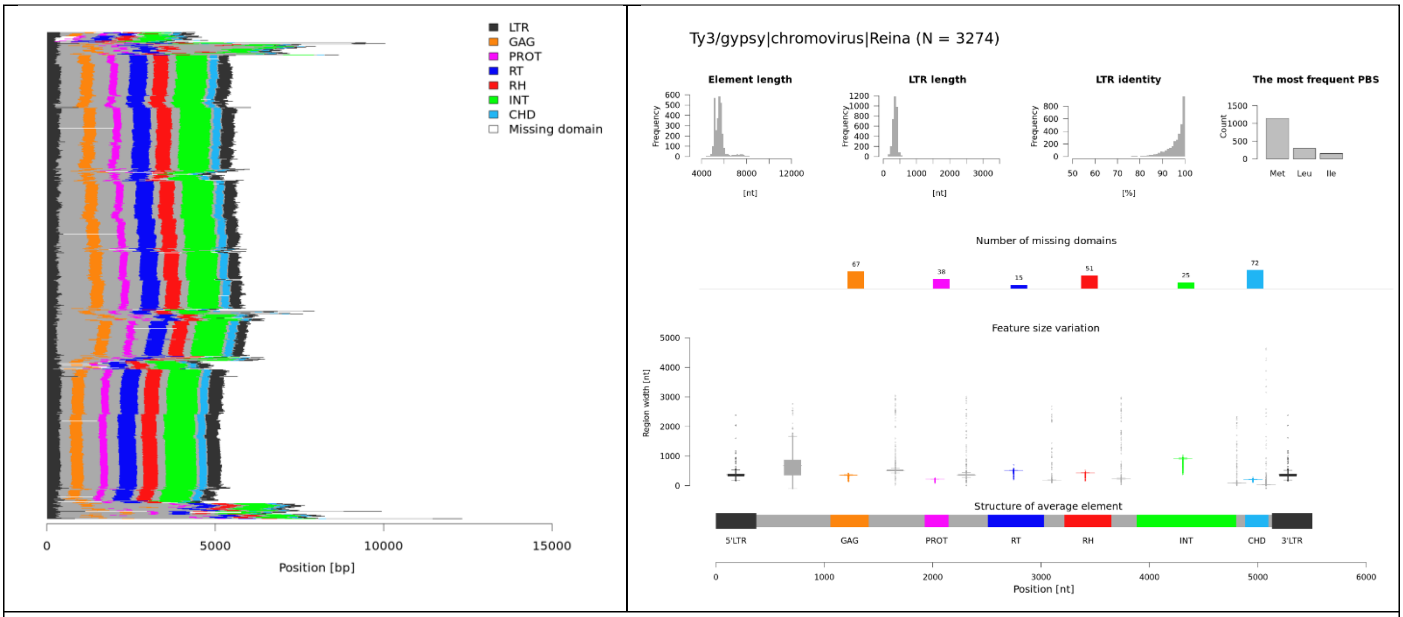

The left panel shows the structural organization of Ty3/gypsy-Reina elements, whereas the right panel presents the summary statistics and structural features of these elements.

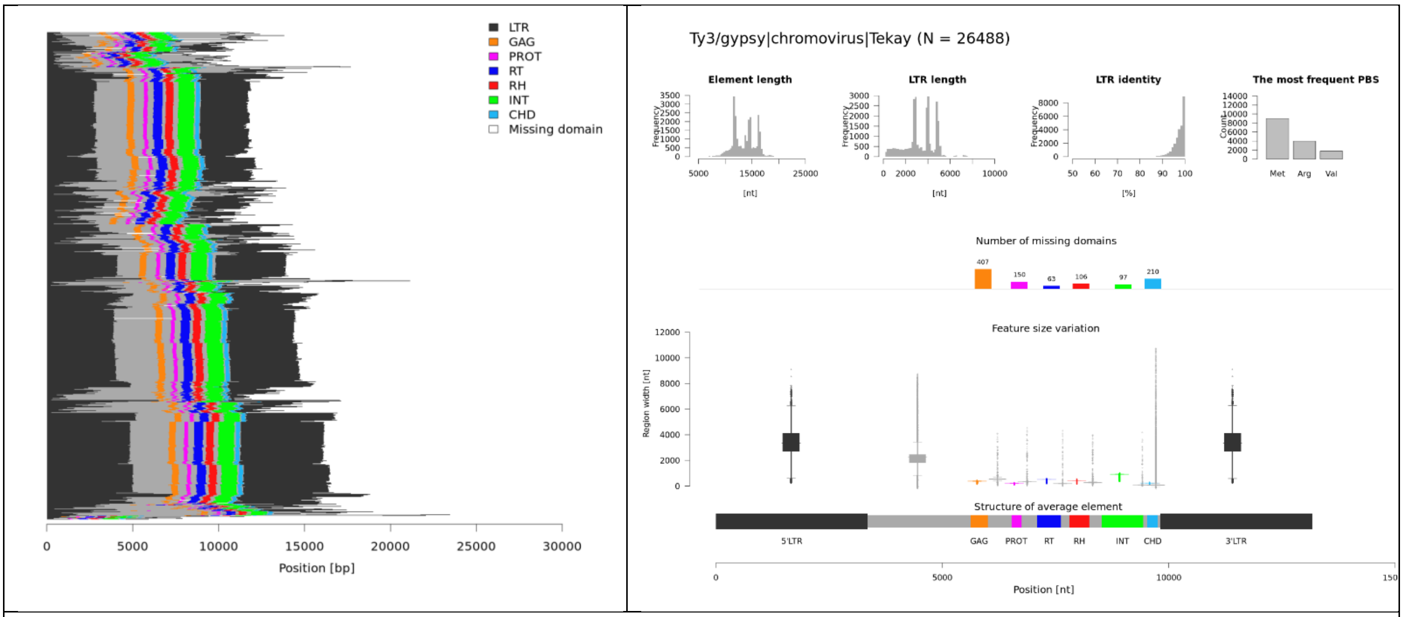

The left panel shows the structural organization of Ty3/gypsy-Tekay elements, whereas the right panel presents the summary statistics and structural features of these elements.

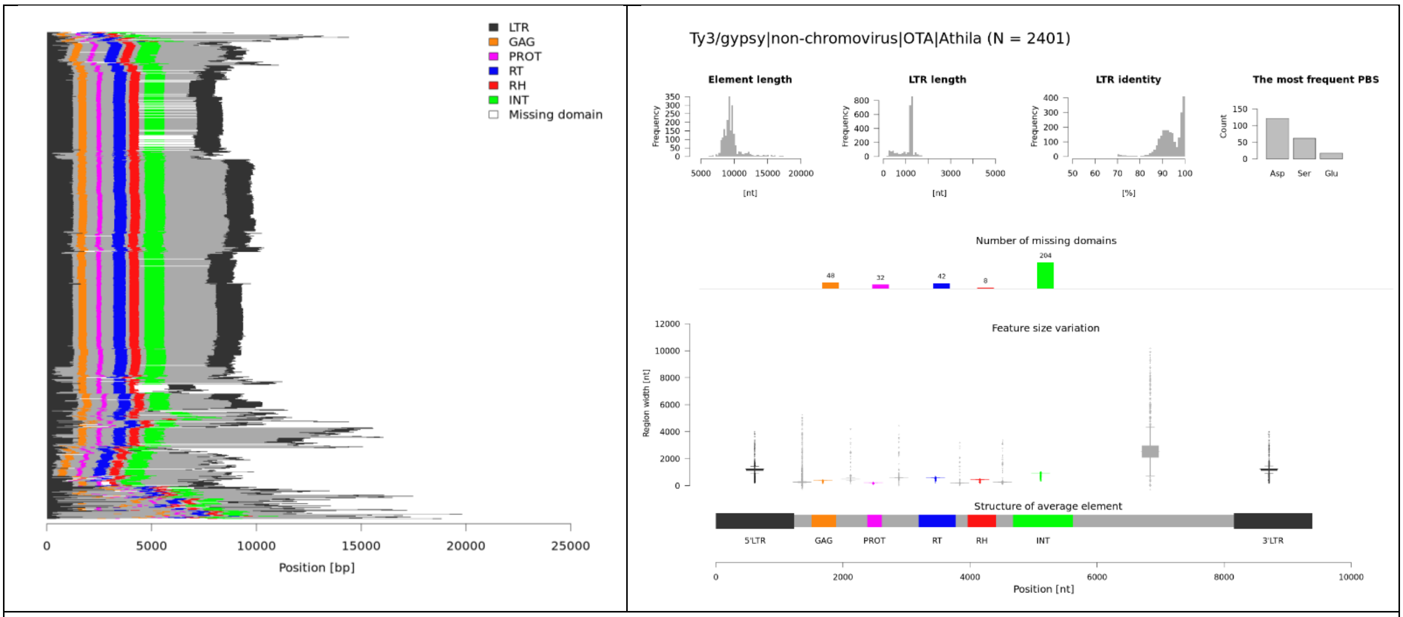

The left panel shows the structural organization of Ty3/gypsy-Athila elements, whereas the right panel presents the summary statistics and structural features of these elements.

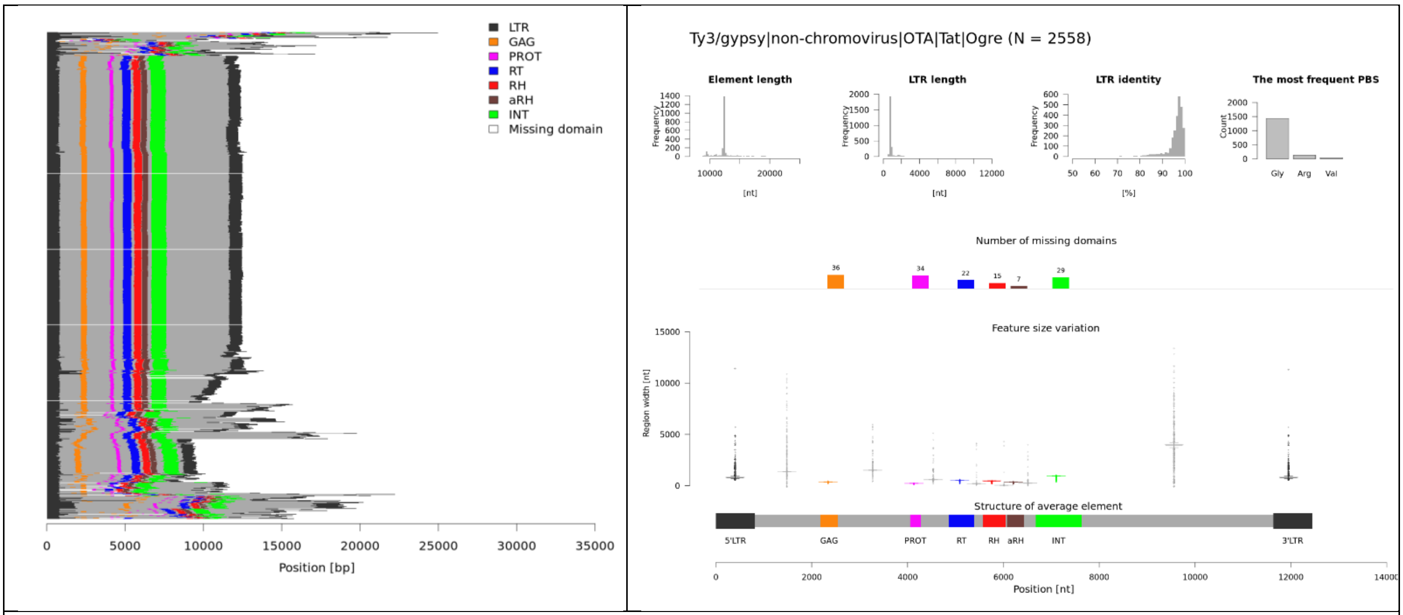

The left panel shows the structural organization of Ty3/gypsy-Ogre elements, whereas the right panel presents the summary statistics and structural features of these elements.

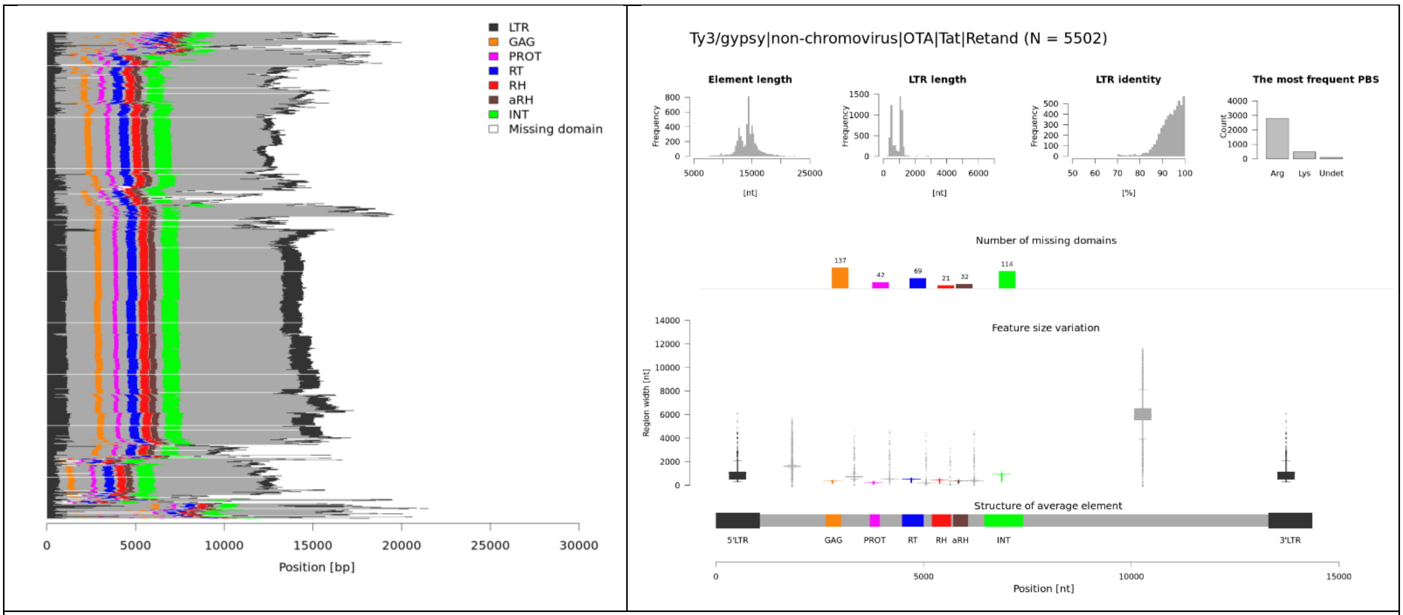

The left panel shows the structural organization of Ty3/gypsy-Retand elements, whereas the right panel presents the summary statistics and structural features of these elements.
