# Supplementary material for: The repeatome landscape in the “Saccharum complex”
Source: Front Plant Sci. 2026 May 21;17:1809735. doi: 10.3389/fpls.2026.1809735 (PMC13233458; doi:10.3389/fpls.2026.1809735)
Supplement: Supplementary file 3 [file Presentation2.pdf]

Supplementary file 1. Shared high confidence satellites among the "*Saccharum* complex"

Satellite DNA (satDNA) clusters were identified by TAREAN (Tandem Repeat Analyzer), included in RepeatExplorer2 (Novák et al., 2013, 2020). The consensus_dimer.fasta file, comprising reconstructed consensus dimers generated by the TAREAN, were retrieved, aligned with Geneious Prime v. 2022.2 (https://www.geneious.com). Heatmap representations of the similarity matrix were generated using package pheatmap (Kolde, 2019) in R v. 4.3.2 (R: The R Project for Statistical Computing, n.d.).

satDNA classification followed the criteria established by Ruiz-Ruano et al. (2016), utilizing specific sequence similarity thresholds: **1.** Monomeric sequences displaying less than **80%** similarity were assigned to different families within the same superfamily; **2.** Sequences sharing **80–95%** similarity were considered variants belonging to the same family; and **3.** Sequences exhibiting greater than **95%** similarity were classified as variants of the same consensus monomer. The satDNA families were designated using a four-letter species prefix (e.g., Soff for *S. officinarum*), followed by the descriptor 'Sat', a numerical identifier assigned by decreasing abundance, and the consensus monomer length. Names followed by an * indicate high confidence satDNA.

Alignment of all SatDNA sequences with identical consensus length and the corresponding heatmap generated from the similarity matrix. SatDNA names with a * correspond to high confidence SatDNA identified by TAREAN.

- Alignment of 4 sequences: SnarengSat06-20, SsinKacSat05-20*, Sspo517Sat07-20, SspoGlaSat08-20

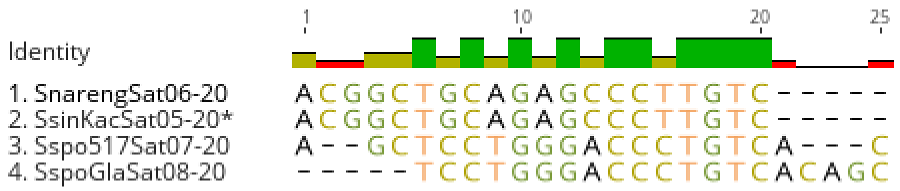

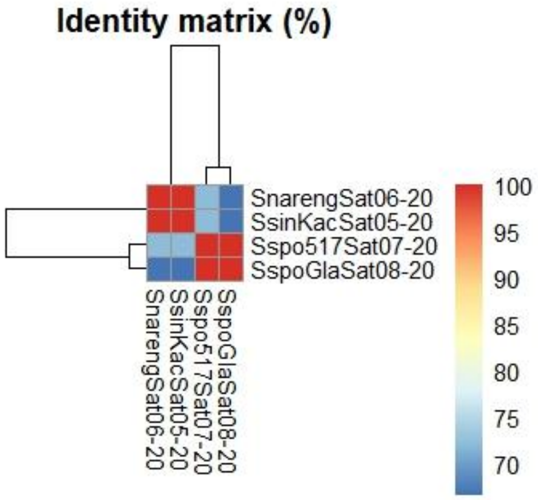

According to satDNA classification followed the criteria established by Ruiz-Ruano et al. (2016), SnarengSat06-20 and SsinKacSat05-20*; Sspo517Sat07-20 and SspoGlaSat08-20 are variants of the same consensus monomer.

- Alignment of 30 sequences: EfulEFOSat01-137*, cvCO213Sat04-137, cvCO285Sat04-137*, cvKASSOSat05-137*, cvNA567Sat02-137, cvPOJ28Sat02-137*, cvQ208.Sat02-137*, cvQ241.Sat03-137*, cvR570.Sat01-137*, cvRagnaSat03-137*, cvSP803Sat01-137*, MfloPI2Sat08-137*, MsinNG7Sat01-137*, SbarChuSat02-137*, SbarTEKSat03-137*, SeduE2.Sat03-137*, SeduIJ7Sat04-137*, SnarengSat04-137*, SoffBadSat03-137*, SoffBlaSat03-137*, SoffLOESat03-137*, SrobIJ7Sat03-137*, SrobNG2Sat03-137*, SsinKacSat06-137*, SsinUbaSat04-137*, Sspo196Sat02-137*, Sspo517Sat03-137*, SspoGlaSat03-137*, SspoManSat02-137*, SspoNPXSat01-137*

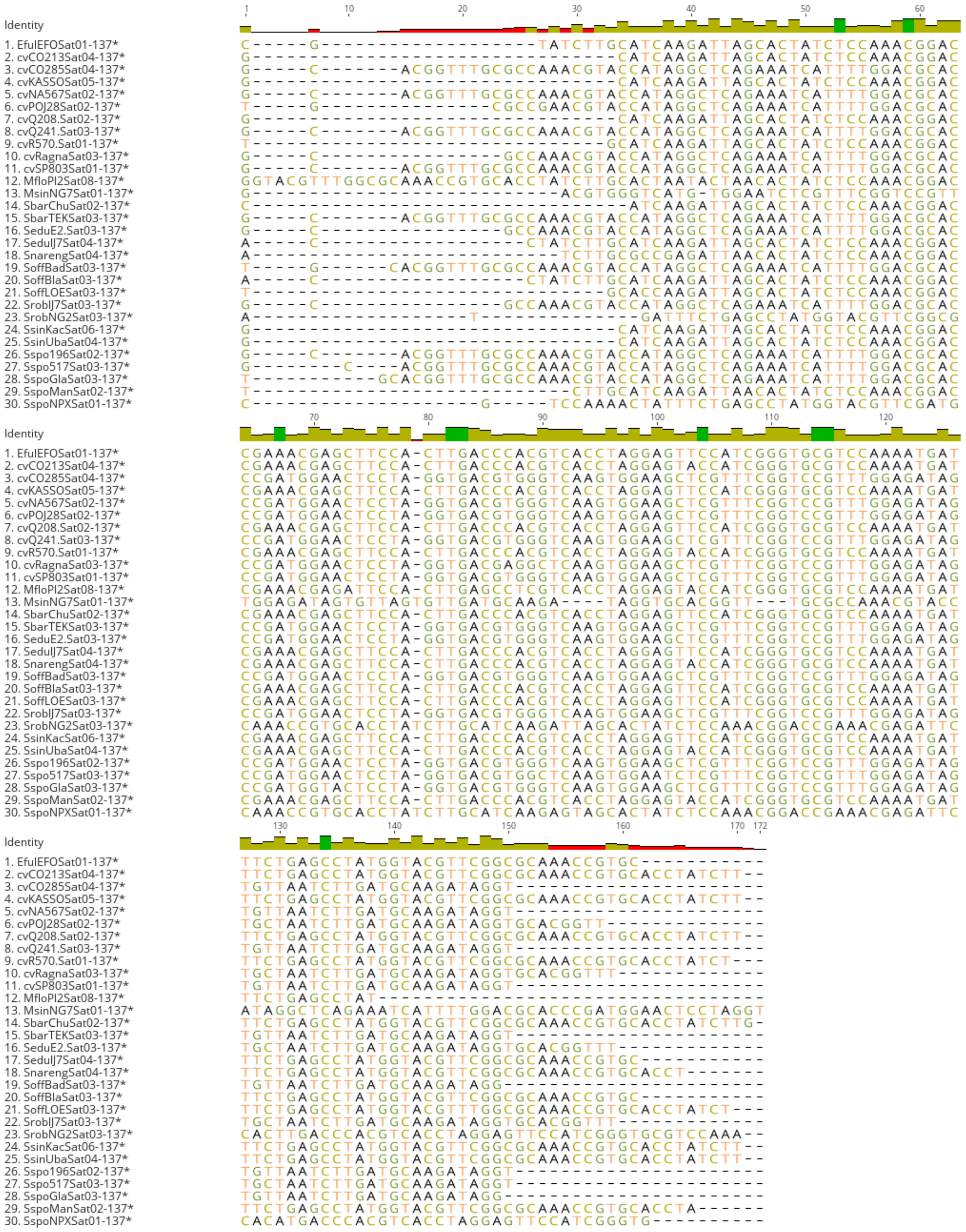

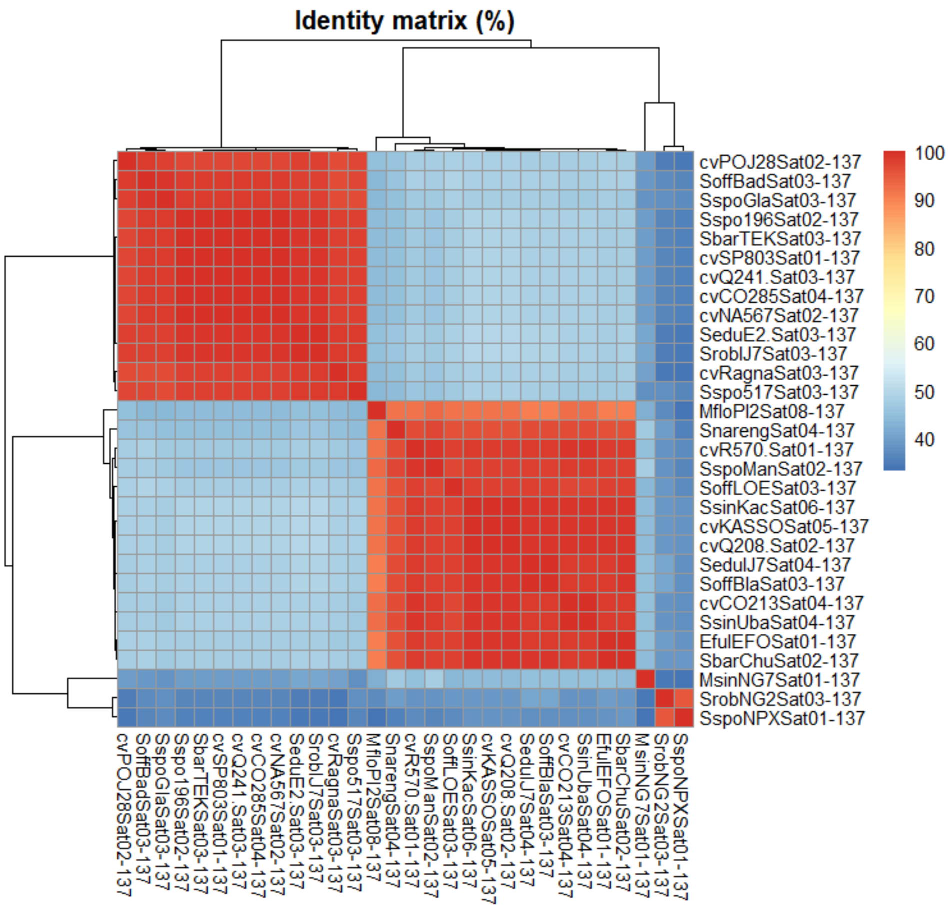

According to satDNA classification followed the criteria established by Ruiz-Ruano et al. (2016), cvPOJ28Sat02-137*, SoffBadSat03-137*, SspoGlaSat03-137*, Sspo196Sat02-137*, SbarTEKSat03-137*, cvSP803Sat01-137*, cvQ241.Sat03-137*, cvCO285Sat04-137*, cvNA567Sat02-137, SeduE2.Sat03-137*, SrobIJ7Sat03-137*, cvRagnaSat03-137*and Sspo517Sat03-137*; MfloPI2Sat08-137*, SnarengSat04-137*, cvR570.Sat01-137*, SspoManSat02-137*, SoffLOESat03-137*, SsinKacSat06-137*, cvKASSOSat05-137*, cvQ208.Sat02-137*, SeduIJ7Sat04-137*, SoffBlaSat03-137*, cvCO213Sat04-137, SsinUbaSat04-137*, EfulEFOSat01-137* and SbarChuSat02-137*; SrobNG2Sat03-137* and SspoNPXSat01-137* are variants of the same consensus monomer.

Alignment of the consensus sequence of Sat01-137 with *CENT2* (Vieira et al., 2018).

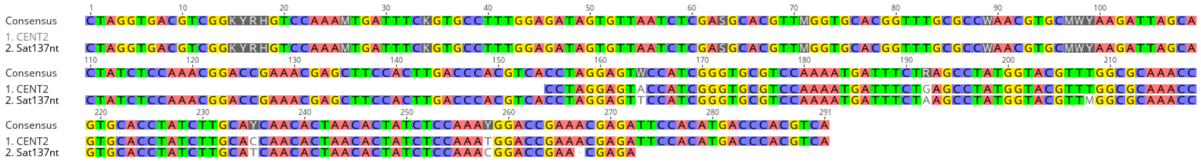

- Alignment of 10 sequences: cvKASSOSat06-144, cvSP803Sat05-144, SbarTEKSat05-144, SsinKacSat01-144*, SsinUbaSat05-144, Sspo196Sat04-144, Sspo517Sat04-144, SspoGlaSat04-144, SspoManSat03-144, SspoNPXSat04-144

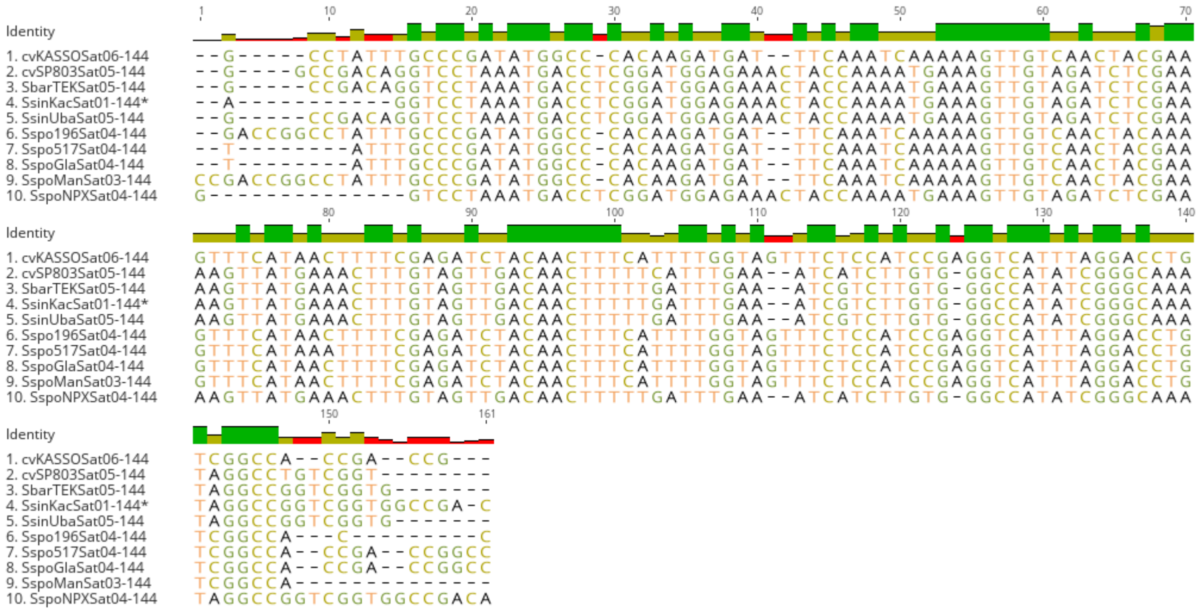

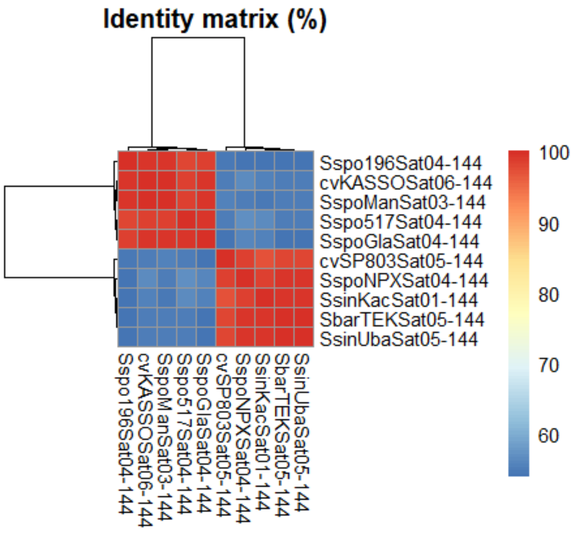

According to satDNA classification followed the criteria established by Ruiz-Ruano et al. (2016), Sspo196Sat04-144, cvKASSOSat06-144, SspoManSat03-144, Sspo517Sat04-144 and SspoGlaSat04-144; cvSP803Sat05-144, SspoNPXSat04-144, SsinKacSat01-144*, SbarTEKSat05-144, SsinUbaSat05-144 are variants of the same consensus monomer.

- Alignment of 2 sequences: SeduIJ7Sat03-185*, SnarengSat01-185*

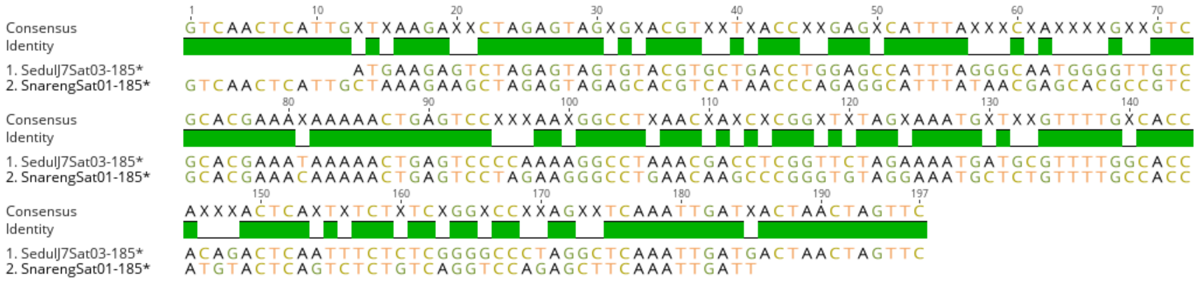

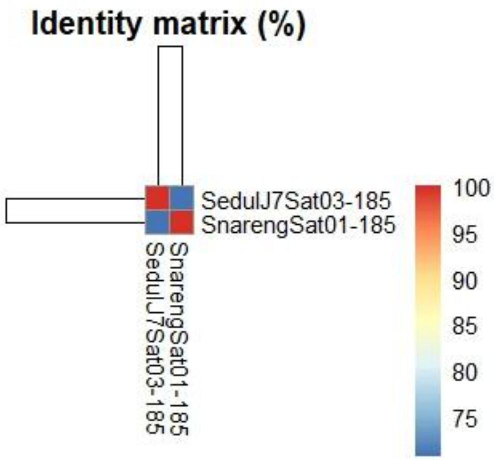

According to satDNA classification followed the criteria established by Ruiz-Ruano et al. (2016), SeduIJ7Sat03-185* and SnarengSat01-185* are different families within the same superfamily.

- Alignment of 5 sequences: cvKASSOSat04-186*, Sspo196Sat06-186, Sspo517Sat02-186*, SspoGlaSat02-186*, SspoNPXSat03-186*

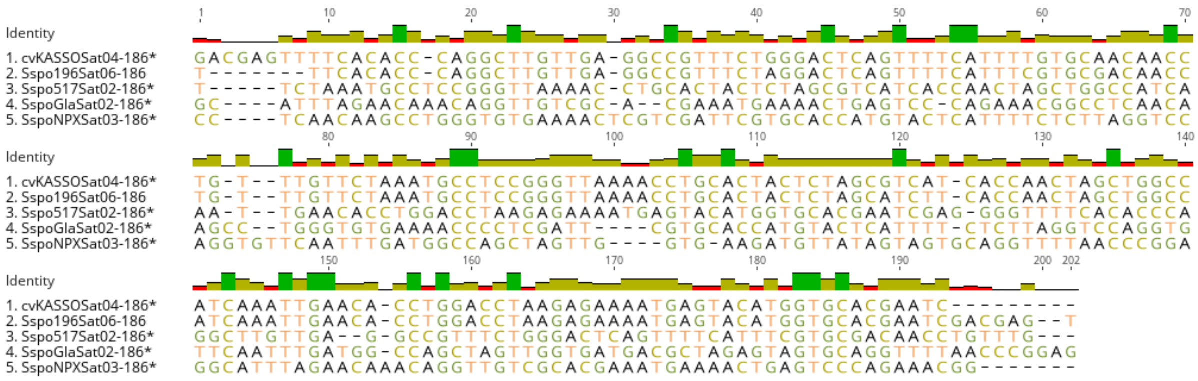

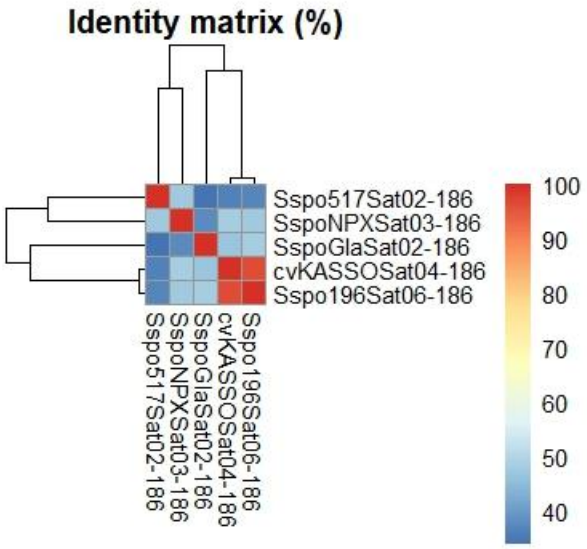

According to satDNA classification followed the criteria established by Ruiz-Ruano et al. (2016), cvKASSOSat04-186* and Sspo196Sat06-186 are variants of the same consensus monomer.

- Alignment of 23 sequences: cvCO213Sat03-364*, cvCO285Sat03-364*, cvKASSOSat03-364*, cvNA567Sat05-364, cvPOJ28Sat05-364, cvQ208.Sat04-364, cvQ241.Sat02-364*, cvR570.Sat05-364, cvRagnaSat02-364*, EfulEFOSat02-364*, SbarTEKSat06-364, SeduE2.Sat02-364*, SeduIJ7Sat02-364*, SoffBadSat02-364*, SoffLOESat02-364*, SrobIJ7Sat02-364*, SrobNG2Sat02-364*, SsinKacSat04-364*, SsinUbaSat03-364*, Sspo517Sat08-364, SspoGlaSat07-364, SspoManSat07-364, SspoNPXSat07-364.

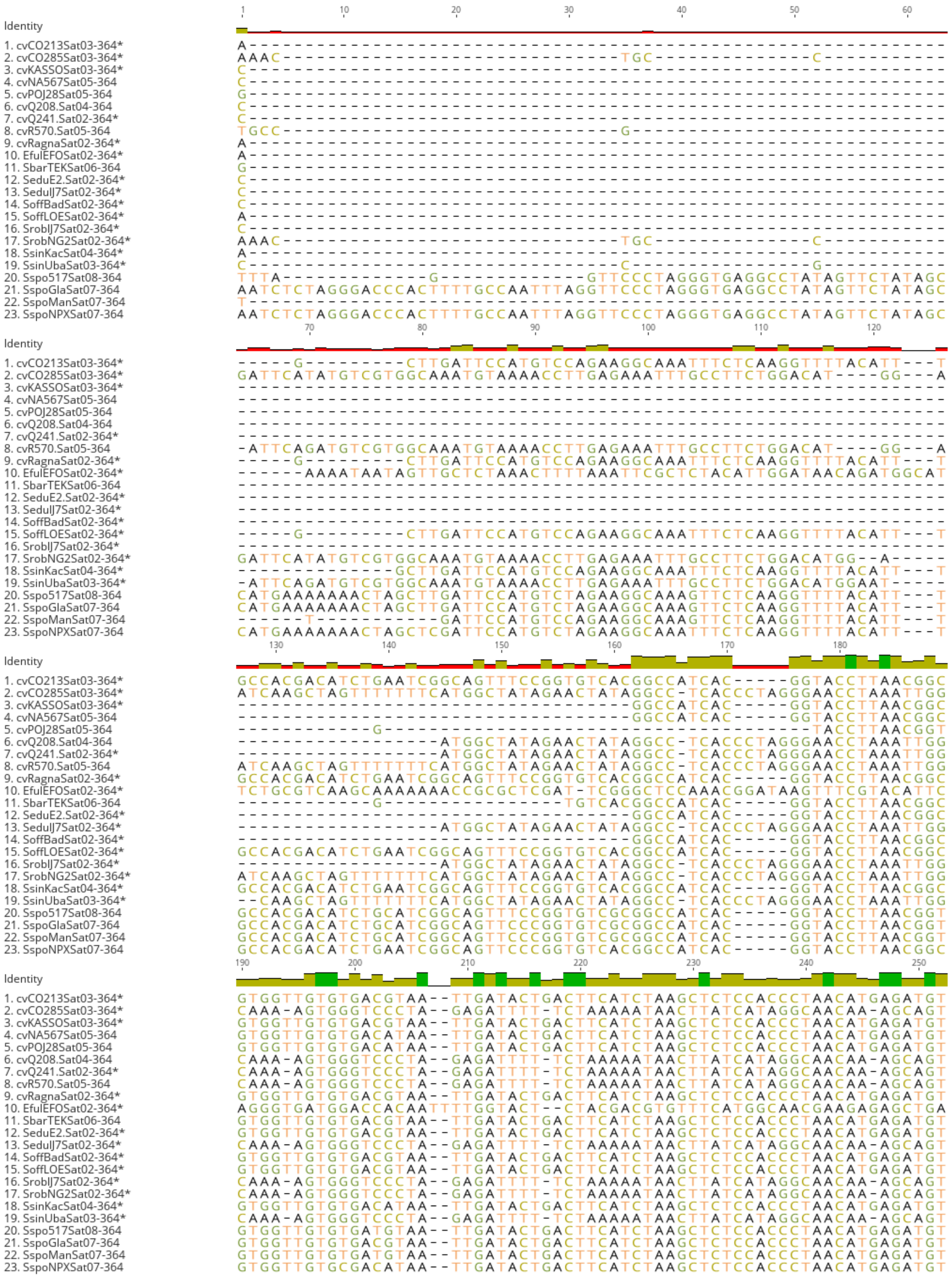

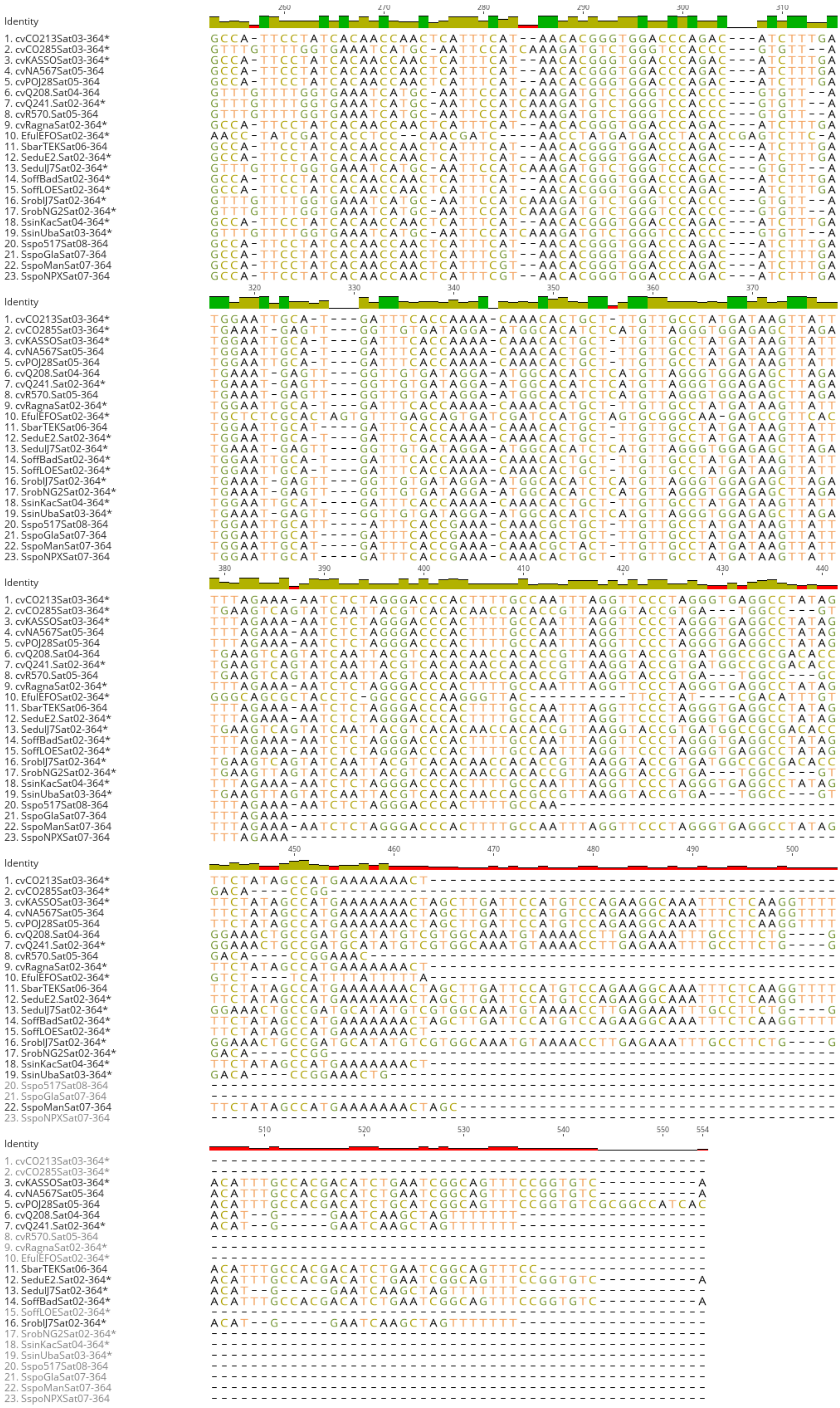
Identity
1. cvCO213Sat03-364*
2. cvCO285Sat03-364*
3. cvKASSOSat03-364*
4. cvNA567Sat05-364
5. cvPOJ28Sat05-364
6. cvQ208.Sat04-364
7. cvQ241.Sat02-364*
8. cvR570.Sat05-364
9. cvRagnaSat02-364*
10. EfulEFOSat02-364*
11. SbarTEKSat06-364
12. SeduE2.Sat02-364*
13. SeduIJ7Sat02-364*
14. SoffBadSat02-364*
15. SoffLOESat02-364*
16. SrobIJ7Sat02-364*
17. SrobNG2Sat02-364*
18. SsinKacSat04-364*
19. SsinUbaSat03-364*
20. Sspo517Sat08-364
21. SspoGlaSat07-364
22. SspoManSat07-364
23. SspoNPXSat07-364

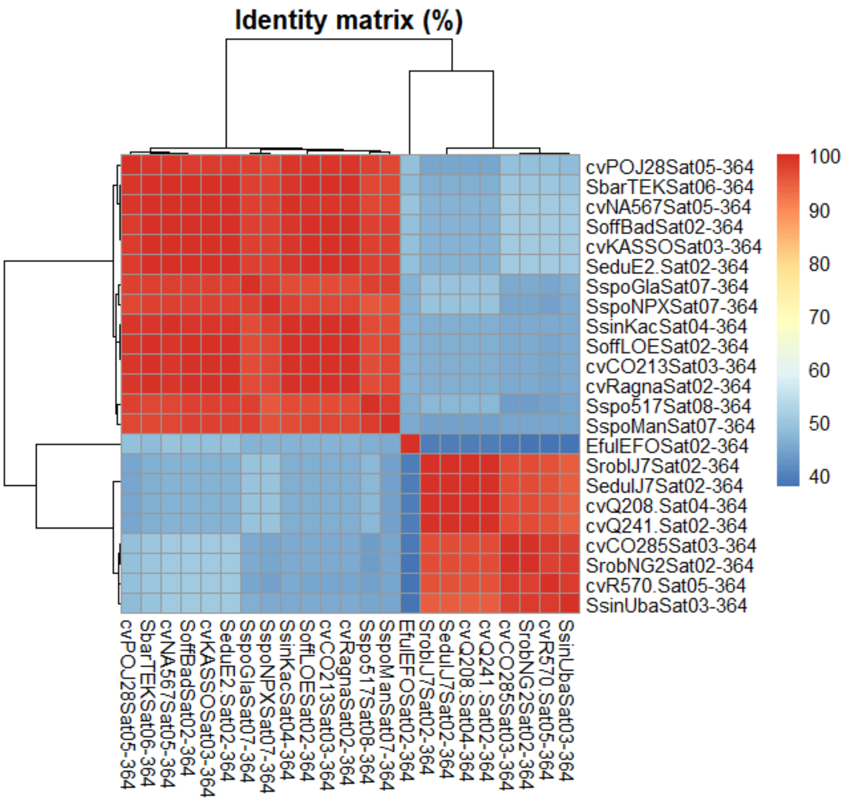

According to satDNA classification followed the criteria established by Ruiz-Ruano et al. (2016), cvPOJ28Sat05-364, SbarTEKSat06-364, cvNA567Sat05-364, SoffBadSat02-364*, cvKASSOSat03-364*, SeduE2.Sat02-364*, SspoGlaSat07-364, SspoNPXSat07-3, SsinKacSat04-364*, SoffLOESat02-364*, cvCO213Sat03-364*, cvRagnaSat02-364*, Sspo517Sat08-364 and SspoManSat07-364; SrobIJ7Sat02-364*, SeduIJ7Sat02-364*, cvQ208.Sat04-364, cvQ241.Sat02-364*, cvCO285Sat03-364*, SrobNG2Sat02-364*, cvR570.Sat05-364 and SsinUbaSat03-364*, are variants of the same consensus monomer.

- Alignment of 24 sequences: cvCO213Sat02-365*, cvCO285Sat01-365*, cvKASSOSat02-365*, cvNA567Sat01-365*, cvPOJ28Sat01-365*, cvQ208.Sat01-365*, cvQ241.Sat01-365*, cvR570.Sat02-365, cvRagnaSat01-365*, cvSP803Sat02-365*, SbarChuSat01-365*, SbarTEKSat02-365*, SeduE2.Sat01-365*, SeduIJ7Sat01-365*, SnarengSat09-365, SoffBadSat01-365*, SoffBlaSat01-365*, SoffLOESat01-365*, SrobIJ7Sat01-365*, SrobNG2Sat01-365*, SsinKacSat03-365*, SsinUbaSat02-365*, Sspo196Sat03-365, SspoNPXSat02-365*.

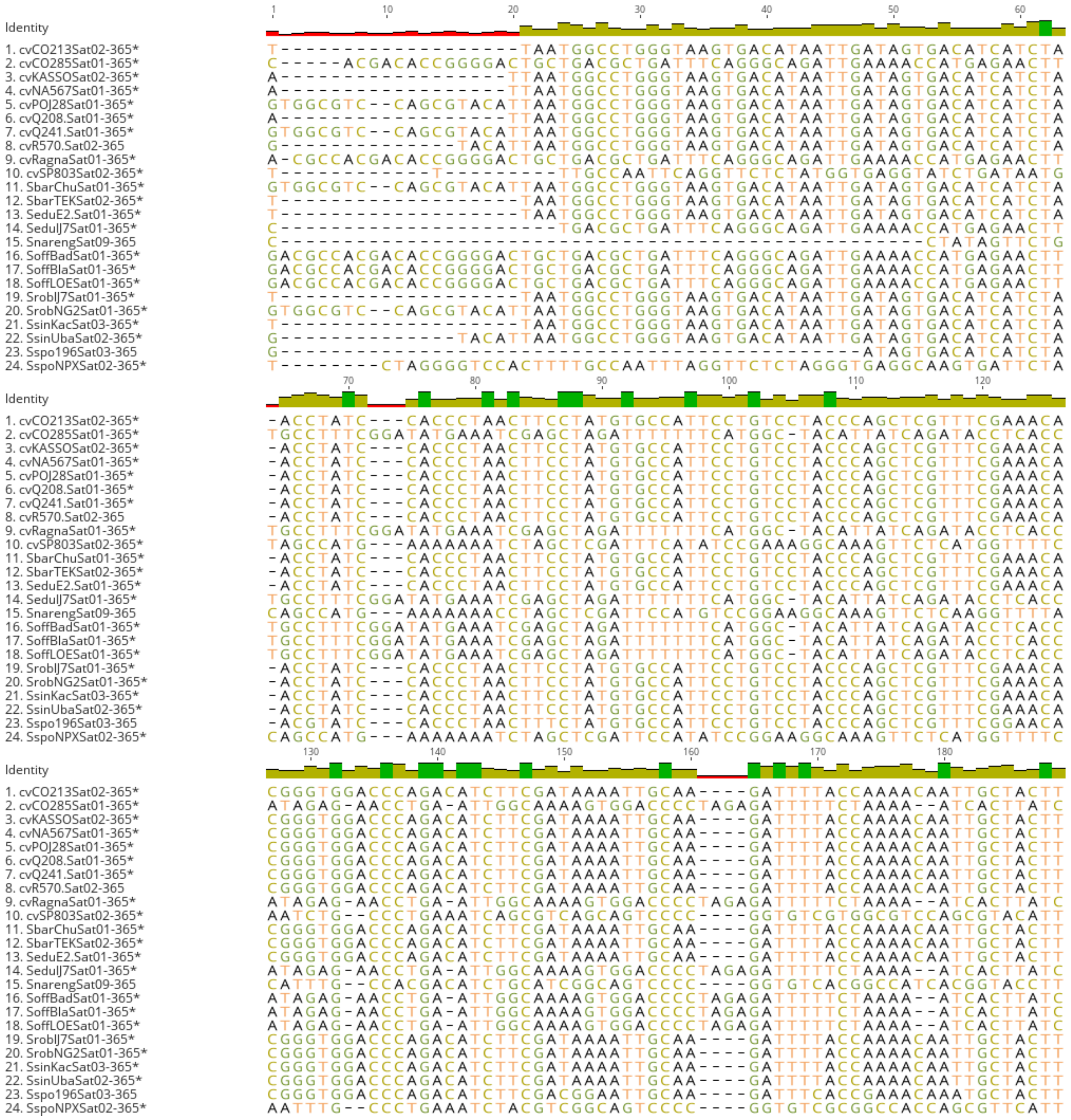

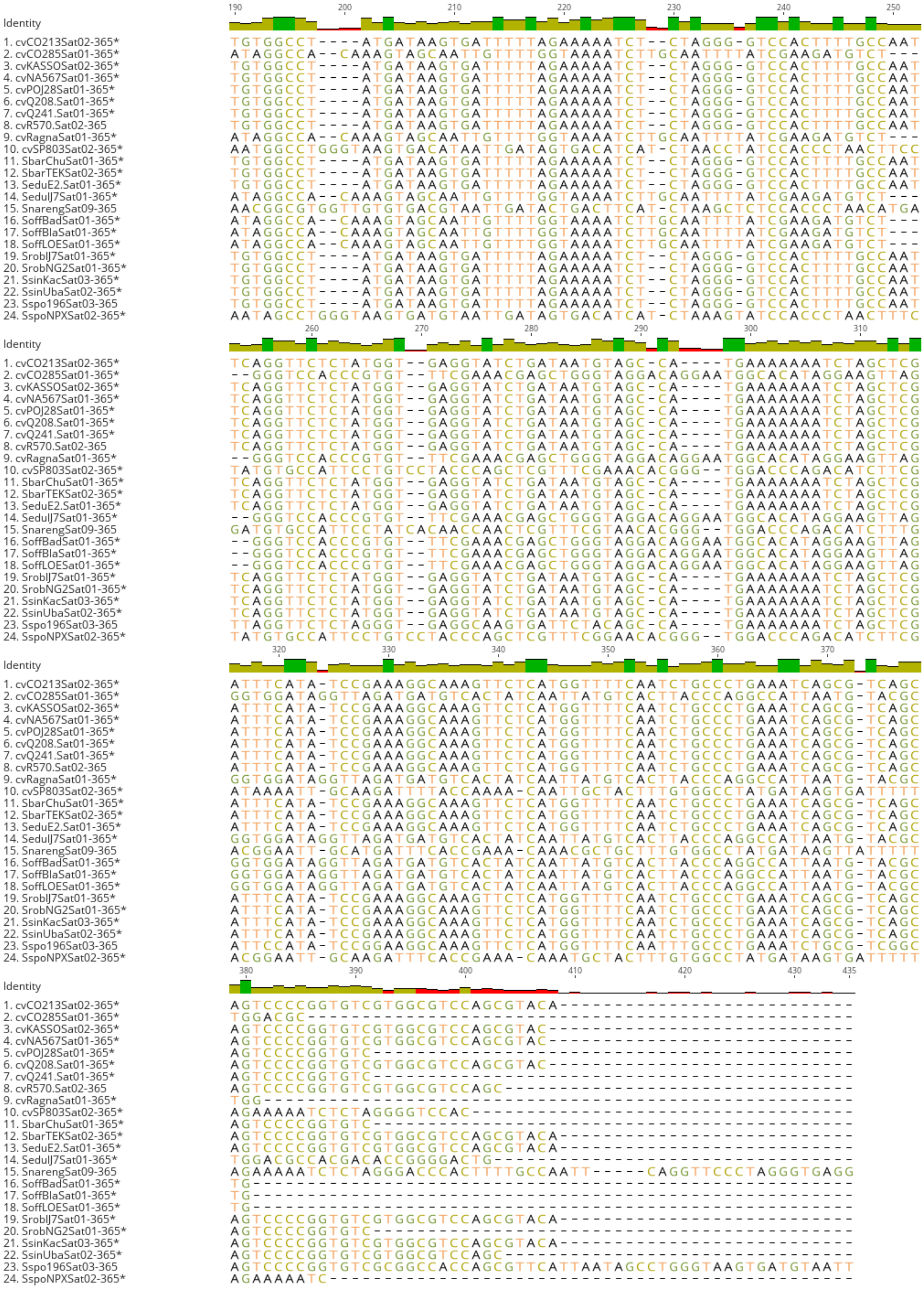

Identity
190 200 210 220 230 240 250
260 270 280 290 300 310
320 330 340 350 360 370
380 390 400 410 420 430 435
1. cvCO213Sat02-365*
2. cvCO285Sat01-365*
3. cvKASSOSat02-365*
4. cvNA567Sat01-365*
5. cvPOJ28Sat01-365*
6. cvQ208.Sat01-365*
7. cvQ241.Sat01-365*
8. cvR570.Sat02-365
9. cvRagnaSat01-365*
10. cvSP803Sat02-365*
11. SbarChuSat01-365*
12. SbarTEKSat02-365*
13. SeduE2.Sat01-365*
14. SeduIJ7Sat01-365*
15. SnarengSat09-365
16. SoffBadSat01-365*
17. SoffBlaSat01-365*
18. SoffLOESat01-365*
19. SrobIJ7Sat01-365*
20. SrobNG2Sat01-365*
21. SsinKacSat03-365*
22. SsinUbaSat02-365*
23. Sspo196Sat03-365
24. SspoNPXSat02-365*

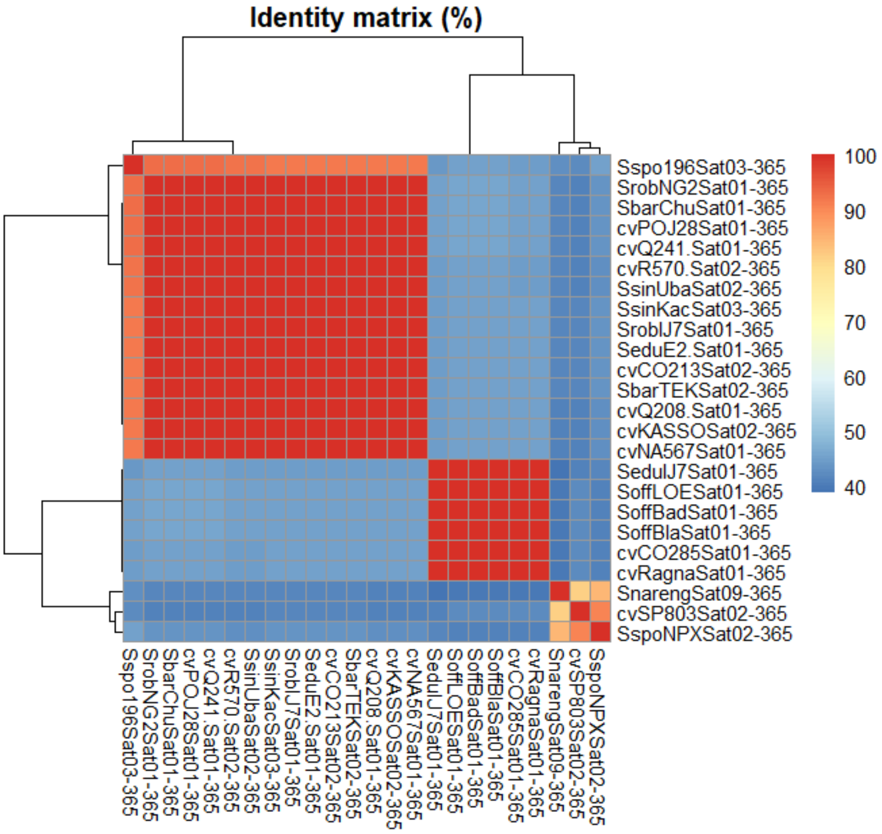

According to satDNA classification followed the criteria established by Ruiz-Ruano et al. (2016), Sspo196Sat03-365, SrobNG2Sat01-365*, SbarChuSat01-365*, cvPOJ28Sat01-365*, cvQ241.Sat01-365*, cvR570.Sat02-365, SsinUbaSat02-365*, SsinKacSat03-365*, SrobIJ7Sat01-365*, SeduE2.Sat01-365*, cvCO213Sat02-365*, SbarTEKSat02-365*, cvQ208.Sat01-365*, cvKASSOSat02-365* and cvNA567Sat01-365*; SeduIJ7Sat01-365*, SoffLOESat01-365*, SoffBadSat01-365*, SoffBlaSat01-365*, cvCO285Sat01-365* and cvRagnaSat01-365*, are variants of the same consensus monomer. SnarengSat09-365, cvSP803Sat02-365* and SspoNPXSat02-365* are variants belonging to the same family.

- Alignment of 2 sequences: MfloPI2Sat02-368*, MsinNG7Sat04-368*

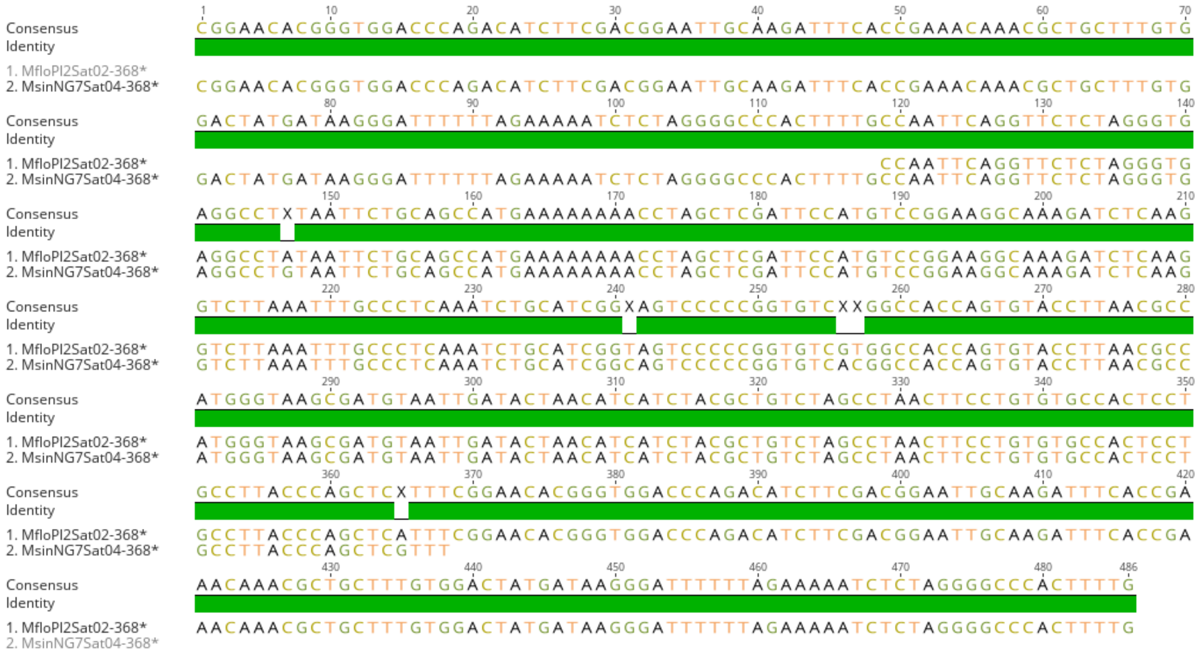

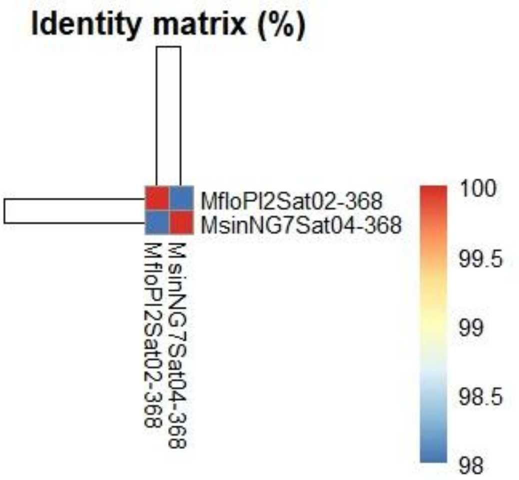

According to satDNA classification followed the criteria established by Ruiz-Ruano et al. (2016), MfloPI2Sat02-368* and MsinNG7Sat04-368* are variants of the same consensus monomer.

- Alignment of 3 sequences: SbarTEKSat01-513*, SsinKacSat02-513*, SsinUbaSat01-513*

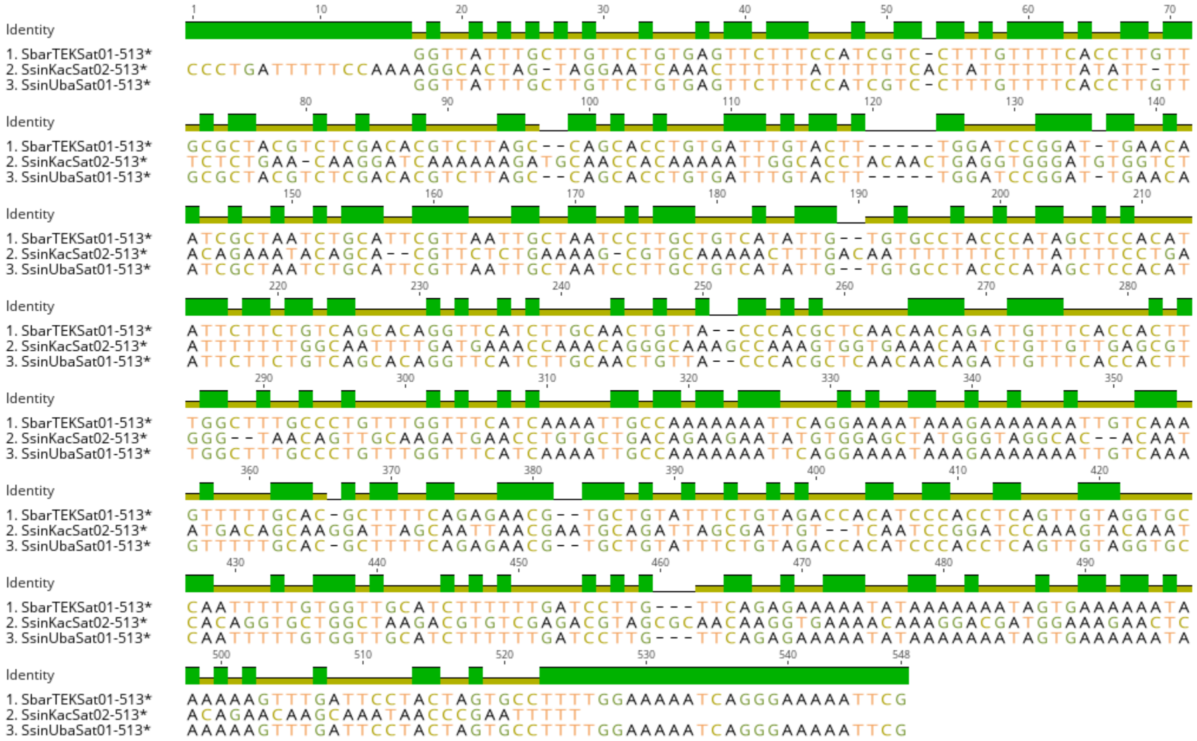

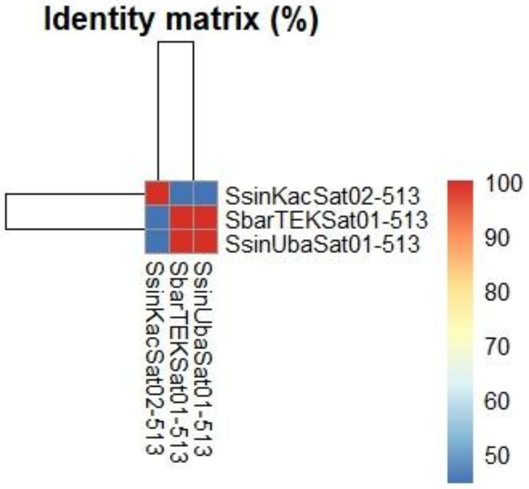

According to satDNA classification followed the criteria established by Ruiz-Ruano et al. (2016), SbarTEKSat01-513*, SsinKacSat02-513* are variants of the same consensus monomer.

- Alignment of 4 sequences: cvQ208.Sat03-515, cvR570.Sat04-515, SbarChuSat04-515, Sspo196Sat01-515*

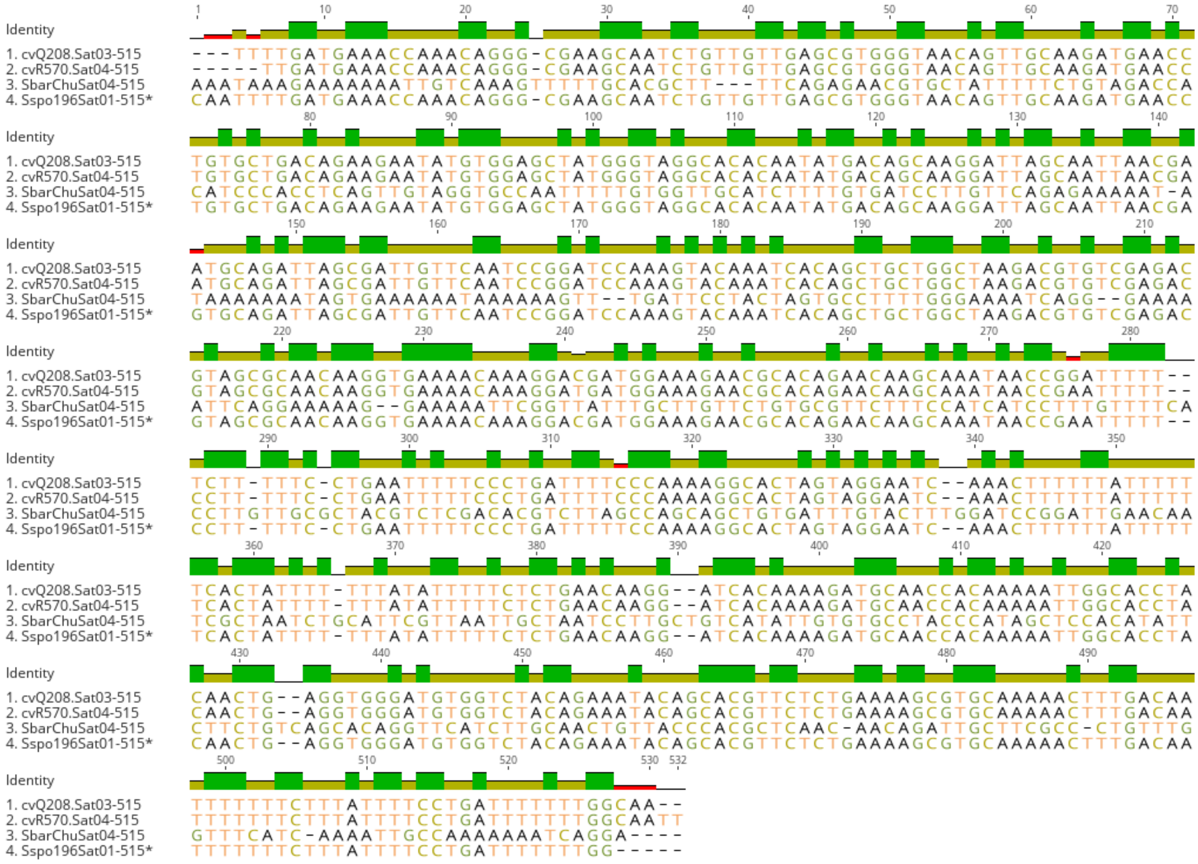

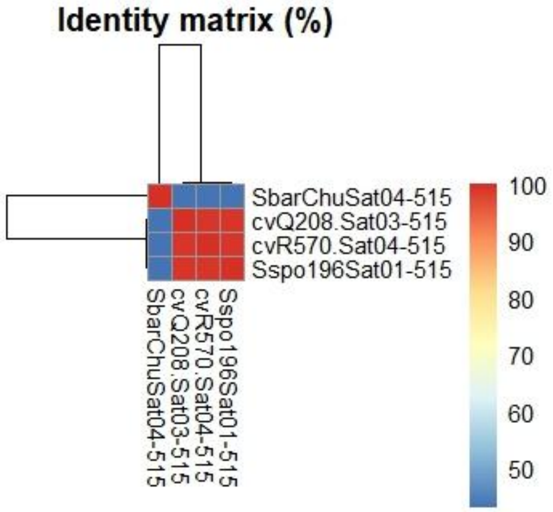

According to satDNA classification followed the criteria established by Ruiz-Ruano et al. (2016), cvQ208.Sat03-515, cvR570.Sat04-515, Sspo196Sat01-515* are variants of the same consensus monomer.

- Alignment of 3 sequences: cvCO213Sat01-516*, cvCO285Sat02-516*, cvRagnaSat05-516

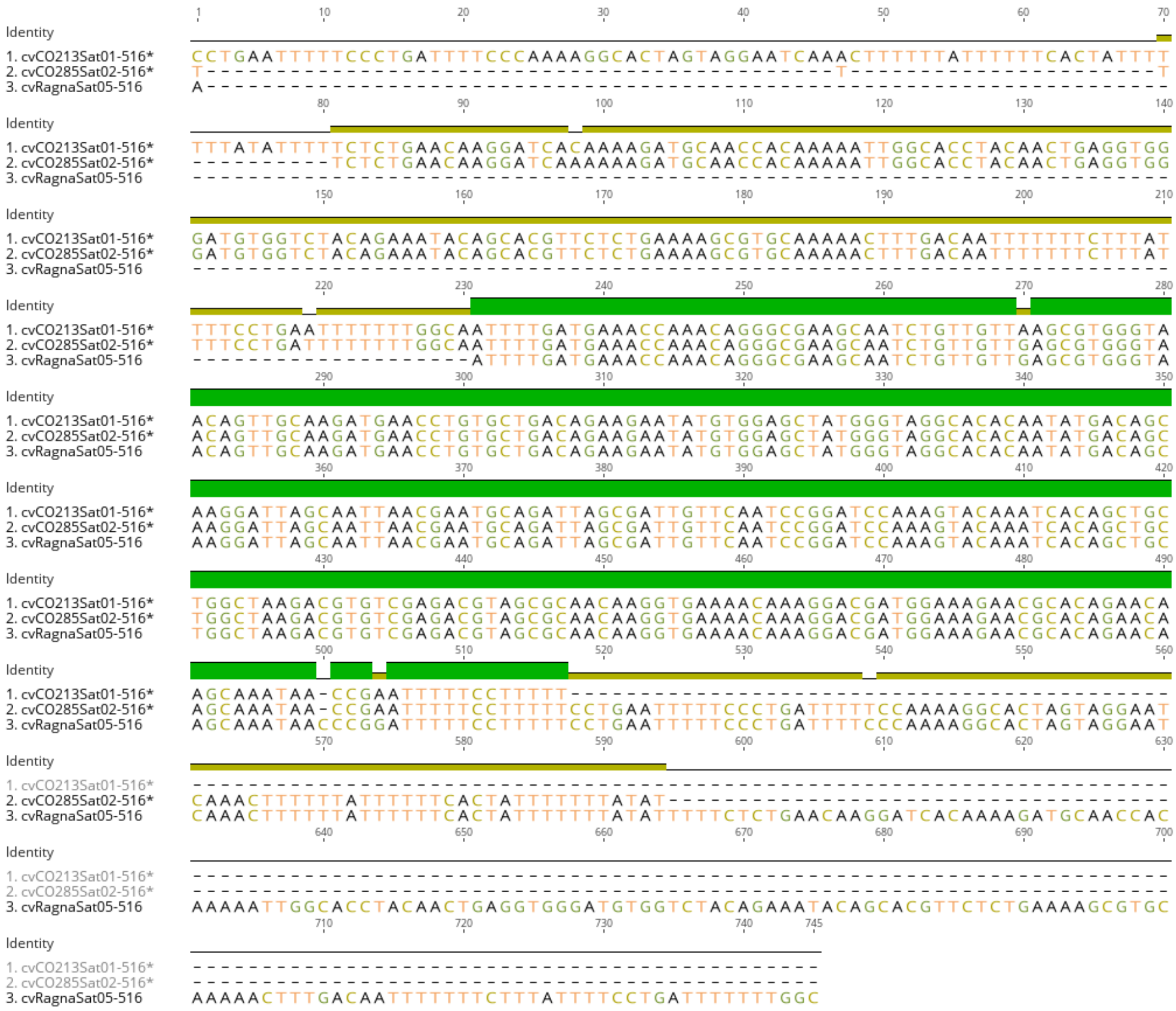

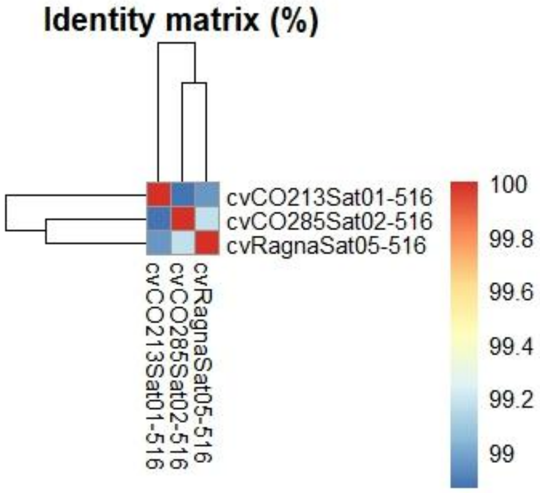

According to satDNA classification followed the criteria established by Ruiz-Ruano et al. (2016), cvCO213Sat01-516*, cvCO285Sat02-516*, cvRagnaSat05-516 are variants of the same consensus monomer.

- Alignment of 3 sequences: cvKASSOSat01-531*, Sspo517Sat01-531*, SspoGlaSat01-532*

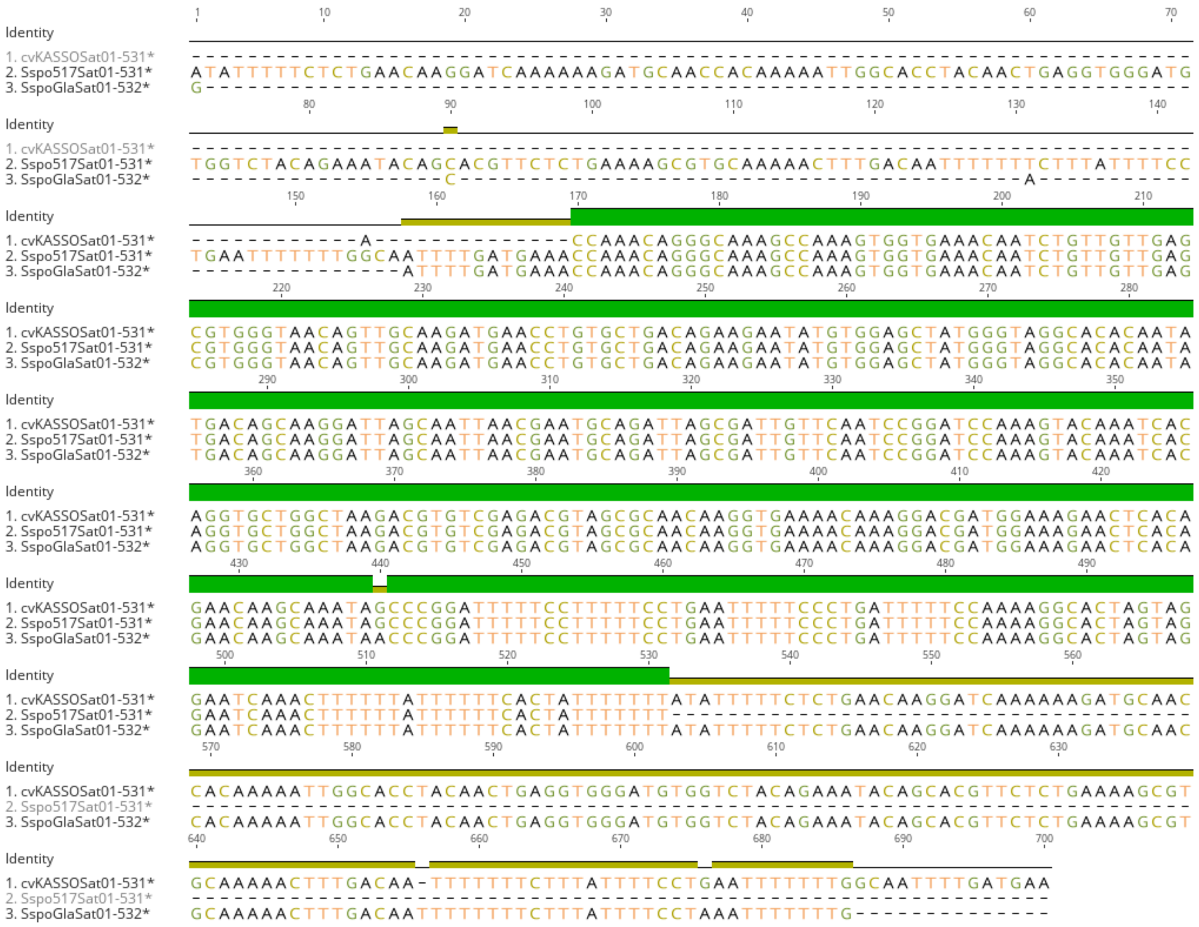

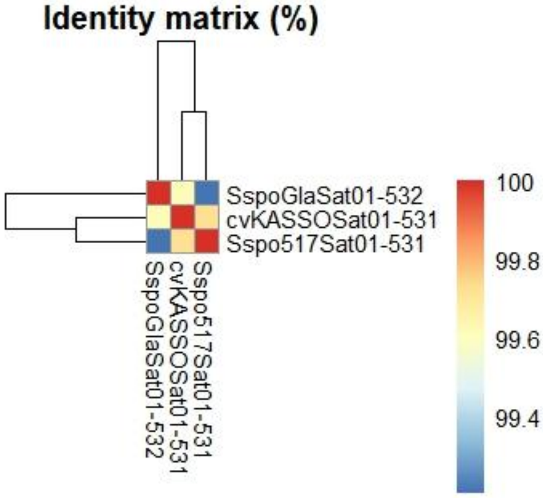

According to satDNA classification followed the criteria established by Ruiz-Ruano et al. (2016), SspoGlaSat01-532*, cvKASSOSat01-531*, Sspo517Sat01-531* are variants of the same consensus monomer.
